# Gut fungi enhances immunosuppressive function of myeloid-derived suppressor cells by activating PKM2-dependent glycolysis to promote colorectal tumorigenesis

**DOI:** 10.1186/s40164-022-00334-6

**Published:** 2022-11-08

**Authors:** Zhiyong Zhang, Yaojun Zheng, Ying Chen, Yuxin Yin, Yuxi Chen, Qianyu Chen, Yayi Hou, Sunan Shen, Mingming Lv, Tingting Wang

**Affiliations:** 1 The State Key Laboratory of Pharmaceutical Biotechnology, Division of Immunology, Medical School, Nanjing University, Nanjing, 210093 China; 2 Jiangsu Key Laboratory of Molecular Medicine, Division of Immunology, Medical School, Nanjing University, Nanjing, 210093 China; 3 Department of Breast, Nanjing Maternity and Child Health Care Hospital, Women’s Hospital of Nanjing Medical University, Nanjing, 210004 China

**Keywords:** *Candida tropicalis*, Colorectal cancer, Myeloid-derived suppressor cells, Syk, PKM2, Aerobic glycolysis

## Abstract

**Background:**

Accumulating evidence implicates that gut fungi are associated with the pathogenesis of colorectal cancer (CRC). Our previous study has revealed that *Candida tropicalis* (*C. tropicalis*) promotes colorectal tumorigenesis by enhancing immunosuppressive function of myeloid-derived suppressor cells (MDSCs) and increasing accumulation of MDSCs, but the underlying mechanisms remain unestablished.

**Methods:**

Bone marrow–derived MDSCs were stimulated with *C. tropicalis*. RNA-sequencing analysis was performed to screen the differentially expressed genes. Quantitative real-time PCR and western blot were used to measure the expression of related proteins. Co-culture assay of MDSCs and CD8^+^ T cells was used to determine the immunosuppressive ability of MDSCs. Metabolomic analysis was conducted to detect metabolic reprogramming of MDSCs. Aerobic glycolysis of MDSCs was assessed by extracellular acidification rate (ECAR), glucose consumption and lactate production. A CAC mouse model was induced by AOM and DSS to determine the therapeutic action of TEPP-46. IHC and immunofluorescence were performed to examine the expression of PKM2, PKM2 (p-Y105) and iNOS in human CRC-infiltrated MDSCs.

**Results:**

*C. tropicalis* facilitates immunosuppressive function of MDSCs by increasing the expression of iNOS, COX2 and NOX2, production of nitric oxide (NO) and reactive oxygen species (ROS). Mechanistically, *C. tropicalis* facilitates the immunosuppressive function of MDSCs through the C-type lectin receptors Dectin-3 and Syk. *C. tropicalis*-enhanced immunosuppressive function of MDSCs is further dependent on aerobic glycolysis. On the one hand, NO produced by MDSCs enhanced aerobic glycolysis in a positive feedback manner. On the other hand, *C. tropicalis* promotes p-Syk binding to PKM2, which results in PKM2 Tyr105 phosphorylation and PKM2 nuclear translocation in MDSCs. Nuclear PKM2 interacts with HIF-1α and subsequently upregulates the expression of HIF-1α target genes encoding glycolytic enzymes, GLUT1, HK2, PKM2, LDHA and PDK1, which are required for the *C. tropicalis*-induced aerobic glycolysis of MDSCs. Blockade of PKM2 nuclear translocation attenuates *C. tropicalis*-mediated colorectal tumorigenesis. The high expression of PKM2, PKM2 (p-Y105) and iNOS in CRC-infiltrated MDSCs correlates with the development of human CRC.

**Conclusion:**

*C. tropicalis* enhances immunosuppressive function of MDSCs via Syk-PKM2-HIF-1α-glycolysis signaling axis, which drives CRC. Therefore, we identify the Syk-PKM2-HIF-1α-glycolysis signaling axis as a potential therapeutic target for CRC.

**Supplementary Information:**

The online version contains supplementary material available at 10.1186/s40164-022-00334-6.

## Background

Colorectal cancer (CRC), a severe hazard to human health, is characterized by high occurrence and death globally [1]. Recently, younger populations have seen an increase in the incidence rate and mortality of colorectal cancer [2]. Inflammatory bowel disease (IBD), which has been linked to poor quality of life, includes crohn’s disease and ulcerative colitis [3]. The risk of CRC is higher in those with ulcerative colitis [4]. As a consequence, examining the connection between IBD and the development of CRC may lead to the development of brand-new therapeutic approaches for the management of colon cancer.

Dysbiosis of the gut bacteria has been linked in several earlier studies to the onset and progression of colitis and CRC [5, 6]. IBD development also involves fungal microbiome dysbiosis in addition to bacteria [7]. Additionally, in mice lacking Dectin-1, the abundance of opportunistic pathogenic fungi, *Candida tropicalis* (*C. tropicalis*), considerably increased during DSS-induced colitis, aggravating the condition [8]. Consistently, our earlier research showed that *C. tropicalis* prevalence was increased in colitis-prone Dectin-3-deficient mice, which exacerbated colitis [9]. And more recently, we discovered that *C. tropicalis* promoted colitis-associated colon cancer (CAC) through enhancing the accumulation and immunosuppressive activity of myeloid-derived suppressor cells (MDSCs) [10]. Unknown, however, are the exact molecular pathways by which *C. tropicalis* enhances MDSCs accumulation and immunosuppressive activity.

Immature bone marrow cells and myeloid progenitor cells make up the heterogeneous cell population known as (MDSCs) [11]. Tumor-bearing mice are the initial hosts of MDSC discovery and they co-express CD11b and Gr-1. Granulocytic MDSCs (G-MDSCs) and monocytic MDSCs are the two current populations of MDSCs [12]. MDSCs build up during tumor, autoimmune disease and infection, effectively inhibiting T cell activity [13]. With high levels of inducible nitric oxide synthase (iNOS), arginase-1 (Arg-1), cyclooxygenase 2 (COX2), NADPH oxidase 2 (NOX2) and production of reactive oxygen species (ROS), nitric oxide (NO) and so on, these cells inhibit T cell function and activation [12, 14].

The pattern recognition receptors (PPRs) known as C-type lectin receptors (CLRs) comprising Dectin-1, Dectin-2, and Dectin-3, play a key role in mediating antifungal immunity [15, 16]. The majority of myeloid cells, including macrophages and dendritic cells, express CLRs on their surface. They have the ability to sense the components of fungal cell walls, β-glucans, a-mannans and so on. It’s interesting to note that β-glucans, α-mannans and chitin are present in both the cell wall structure and composition of *C. tropicalis* and *C. albicans* [17]. Dectin-3 and Dectin-2 were discovered to form a heterodimeric PRR for detecting fungal infection [18]. Additionally, we discovered that *C. tropicalis* could be recognized by Dectin-3 on macrophages [9, 19]. Whereas, it is still unclear how Dectin-3 modulate the immunosuppressive function of MDSCs in the development of CRC remains ambiguous.

A rising number of research efforts in recent years have demonstrated that metabolic reprogramming is essential for immune cell function. [20–25]. The destiny and functionality of immune cells are manipulated by this metabolism-driven reprogramming. The two primary anabolic and catabolic processes are glycolysis and oxidative phosphorylation (OXPHOS). The “Warburg effect,“ for instance, describes how tumor cells employ aerobic glycolysis to generate high quantities of ATP even when oxygen is present. [26]. To our surprise, a recent study found that cancer cells had the highest glutamine absorption while myeloid cells had the highest intratumoral glucose uptake. [27]. This appears to be inconsistent with earlier findings. By chance, studies also revealed that immune cells in tumors mostly used glucose metabolism. Glycolysis could be used by MDSCs in particular to promote their immunosuppressive activity in malignancies [28, 29]. However, it is still unclear whether *C. tropicalis* may cause MDSCs to undergo metabolic reprogramming, and the underlying mechanism has to be investigated.

In the current study, we first report that Syk-dependent PKM2 Tyr105 phosphorylation and PKM2 nuclear translocation are necessary for expression of HIF-1α target genes encoding glycolytic enzymes, which are required for the *C. tropicalis*- induced glycolysis activation in MDSCs. Subsequently, glycolysis activation results in enhanced immunosuppressive function of MDSCs, thus exacerbating progression of CRC. Therefore, we elucidate an as-yet-unknown mechanism by which *C. tropicalis* promotes the development of CRC and pinpoint the Syk-PKM2-HIF-1α-glycolysis signaling axis as a promising therapeutic target for the disease.

## Methods

### Reagents

2-Deoxy-D-glucose (2-DG) (#D8375) and NO donor S-nitroso-N-acetylpenicillamine (SNAP, #N3398) were purchased from Sigma-Aldrich. iNOS inhibitor S-methylisothiourea hemisulfate salt (SMT, #s0008) were obtained from Beyotime Biotechnology. Syk inhibitor R406 (#HY-12,067) and TEPP-46 (ML265, #S7302) were purchased from MedChemExpress.

### Mice

*Clec4d* ^−/−^ C57BL/6 J mice were friendly provided by Dr. Xin Lin (Tsinghua University, Beijing, China). *Clec4d*^−/−^ mice and WT controls (*Clec4d*^+/+^) used in this study were from heterozygous *Clec4d*^+/−^ mice breeding set-ups. And 8–10 weeks old female mice were utilized throughout the entirety of the experiments. At the Medical School of Nanjing University, all mice were kept in a pathogen-free environment. Animal care and experimental protocols were in accordance with the NIH “Guide for the Care and Use of the Laboratory Animal.” All animal study were performed following a protocol reviewed and approved by the Institutional Animal Care and Use Committee at Nanjing University (SCXK-Jiangsu-2019-0056).

### Fungal strains

The *C. tropicalis* strain (W4162870) was friendly provided by Dr. Xin Lin (Tsinghua University, Beijing, China) and was stored in 35% glycerol at − 80 ℃. It grown normally on Sabouraud (Sab) agar plates at 25 ℃. Then a single *C. tropicalis* colony was grown at 30 °C in Sab broth (1% mycological peptone and 4% glucose) overnight. The number of *C. tropicalis* was measured by a cell counter. Heat-inactivated *C.tropicalis* was prepared by heat treatment of the cell suspension at 95 °C for 45 min.

### Mouse CAC model

To establish CAC model, female WT C57BL/6 J mice (8–10 weeks old) were intraperitoneally injected with one dose of AOM (10 mg/kg; Sigma; #A5486) on day 1. After 5 days, the mice were fed with drinking water containing 2% DSS (36–50 kDa; MP Biomedicals, #160,110) for 7 consecutive days. 3 cycles of 2% DSS administration in water were performed. The mice were orally gavaged with *C. tropicalis* (1 **×** 10^7^ yeast/mouse/dose, twice a week) and TEPP-46 (50 mg/kg) was injected intraperitoneally once a week during induction of tumorigenesis. The mice were euthanized on day 100. All colon tumor tissues and spleens were removed and collected on day 100.

### Bone marrow–derived MDSCs preparation

Primary cultures of BM–derived MDSCs from female WT and *Clec4d*^−/−^ C57BL/6 J mice were prepared as previously [30, 31]. Briefly, mice’s tibias and femurs were used to extract bone marrow cells. Using an ACK Lysis Buffer, erythrocytes were eliminated. In complete RPMI-1640 medium with 40 ng/ml mouse IL-6 (Miltenyi Biotec, #130-096-683) and 40 ng/ml mouse GM-CSF (Miltenyi Biotec, #130-095-746), the cells were cultured for 4 d. After 4 days of culture, flow cytometry analysis demonstrated that the purity of MDSCs (CD11b^+^Gr1^+^) was nearly 90% (Additional file 1: Fig. S1B).

### RNA sequencing

RNA–seq analysis was conducted by OE Biotech Co., Ltd. (Shanghai, China). Briefly, total RNA was isolated from TRIzol Reagent (Invitrogen, Carlsbad, CA) from MDSCs treated with or without *C. tropicalis* according to the manufacturer’s instructions. By using the NanoDrop 2000 spectrophotometer (Thermo Scientific, USA), RNA purity and quantification were examined. The Agilent 2100 Bioanalyzer (Agilent Technologies, Santa Clara, CA, USA) was used to determine the integrity of RNA. Then, TruSeq Stranded mRNA LT Sample Prep Kit (Illumina, San Diego, CA, USA) was used to create the libraries following the manufacturer’s instructions. Paired-end reads were produced after the libraries were sequenced on the Illumina HiSeq X Ten platform. Using HISAT2, the clean reads were mapped to the most current mouse reference genome. The read counts for each gene were acquired using HTSeq-count, and the FPKM of each gene was determined using Cufflinks. The DESeq (2012) R package was used to carry out the differential expression analysis. The threshold for substantially differential expression was chosen at P value < 0.05 and fold change > 2 or fold change < 0.5. Heat maps were produced in R using the gplots package.

### cDNA synthesis and quantitative real-time PCR

Following the manufacturer’s recommendations, total RNA was extracted using the TRIzol Reagent (Invitrogen, Carlsbad, CA), and using oligo (dT) primer, it was reverse-transcribed into cDNA. On a Step One Plus or an ABI Vii 7 sequence detection system (Applied Biosystems, Thermo Fisher Scientific, US), Q-PCR test was carried out using SYBR green PCR master mix solution. Relative abundance of genes was calculated with 2^−ΔΔCT^ quantification method and β-actin was used as internal control. The primers used are listed in Additional file 1: Table S1.

### Western blot analysis and immunoprecipitation

Collected MDSCs and colon tumor tissue were lysed in lysis buffer. Protein concentration was measured after cell lysis. Total cell lysates or nuclear extracts were put through SDS–PAGE and then blotted.

Cell lysates from MDSCs were immunoprecipitated with PKM2 Rabbit mAb (Sepharose® Bead Conjugate) or Rabbit mAb IgG Isotype Control (Sepharose® Bead Conjugate). The resulting immunoprecipitates and total lysates were subjected to SDS-PAGE and followed by the immunoblotting with corresponding antibodies. The antibodies used are listed in Additional file 1: Table S2. Fully uncropped and unprocessed membrane blot are shown in Additional file 2.

### Flow cytometry

Flow cytometric analyses were performed as described previously [31]. In brief, for cell surface marker staining, single cell suspensions were prepared from different cell samples or organs. Then single cells were stained with fluorescence-conjugated anti-CD45, anti-CD11b, anti-Gr-1, anti-Ly6G, anti-Ly6C, anti-CD3, anti-CD4, anti-CD8a and 7-AAD (BD Pharmingen, 559,925) for 30 min at 4 °C in the dark. To detect the expression of PKM2, PKM2 (p-Y105) and iNOS in MDSCs (HLA-DR^−/low^ CD33^+^ CD14^−/low^ CD66b^+^) from human CRC tissues and adjacent nontumor tissues, the single cell suspensions from human CRC tissues and adjacent nontumor tissues were stained with fluorescence-conjugated anti-human HLA-DR antibody, anti-human CD33 antibody, anti-human CD14 antibody and anti-human CD66b antibody, or anti-PKM2 (CST), anti-PKM2 (p-Y105) (Bioss), anti-iNOS (Proteintech) respectively. Subsequently, flow cytometry was carried out on a FACSCalibur flow cytometer (BD Biosciences). The data was analyzed using the FlowJo sofware. The antibodies used are listed in Additional file 1: Tables S3 and S2.

### Measurement of nitric oxide (NO) concentration

MDSCs were treated and then the concentration of nitric oxide (NO) in cell culture supernatants was measured using a nitric oxide (NO) assay kit (Beyotime Biotechnology, S0021) based on the Griess reaction. All samples were prepared and measured according to the manufacturer’s protocol.

### Detection of reactive oxygen species production (ROS)

Reactive oxygen species (ROS) production by MDSCs was assayed by using oxidation-sensitive dye DCFH-DA (Beyotime Biotechnology, S0033S) according to the manufacturer’s protocol. In brief, MDSCs were collected and then incubated at 37 °C in RPMI-1640 medium containing 10 µM DCFH-DA for 30 min. After the incubation, MDSCs were washed with RPMI-1640 medium to remove nonspecific binding. Then the relative amount of ROS generated was assayed by flow cytometry.

### MDSC suppression assay

BM–derived MDSCs from female WT and *Clec4d*^−/−^ C57BL/6 J mice were prepared. Next, WT and *Clec4d*^*−/−*^ MDSCs were stimulated with or without *C. tropicalis* (MOI = 1) for 24 h. For glycolysis inhibition experiment, WT MDSCs were stimulated with *C. tropicalis* (MOI = 1) in the presence or absence of glycolysis inhibitor 2-DG (1 mM) for 24 h. In addition, MDSCs from WT and *Clec4d*^−/−^ mice bearing AOM/DSS-induced CAC gavaged with or without *C. tropicalis* were collected. Then, MDSCs were cocultured with CD8^+^ T cells (2 × 10^5^ cells/well) labeled with 5 µM carboxyfluorescein succinimidyl ester (CFSE; eBioscience, #C34570) at a 1:1 ratio in 96-well plates in the presence of 4 µg/mL anti-CD3 (eBioscience, #16-0031-81) and 2 µg/mL anti-CD28 mAbs (eBioscience, #16-0281-81) for 72 h. The CFSE-fluorescence intensity was analyzed using flow cytometry to determine the proliferation of CD8^+^ T cells.

#### ELISA

The concentrations of IFNγ in the supernatants was measured by Mouse IFNγ ELISA Kit according to the instructions of manufacturer.

### Bioinformatics analysis

The substrate phosphorylation sites of Syk, posttranslational modification (PTM) sites of PKM2 and phosphorylated tyrosine sites of PKM2 were obtained from PhosphoSitePlus® (http://www.phosphosite.org/) [32].

*Pkm*, *Glut1*, *Hk2*, *Ldha*, *Pdk1* and *Nos2* expression in human CRC tissues (T, n = 275) and normal colon tissues (N, n = 349) from TCGA and GTEx datasets for Colon adenocarcinoma (COAD), analyzed by the GEPIA2 online analysis tool (http://gepia2.cancer-pku.cn/#index). [33] Correlation between *Pkm* expression and *Glut1*, *Hk2*, *Ldha*, *Pdk1*, *Nos2*, *Ptgs2* (*Cox2*), *Cybb* (*Nox2*) expression in TCGA database for COAD analyzed by the GEPIA2.

### Metabolism assays

For unbiased metabolomics analysis, MDSCs were stimulated with heat-inactivated *C.tropicalis* (MOI = 1) for 24 h, and then metabolite profiling were identified by liquid chromatography-tandem mass spectrometry (LC-MS/MS) system by Shanghai Lu-Ming Biotech Co., Ltd., Shanghai, China. The acquired data were analyzed using the progenesis QI (Waters Corporation, Milford, USA) Data Processing Software.

For measurement of glucose consumption and lactic acid production, after MDSCs were treated, glucose and lactate levels in the cell culture supernatants were measured using a Glucose Assay Kit (Eton Bioscience, #1,200,031,002) and a Lactate Assay Kit (Eton Bioscience, #1,200,011,002) according to the manufacturer’s protocol.

The extracellular acidification rate (ECAR) of MDSCs were detected using Seahorse XF Glycolysis Stress Test Kit (Agilent Technologies). For real-time analysis of the ECAR, MDSCs were analyzed with Agilent Seahorse XFe96 Extracellular Flux Analyzer (Seahorse Bioscience) following manufacturer’s instructions.

### Gene silencing by siRNA transfection of MDSCs

siRNA specifically targeting mouse HK2 and PKM2 and negative control siRNAs (NC) were purchased from RiboBio (Guangzhou, China). The siRNA sequences are shown in Additional file 1: Table S4. MDSCs were plated in six-well and twelve-well plates for 24 h and then siRNA (50 nM) was transfected into MDSCs using Lipofectamine RNAi-MAX Transfection Reagent (Invitrogen, Carlsbad, CA, US) according to the manufacturer’s protocol.

### Histopathological and immunohistochemical (IHC) analysis

Hematoxylin and eosin (H&E) was used to stain slices of paraffin-embedded colon tumor tissues. Using clinical and pathological criteria, a pathologist who was unaware of the mice genotype and therapy evaluated the histological score of colon tumor samples [34].

For IHC staining, the colon tumor tissues sections were stained with the indicated antibodies. The antibodies used are listed in Additional file 1: Table S2. The percentages of positive cells were quantified using ImageJ (https://imagej.nih.gov/ij/).

### Immunofluorescence

The sections of paraffin-embedded colon tumor tissues were prepared. Then the sections were incubated with anti-CD11b (AiFang Biological, #AF300081), anti-PKM2 (Cell Signaling Technology, #4053), anti-phospho-PKM2 (Tyr105) (Bioss, #bs-3334R) and anti-iNOS (Sigma, #SAB4502012) primary antibodies. DAPI was used for nuclear counterstaining. The sections were detected using a confocal laser scanning microscope.

### Human samples

This study comprised 50 colorectal cancer patients who were treated at Yancheng First Hospital, an affiliate hospital of Nanjing University Medical School, from April 2016 to December 2018. When the patients had surgery, the nearby non-tumor tissues and colorectal cancer tissues were obtained. The Medical School of Nanjing University’s ethical committee has authorized every research involving human samples, and all individuals have given their written informed permission.

### Statistical analysis

The results were expressed as the mean ± SEM. Statistical analysis was performed with GraphPad Prism 8.0 (https://www.graphpad.com/scientific-software/prism/) software. Two-tailed Student’s *t*-test was used to compare the significant differences between two groups and one-way analysis of variance (ANOVA) was performed to compare the differences among multiple groups. *P* value < 0.05 was considered to be statistically significant. n.s., *P* > 0.05; *P < 0.05, **P < 0.01, ***P < 0.001.

## Results

### ***C. tropicalis*promotes the immunosuppressive function of MDSCs through Dectin-3**

**Fig. 1:**
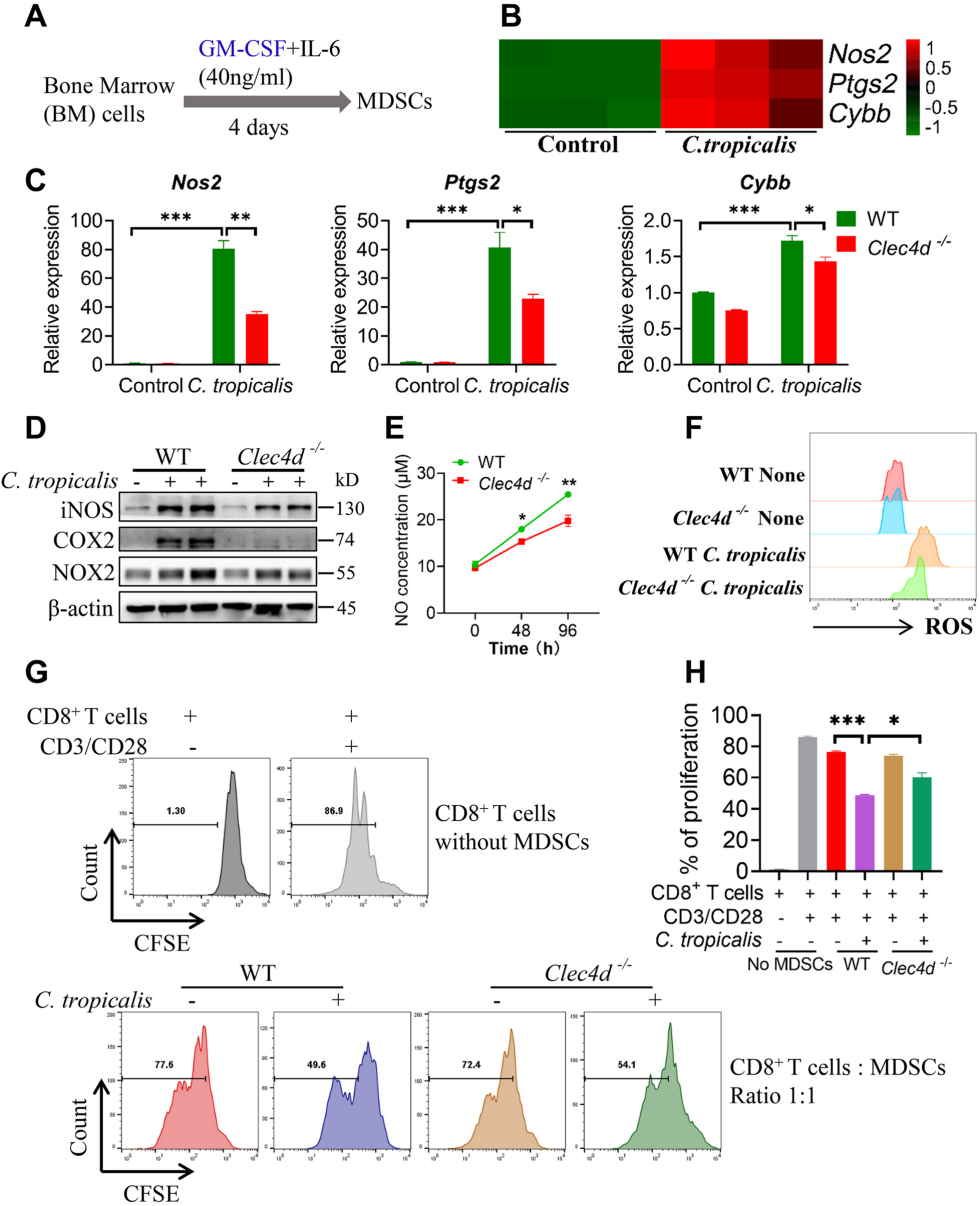
* C. tropicalis* promotes the immunosuppressive function of MDSCs through Dectin-3. **A** The scheme showing the process of inducing bone marrow cells to differentiate into MDSCs. **B** RNA-seq analysis showing the upregulated genes associated with the immunosuppressive function in MDSCs stimulated with heat-inactivated *C. tropicalis* (MOI = 2) for 6 h (n = 3). **C** WT and *Clec4d*^*−/−*^ MDSCs were stimulated with heat-inactivated *C. tropicalis* (MOI = 1) for 6 h. *Nos2*, *Ptgs2* and *Cybb* mRNA expression were measured by quantitative real-time PCR (q-PCR). **D** WT and *Clec4d*^*−/−*^ MDSCs were stimulated with heat-inactivated *C. tropicalis* (MOI = 1) for 48 h. Cell lysates were analyzed by immunoblotting for iNOS, COX2 and NOX2. **E** and **F** WT and *Clec4d*^*−/−*^ MDSCs were stimulated with heat-inactivated *C. tropicalis* (MOI = 5) for the indicated time or 48 h. NO concentration in culture supernatants was measured by NO assay kit (**E**). ROS production was analyzed by flow cytometry. Representative histograms of ROS production in WT and *Clec4d*^*−/−*^ MDSCs was shown (**F**). **G** and **H** The suppressive effect of MDSCs on the proliferation of CD8^+^ T cells was analyzed by flow cytometry. The results shown here are expressed as the mean ± SEM. Each panel is a representative experiment of at least three independent biological replicates. *p < 0.05, **p < 0.01, ***p < 0.001. The following statistical analyses were performed: unpaired Student’s *t*-test or one-way ANOVA where appropriate

To investigate the role of C-type lectin receptor, Dectin-3 on differentiation and immunosuppressive function of MDSCs, BM cells from wild type (WT) or *Clec4d*^*−/−*^ mice were cultured in vitro with murine IL-6 and GM-CSF to acquire MDSCs (Fig. 1A) [30]. Both WT and *Clec4d*^*−/−*^ MDSCs were differentiated properly with a purity of 90% after 4 days of culture. The proportion of MDSCs, G-MDSCs and M-MDSCs exhibited no difference between WT and *Clec4d*^*−/−*^ mice before and after culture (Additional file 1: Fig. S1A and B), suggesting Dectin-3 has no effect on MDSCs differentiation.

Next, we explored whether Dectin-3 mediated the immunosuppressive function of MDSCs modulated by *C. tropicalis*. Therefore, we first detected the expression of Dectin-3 in MDSCs stimulated with or without *C. tropicalis*. We found that *C. tropicalis* significantly up-regulated the expression of Dectin-3 (Additional file 1: Fig. S1C). Then we performed RNA-seq analysis in MDSCs stimulated with or without *C. tropicalis*. The expression of *Nos2* (iNOS), *Ptgs2* (COX2) and *Cybb* (NOX2), which are associated with the immunosuppressive function of MDSCs, were significantly upregulated upon *C. tropicalis* stimulation **(**Fig. 1B**)**, a finding that was supported by qPCR and western blot (Fig. 1C, D). However, their expression in MDSCs after stimulation with *C. tropicalis* was notably decreased by Dectin-3 deficiency (Fig. 1C, D). Consistently, *C. tropicalis* remarkably increased the production of NO and ROS, which are the products of iNOS and NOX2. And Dectin-3 deficiency resulted in a significant reduced NO and ROS production in MDSCs after *C. tropicalis* stimulation (Fig. 1E, F). After *C. tropicalis* stimulation, the immunosuppressive effects of MDSCs on the proliferation of CD8^+^ T cells were noticeably heightened. However, Dectin-3-deficient MDSCs had lower immunosuppressive effects than WT MDSCs following stimulation by *C. tropicalis* (Fig. 1G, H). We also found that under the condition of *C. tropicalis* stimulation, Dectin-3-deficient MDSCs had significantly lower suppressive ability on IFNγ secretion by CD8^+^ T cells than WT MDSCs (Additional file 1: Fig. S1D). Consistent with the above results, we also further demonstrated that *C. tropicalis* significantly enhanced the immunosuppressive function of MDSCs via Dectin-3 in mice bearing AOM/DSS-induced CAC (Additional file 1: Fig. S1E and F). Taken together, our data demonstrate that *C. tropicalis* promotes the immunosuppressive function of MDSCs through Dectin-3.

### **Dectin-3 mediates*C. tropicalis*-induced glycolysis activation in MDSCs**

**Fig. 2:**
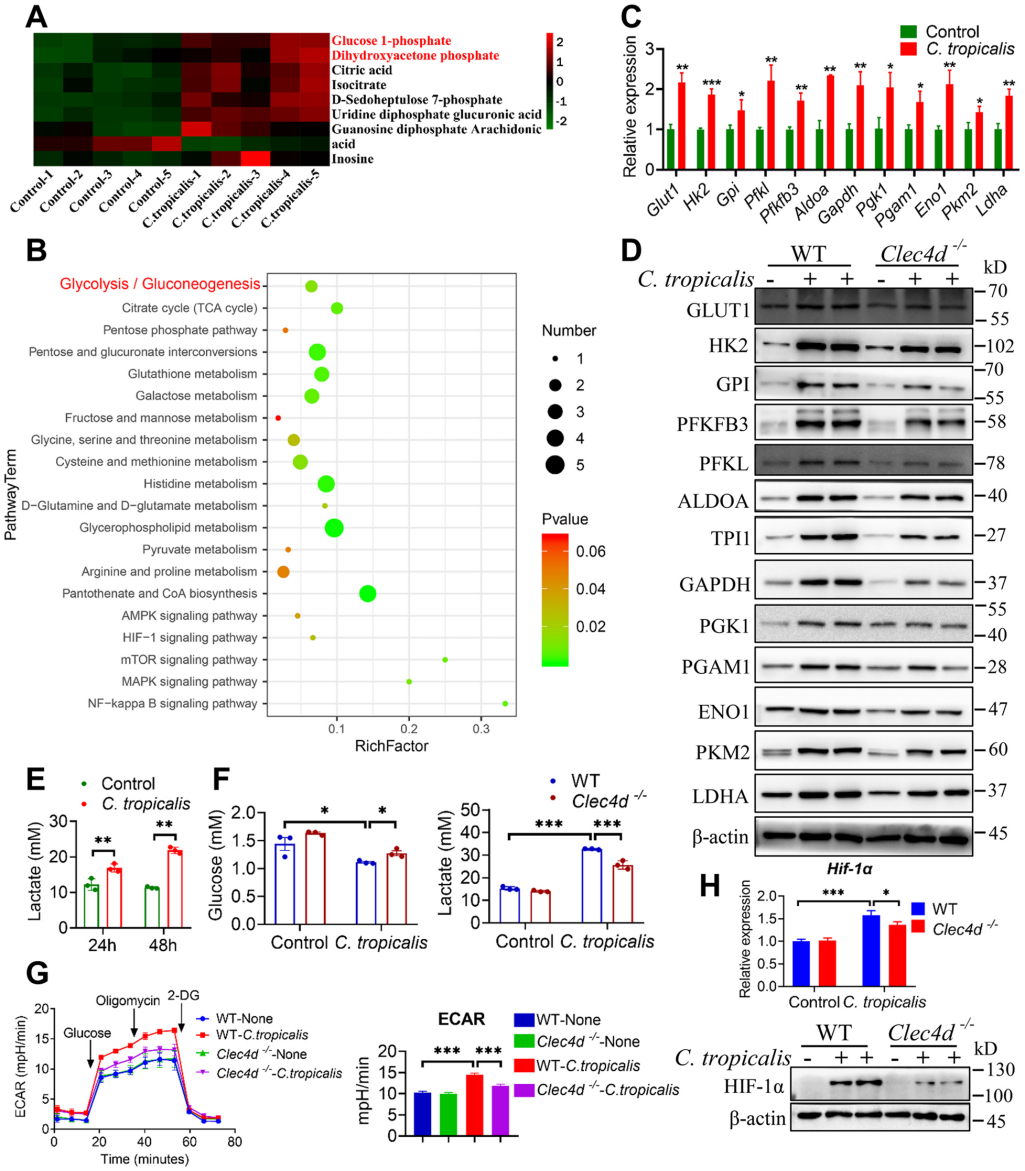
Dectin-3 mediates *C. tropicalis*-induced glycolysis activation in MDSCs. **A** Heatmaps showing the upregulation of metabolites of the glucose metabolism in MDSCs treated with *C. tropicalis* (MOI = 1) for 24 h (n = 5). **B** Metabolic pathway enrichment analysis shows the pathways with the significant change. **C** WT MDSCs were stimulated with heat-inactivated *C. tropicalis* (MOI = 1) for 24 h. The mRNA expression of glycolytic enzymes was measured by q-PCR. **D** WT and *Clec4d*^*−/−*^ MDSCs were stimulated with heat-inactivated *C. tropicalis* (MOI = 1) for 48 h. Cell lysates were analyzed by immunoblotting for glycolytic enzymes. **E** WT MDSCs were stimulated with heat-inactivated *C. tropicalis* (MOI = 5) for 24 and 48 h. Lactate secretion by MDSCs were determined by lactate assay kit. **F** WT and *Clec4d*^*−/−*^ MDSCs were stimulated with heat-inactivated *C.tropicalis* (MOI = 5) for 48 h. Lactate secretion and glucose consumption by MDSCs were determined by lactate and glucose assay kit. **G** WT and *Clec4d*^*−/−*^ MDSCs were stimulated with or without *C.tropicalis* (MOI = 2), then the ECAR level of these cells was measured by Agilent Seahorse XFe96 Analyzer. **H** WT and *Clec4d*^*−/−*^ MDSCs were stimulated with heat-inactivated *C. tropicalis* (MOI = 1) for 6 h. Hif-1α mRNA expression were measured by q-PCR. WT and *Clec4d*^*−/−*^ MDSCs were stimulated with heat-inactivated *C. tropicalis* (MOI = 2) for 42 h. Cell lysates were analyzed by immunoblotting for HIF-1α. The results shown here are expressed as the mean ± SEM. Each panel is a representative experiment of at least three independent biological replicates. *p < 0.05, **p < 0.01, ***p < 0.001. The following statistical analyses were performed: unpaired Student’s *t*-test or one-way ANOVA where appropriate

To elucidate the effect of *C. tropicalis* on the metabolic reprogramming of MDSCs, unbiased metabolomics profiling was performed in MDSCs stimulated with or without *C. tropicalis* for 24 h. Principal component analysis (PCA), (orthogonal) partial least-squares-discriminant analysis (OPLS-DA) and (PLS-DA) showed that the principal component of metabolites in MDSCs stimulated by *C. tropicalis* was obviously altered (Additional file 1: Fig. S2A). We found significant increases in 302 metabolites and decreases in 150 metabolites (p < 0.05) (Additional file 1: Fig. S2B and C). As shown in Fig. 2A, C. *tropicalis* induced accumulation of metabolic intermediates related to glucose metabolism including glucose 1-phosphate and dihydroxyacetone phosphate. Metabolic pathway enrichment analysis revealed that *C. tropicalis* stimulation induced changes in glycolysis metabolic pathways **(**Fig. 2B**)**. This suggests that *C. tropicalis* may induce glycolysis activation of MDSCs. To validate the above results, we first examined the expression of glycolytic related enzymes in MDSCs. The mRNA and protein expression of glycolytic related enzymes, including GLUT1, HK2, GPI, PFKL, PFKFB3, ALDOA, TPI1, GAPDH, PGK1, PGAM1, ENO1, PKM2 and LDHA were markedly up-regulated in MDSCs treated with *C. tropicalis* compared with untreated MDSCs (Fig. 2C, D). Moreover, *C. tropicalis* stimulation obviously enhanced glycolysis of MDSCs by increasing glucose uptake level, lactate production and extracellular acidification rate (ECAR) level **(**Fig. 2E-G**)**. Further experiments indicated that Dectin-3 deficiency resulted in reduced levels of glycolysis in MDSCs upon *C. tropicalis* stimulation **(**Fig. 2D, F and G). Hypoxia-inducible factor 1α (HIF-1α) has been demonstrated to be an essential regulator of glycolysis, promoting the expression and activity of glycolysis-related enzymes [35, 36]. Consistently, we discovered that *C. tropicalis* enhanced the mRNA and protein expression of HIF-1α in MDSCs via Dectin-3 **(**Fig. 2H**)**. Collectively, these data suggest that Dectin-3 mediates *C. tropicalis*-induced glycolytic reprogramming of MDSCs.

### **Glycolysis mediates*C. tropicalis*–enhanced immunosuppressive function of MDSCs**

**Fig. 3:**
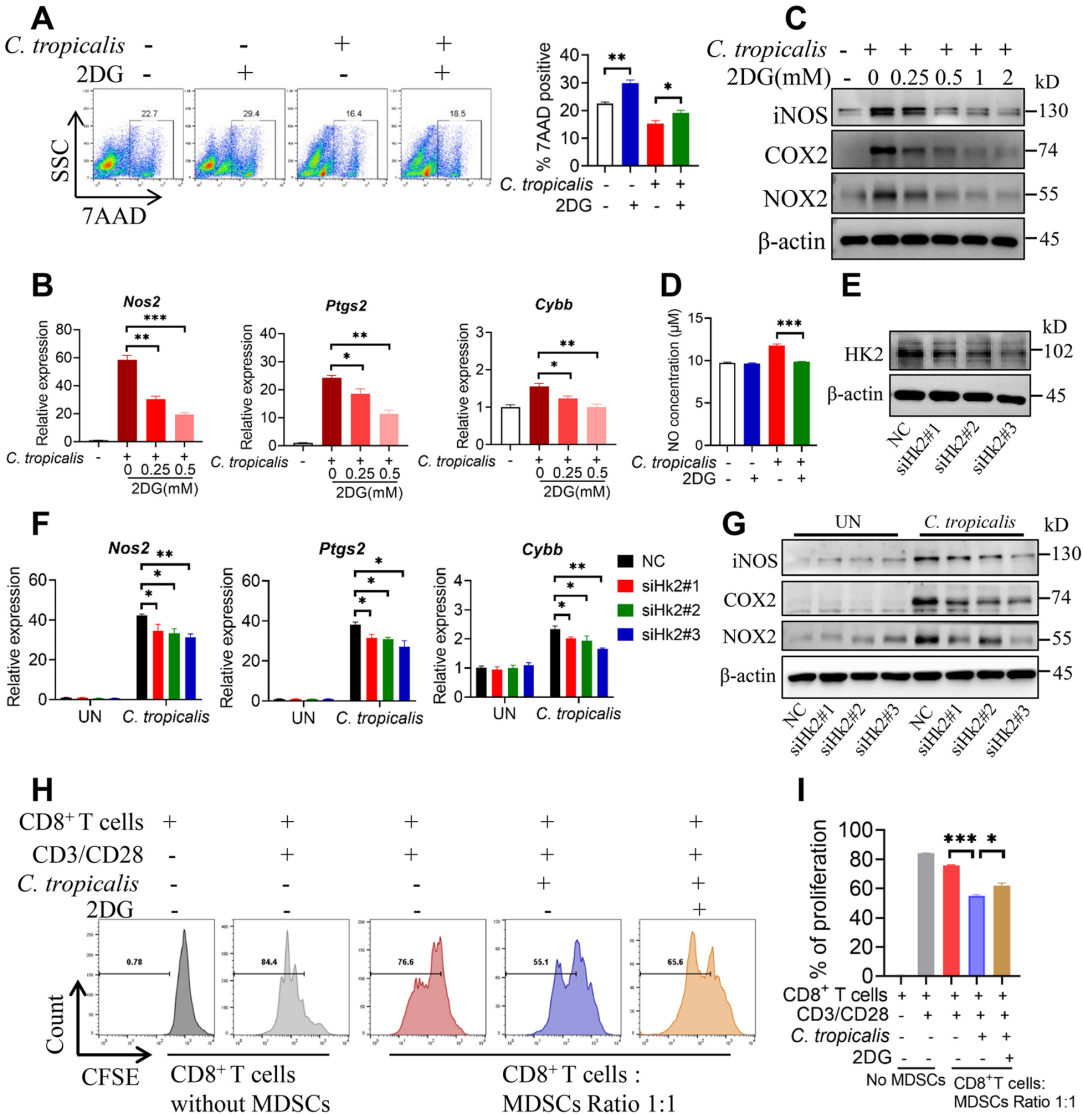
Glycolysis mediates *C. tropicalis*-enhanced immunosuppressive function of MDSCs.** A** WT MDSCs were stimulated with heat-inactivated *C. tropicalis* (MOI = 1) in combination with or without glycolysis inhibitor 2-DG (1 mM) for 24 h. 7AAD viability was determined by flow cytometry. **B** and **C** WT MDSCs were stimulated with heat-inactivated *C. tropicalis* (MOI = 1) in the presence or absence of glycolysis inhibitor 2-DG (as indicated concentration) for 12 or 18 h. iNOS, COX2, NOX2 mRNA expression were measured by q-PCR (**B**). Cell lysates were analyzed by immunoblotting for iNOS, COX2, NOX2 (**C**). **D** WT MDSCs were stimulated with heat-inactivated *C. tropicalis* (MOI = 5) in the presence or absence of glycolysis inhibitor 2-DG (1 mM) for 48 h. NO concentration in culture supernatants was measured by NO assay kit. **E**-**G** WT MDSCs were pretransfected with HK2 siRNA for 24 h prior to stimulation with heat-inactivated *C. tropicalis* (MOI = 2) for 12 or 24 h. *Nos2*, *Ptgs2* and *Cybb* mRNA expression were measured by qPCR (F). Cell lysates were analyzed by immunoblotting for HK2 (**E**), iNOS, COX2 and NOX2 (**G**). **H** and **I** The suppressive effect of MDSCs on the proliferation of CD8^+^ T cells was analyzed by flow cytometry. The results shown here are expressed as the mean ± SEM. Each panel is a representative experiment of at least three independent biological replicates. *p < 0.05, **p < 0.01, ***p < 0.001. The following statistical analyses were performed: unpaired Student’s *t*-test or one-way ANOVA where appropriate

We further explored whether glycolysis could modulate the immunosuppressive function of MDSCs enhanced by *C. tropicalis*. To address this issue, WT MDSCs were stimulated with *C. tropicalis* in the presence or absence of glycolysis inhibitor 2-Deoxy-D-glucose (2DG). 2DG treatment decreased the survival of MDSCs stimulated with *C. tropicalis* **(**Fig. 3A**)**. Moreover, treatment of MDSCs with 2DG markedly impaired the mRNA and protein expression of iNOS, COX2 and NOX2 up-regulated by *C. tropicalis* in a dose-dependent manner (Fig. 3B and C). Similarly, 2DG also attenuated NO production of MDSCs induced by *C. tropicalis* (Fig. 3D**)**. To further verify the effect of glycolysis on the immunosuppressive function of MDSCs, we knocked down the first key enzyme for glycolysis, HK2 expression via siRNA in MDSCs **(**Fig. 3E**)**. As shown in Fig. 3F and G, HK2 knockdown markedly abrogated the induction of iNOS, COX2 and NOX2 expression in *C. tropicalis*-treated MDSCs. Furthermore, while *C. tropicalis*-treated MDSCs markedly inhibited the proliferation of CD8^+^ T cells, such suppression could all be efficiently attenuated by the treatment of MDSCs with 2DG **(**Fig. 3H and I). Together, the above results show that glycolysis could mediate *C. tropicalis*–enhanced immunosuppression of MDSCs.

### **iNOS-derived NO is required for*C. tropicalis*–induced glycolysis activation in MDSCs**

**Fig. 4:**
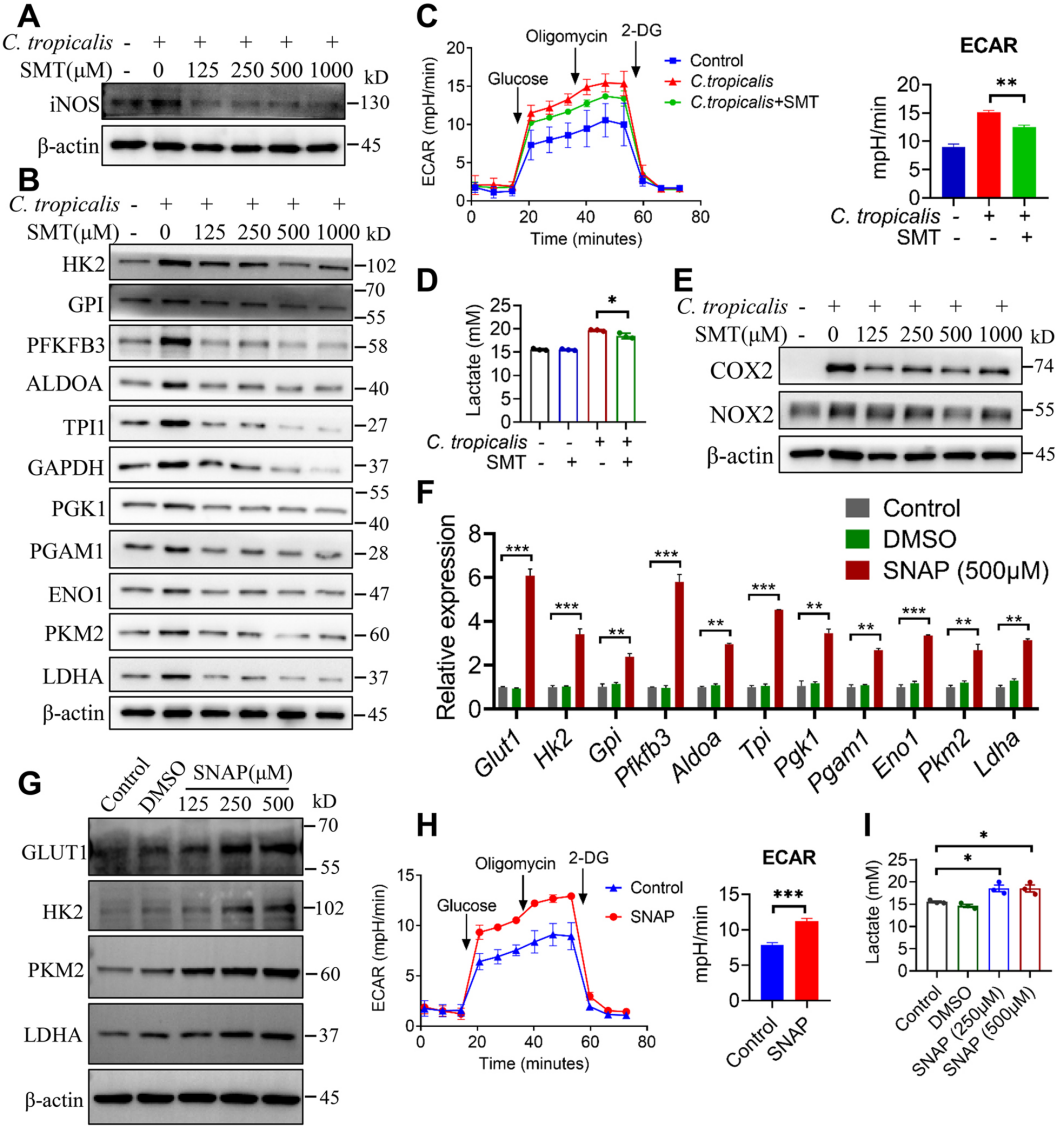
iNOS-derived NO is required for *C. tropicalis*-induced glycolysis activation in MDSCs. **A**, **B** and **E** WT MDSCs were stimulated with heat-inactivated *C. tropicalis* (MOI = 1) in combination with or without specific iNOS inhibitor SMT (as indicated concentration) for 18 h. Cell lysates were analyzed by immunoblotting for the indicated proteins. **C** WT MDSCs were stimulated with heat-inactivated *C. tropicalis* (MOI = 5) in combination with or without specific iNOS inhibitor SMT (500 µM) for 24 h. Lactate secretion by MDSCs were determined by lactate assay kit. **D** MDSCs were stimulated with *C. tropicalis* (MOI = 2) in the presence or absence of specific iNOS inhibitor SMT (500 µM), then the ECAR level of these cells was measured by Agilent Seahorse XFe96 Analyzer. **F** and **G** WT MDSCs were treated with NO donor (as indicated concentration) for 12 h. Glycolytic enzymes mRNA expression were measured by q-PCR (**F**). Cell lysates were analyzed by immunoblotting for the indicated proteins (**G**). **H** WT MDSCs were treated with NO donor (250 µM and 500 µM) for 24 h. Lactate secretion by MDSCs were determined by lactate assay kit. **I** WT MDSCs were treated with NO donor (500 µM), then the ECAR level of these cells was measured by Agilent Seahorse XFe96 Analyzer. The results shown here are expressed as the mean ± SEM. Each panel is a representative experiment of at least three independent biological replicates. *p < 0.05, **p < 0.01, ***p < 0.001. The following statistical analyses were performed: unpaired Student’s *t*-test or one-way ANOVA where appropriate

Next, we investigated the mechanism by which *C. tropicalis* induces glycolysis activation of MDSCs. Glycolytic reprogramming of LPS-activated DCs has been reported to be maintained by iNOS-derived NO [37–39]. Consistently, we also found *C. tropicalis* enhanced the production of iNOS-derived NO in MDSCs through Dectin-3. Thus, we assumed whether iNOS-derived NO is required for *C. tropicalis*–induced glycolytic reprogramming in MDSCs. To confirm this assumption, we treated *C. tropicalis*-stimulated MDSCs with SMT, an iNOS (NOS2)-specific inhibitor. The results showed that *C. tropicalis*–induced expression of iNOS and glycolytic enzymes were markedly blocked in SMT-treated MDSCs **(**Fig. 4A and B). Furthermore, SMT treatment significantly reduced the lactate production and the ECAR level in MDSCs upon *C. tropicalis* stimulation **(**Fig. 4C and D). This indicated that inhibition of iNOS could inhibit glycolytic reprogramming induced by *C. tropicalis*. Consistently, inhibition of iNOS abrogated the induction of COX2 and NOX2 expression in *C. tropicalis*-stimulated MDSCs **(**Fig. 4E**)**. In order to directly study the effect of nitric oxide on glycolysis of MDSCs, WT MDSCs were treated with the NO donor SNAP. As shown in Fig. 4F−I, SNAP obviously enhanced the mRNA and protein expression of glycolytic enzymes, lactate production and ECAR level. Taken together, these data unequivocally indicate that iNOS-derived NO is required for *C. tropicalis*–induced glycolytic reprogramming in MDSCs.

### ***C. tropicalis*induced Syk-mediated PKM2 Tyr105 phosphorylation and PKM2 nuclear translocation in MDSCs**

**Fig. 5:**
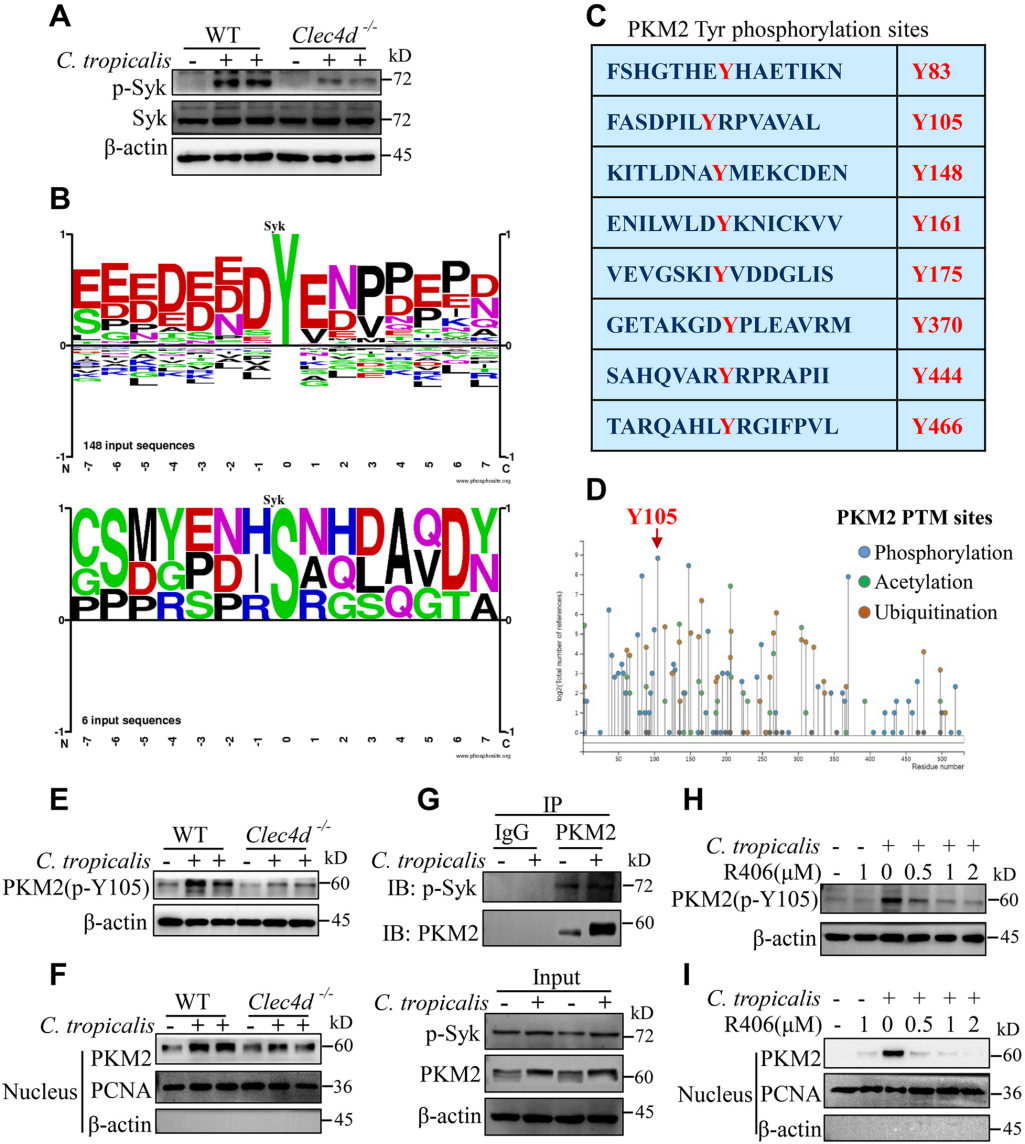
*C. tropicalis *induced Syk-mediated PKM2 Tyr105 phosphorylation and PKM2 nuclear translocation in MDSCs. **A** WT and *Clec4d*^*−/−*^ MDSCs were stimulated with heat-inactivated *C.tropicalis* (MOI = 2) for 24 h. Cell lysates were analyzed by immunoblotting for phosphorylated (p-) and total Syk. **B** Schematic representation showing the substrate phosphorylation sites of Syk. **C** List showing eight putative phosphorylated tyrosine sites of PKM2. **D** Schematic representation showing posttranslational modification (PTM) sites of PKM2. Data of B-D was from PhosphoSitePlus® (http://www.phosphosite.org/) [32]. **E** and **F** WT and *Clec4d*^*−/−*^ MDSCs were stimulated with heat-inactivated *C.tropicalis* (MOI = 2) for 6 or 24 h. Cell lysates were analyzed by immunoblotting for PKM2 (p-Y105) (**E**). Nuclear extracts were analyzed by immunoblotting for PKM2 (**F**). **G** WT MDSCs were stimulated with heat-inactivated *C.tropicalis* (MOI = 2) for 18 h. Immunoprecipitation was carried out with antibodies against PKM2 or rabbit IgG. The immunoprecipitates and cell lysates were analyzed by immunoblotting for the indicated proteins. **H** and **I** WT MDSCs were stimulated with heat-inactivated *C.tropicalis* (MOI = 2) in the presence or absence of Syk inhibitor R406 (as indicated concentration) for 18 or 24 h. Cell lysates were analyzed by immunoblotting for PKM2 (p-Y105) (**H**). Nuclear extracts were analyzed by immunoblotting for PKM2 (**I**). The results shown here are expressed as the mean ± SEM. Each panel is a representative experiment of at least three independent biological replicates. *p < 0.05, **p < 0.01, ***p < 0.001. The following statistical analyses were performed: unpaired Student’s *t*-test or one-way ANOVA where appropriate

C-type lectin receptor, Dectin-3 has been reported to couple with the kinase Syk, resulting in phosphorylation and activation of Syk during fungal infection [18, 40, 41]. As expected, we found that *C. tropicalis* significantly induced Dectin-3-mediated phosphorylation of Syk in MDSCs **(**Fig. 5A**)**. Increasing evidence suggests that PKM2 is translocated to the nucleus upon phosphorylation, where it promotes aerobic glycolysis [42, 43]. The ability of Syk, a non-receptor tyrosine kinase, to phosphorylate the substrate protein is well documented. Bioinformatics tools (PhosphoSitePlus®) analysis suggested Syk could phosphorylate serine and tyrosine residues of substrate proteins, and the primary function of Syk is to phosphorylate tyrosine residues **(**Fig. 5B**)**. To determine whether Syk phosphorylates tyrosine residues of PKM2, we predicted eight tyrosine phosphorylation sites of PKM2 using bioinformatics tools (PhosphoSitePlus®) **(**Fig. 5C**)**. We further found that PKM2 Tyr105 phosphorylation occurred most frequently **(**Fig. 5D**)**. So, we speculated Syk was most likely to phosphorylate PKM2 Tyr105. To test the above speculation, WT and Dectin-3-deficient MDSCs were stimulated with *C. tropicalis*. We observed that *C. tropicalis* significantly induced phosphorylation of PKM2 Tyr105 and nuclear translocation of PKM2 through Dectin-3 **(**Fig. 5E and F). To further confirm the relationship between Syk and PKM2, we performed co-immunoprecipitation assays and indicated that *C. tropicalis* promoted p-Syk binding to PKM2 **(**Fig. 5G**)**. In order to directly study the effect of Syk on PKM2 phosphorylation and translocation, *C. tropicalis*-stimulated MDSCs were treated with R406, an inhibitor of Syk. We found that treatment of MDSCs with R406 markedly impaired *C. tropicalis*-induced of PKM2 Tyr105 phosphorylation and PKM2 nuclear translocation **(**Fig. 5H and I). In addition, R406 also inhibited the expression of glycolytic enzymes, HIF-1α and the ECAR level in MDSCs after *C. tropicalis* stimulation (Additional file 1: Fig. S3A-C). Meanwhile, the protein expression of iNOS, COX2 and NOX2 were also down-regulated in *C. tropicalis*-stimulated MDSCs after R406 treatment (Additional file 1: Fig. S3D). Taken together, these data demonstrate that Syk is required for PKM2 Tyr105 phosphorylation and PKM2 nuclear translocation in *C. tropicalis*-treated MDSCs.

### **PKM2 nuclear translocation promotes HIF-1α-dependent glycolytic metabolism in MDSCs after*C. tropicalis*stimulation**

**Fig. 6:**
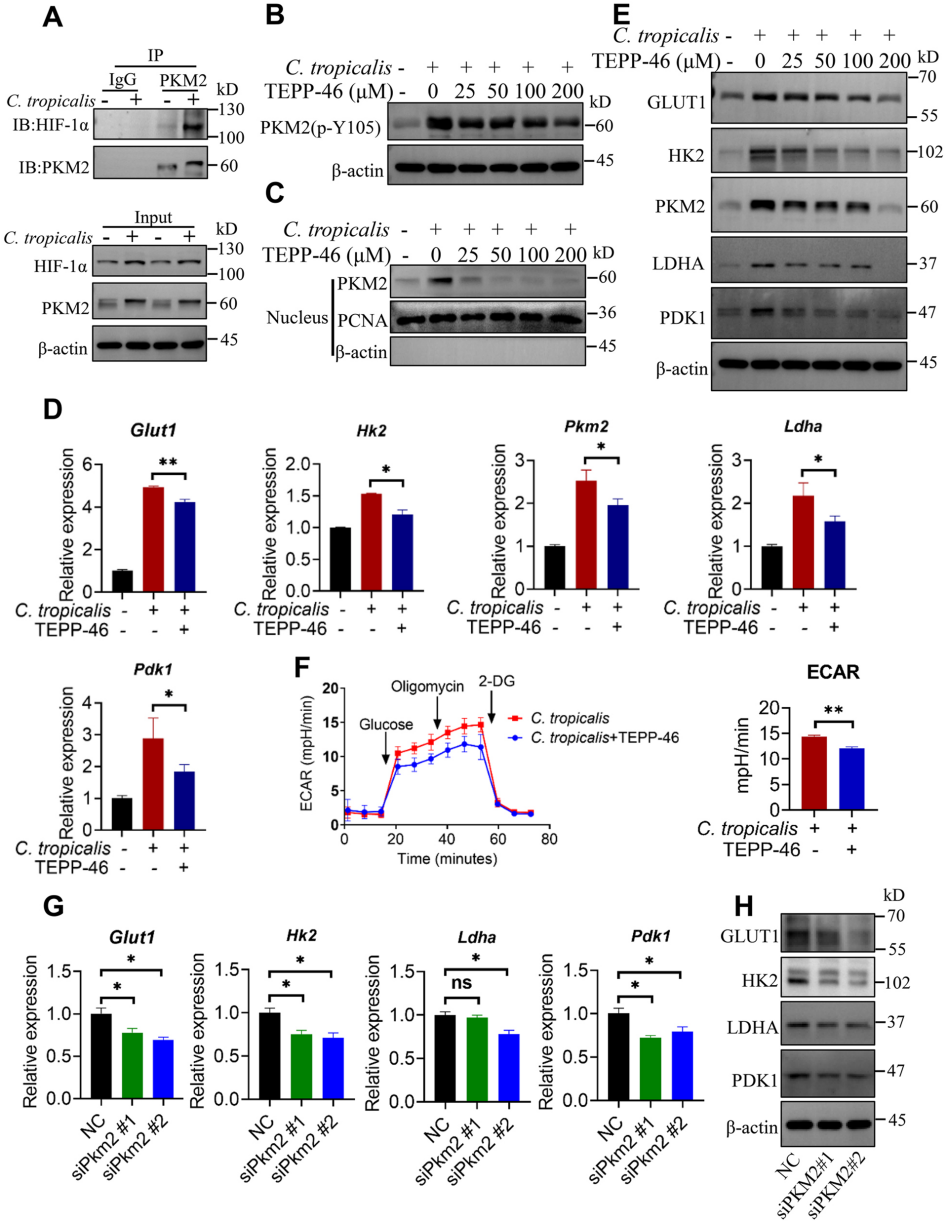
PKM2 nuclear translocation promotes HIF-1α-dependent glycolytic metabolism in MDSCs after *C. tropicalis* stimulation. **A** WT MDSCs were stimulated with heat-inactivated *C. tropicalis* (MOI = 2) for 24 h. Immunoprecipitation was carried out with antibodies against PKM2 or rabbit IgG. The immunoprecipitates and cell lysates were analyzed by immunoblotting for the indicated proteins. **B**, **C** and **E** WT MDSCs were stimulated with heat-inactivated *C. tropicalis* (MOI = 2) in the presence or absence of TEPP-46 (as indicated concentration) for 6 or 24 h. Cell lysates were analyzed by immunoblotting for the indicated proteins (**B** and **E**). Nuclear extracts were analyzed by immunoblotting for PKM2 (**C**). **D** WT MDSCs were stimulated with heat-inactivated *C. tropicalis* (MOI = 2) in the presence or absence of TEPP-46 (100 µM) for 6 h. Glycolytic enzymes mRNA expression were measured by q-PCR. **F** WT MDSCs were stimulated with *C. tropicalis* (MOI = 2) in the presence or absence of TEPP-46 (100 µM), then the ECAR level of these cells was measured by Agilent Seahorse XFe96 Analyzer. **G** and **H** WT MDSCs were pretransfected with PKM2 siRNA for 24 h prior to stimulation with heat-inactivated *C. tropicalis* (MOI = 2) for 6 or 18 h. Glycolytic enzymes mRNA expression were measured by q-PCR (**G**). Cell lysates were analyzed by immunoblotting for glycolytic enzymes (**H**). The results shown here are expressed as the mean ± SEM. Each panel is a representative experiment of at least three independent biological replicates. *p < 0.05, **p < 0.01, ***p < 0.001. The following statistical analyses were performed: unpaired Student’s *t*-test or one-way ANOVA where appropriate

In the present study, we revealed that the expression of HIF-1α was elevated in *C. tropicalis*-stimulated MDSCs (Fig. 2H). Therefore, we hypothesized whether *C. tropicalis* could induce the interaction between PKM2 and HIF-1α in MDSCs. The result indicated that treatment of MDSCs with *C. tropicalis* increased the interaction between PKM2 and HIF-1α (Fig. 6A). Emerging evidence have demonstrated that TEPP-46, a PKM2 activator promotes PKM2 tetramerization, which suppresses phosphorylation and nuclear translocation of PKM2 [44–46]. To further determine the effect of PKM2 nuclear translocation on glycolytic metabolism in *C. tropicalis*-stimulated MDSCs, MDSCs were treated with *C. tropicalis* in combination with or without TEPP-46. As expected, TEPP-46 significantly inhibited *C. tropicalis*–induced PKM2 Tyr105 phosphorylation and PKM2 nuclear translocation in a dose-dependent manner (Fig. 6B and C). This indicates that PKM2 phosphorylation at Tyr105 is necessary for nuclear translocation of PKM2. As shown in Fig. 6D and E, TEPP-46 markedly suppressed the mRNA and protein expression of HIF-1α-dependent glycolytic enzymes GLUT1, HK2, PKM2, LDHA and PDK1 in MDSCs after *C. tropicalis* stimulation. Moreover, TEPP-46 also resulted in the reduction of ECAR level in *C. tropicalis*-treated MDSCs (Fig. 6F). Inhibition of PKM2 nuclear translocation also resulted in a significant decreased expression of iNOS, COX2 and NOX2 in MDSCs after *C. tropicalis* stimulation (Additional file 1: Fig. S4A and B). To further verify the above results, we knocked down the expression of PKM2 in BM-MDSCs utilizing PKM2-targeted siRNA (Additional file 1: Fig. S4C). As shown in Fig. 6G and H, siPKM2 obviously blocked the mRNA and protein expression of HIF-1α-dependent glycolytic enzymes in *C. tropicalis*-treated MDSCs. Meanwhile, PKM2 knockdown also diminished the mRNA and protein expression of iNOS, COX2 and NOX2 in MDSCs after stimulation with *C. tropicalis* (Additional file 1: Fig. S4D and E). Together, these observations support the concept that PKM2 Tyr105 phosphorylation and PKM2 nuclear translocation are essential for *C. tropicalis*-induced HIF-1α-dependent glycolytic metabolism in MDSCs.

### **Pharmacological inhibition of PKM2 nuclear translocation alleviates*C. tropicalis*-aggravated CAC**

**Fig. 7:**
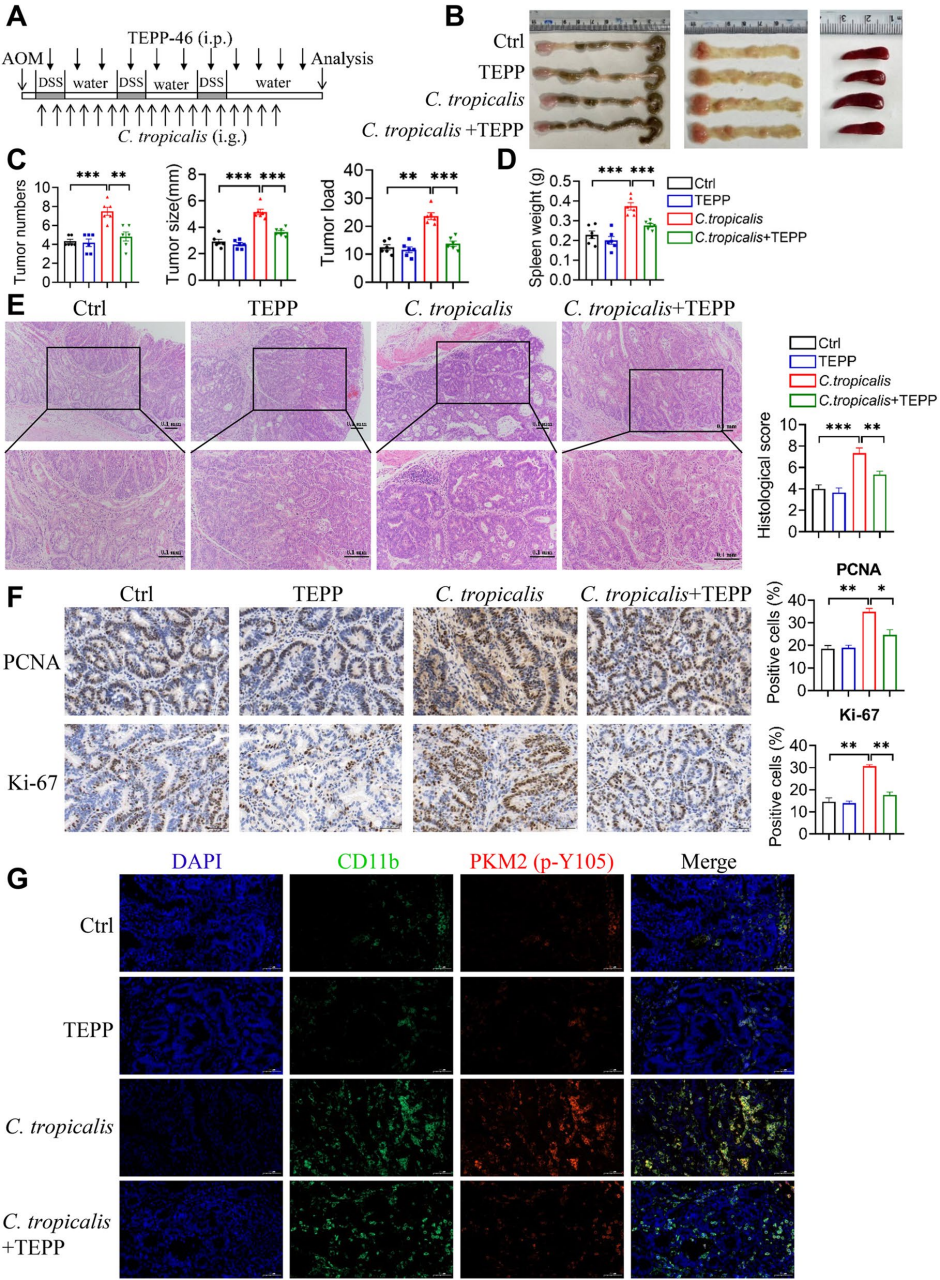
TEPP-46 ameliorates *C. tropicalis*-aggravated CAC. **A** Schematic showing the experimental design, timeline of AOM/DSS-induced CAC model (n = 6 for each group). **B** The representative pictures of colon tumors, the inside of the colon and spleens were displayed. **C** Tumor number, size, and load in each mouse were detected. **D** The spleen weight of each mouse was measured. **E** Representative histological images of colon tumors by H&E staining. Histological score was evaluated by a pathologist. Scale bars, 0.1 mm. **F** The expression of Ki-67 and PCNA in colon tumors tissue was detected by immunohistochemistry (IHC). The percentages of Ki-67-positive and PCNA-positive tumor cells were quantified. Scale bars, 50 μm. **G** The infiltration of CD11b^+^ myeloid cells and the level of PKM2 (p-Y105) of CD11b^+^ myeloid cells in colon tumors tissue were determined by immunofluorescence assay. Scale bars, 50 μm. The results shown here are expressed as the mean ± SEM. Each panel is a representative experiment of 6 independent biological replicates. *p < 0.05, **p < 0.01, ***p < 0.001. The following statistical analyses were performed: unpaired Student’s *t*-test or one-way ANOVA where appropriate

Considering that inhibition of PKM2 nuclear translocation can suppress *C. tropicalis*-induced glycolysis and immunosuppressive function in MDSCs, we tested the therapeutic possibility of TEPP-46 in the CAC model. Therefore, an AOM/DSS-induced CAC mouse model was created by injecting of one dose of AOM intraperitoneally, followed by 3 cycles of 2% DSS administration in water. And mice were mono-colonized with *C. tropicalis* and given TEPP-46 (TEPP) via intraperitoneal injection during the induction of tumorigenesis (Fig. 7A). We found that colonization of *C. tropicalis* significantly increased the tumor number, size, load and spleen weight of CAC mice (Fig. 7B–D). However, TEPP-46 treatment observably alleviated *C. tropicalis*-promoted colorectal tumorigenesis (Fig. 7B–D). Histopathological examination indicated that the histological score of colon tumor tissues was markedly higher in the *C. tropicalis*-gavaged tumor-bearing mice than in control mice (Fig. 7E). TEPP-46 treatment reduced the *C. tropicalis*-enhanced histological score of colon tumor tissues (Fig. 7E). To evaluate cellular proliferation in colon tumors, an immunohistochemical experiment was carried out. We found that blockade of PKM2 nuclear translocation substantially reduced the expression of proliferating cell nuclear antigen (PCNA) and Ki-67 in *C. tropicalis*-colonized mice with CAC (Fig. 7F). Immunofluorescence assay further confirmed that the level of PKM2 (p-Y105) in CRC-infiltrating CD11b^+^ myeloid cells was significantly increased in colon tumor tissues of *C. tropicalis*-colonized mice with CAC, which could be suppressed by TEPP-46 treatment (Fig. 7G). The proportion of MDSCs in colonic lamina propria (LP), bone marrow (BM) and spleen, as well as the proportion of CD4^+^ and CD8^+^ T cells in spleen was also measured. The results indicated that the proportion of MDSCs was remarkably increased in colonic LP, BM and spleen of *C. tropicalis*-colonized mice with CAC. Consistently, the proportion of CD4^+^ and CD8^+^ T cells was decreased in spleen after colonization of *C. tropicalis* (Additional file 1: Fig. S5A). TEPP-46 treatment markedly reversed the effect of *C. tropicalis* (Additional file 1: Fig. S5A). Furthermore, TEPP-46 reversed the immunosuppressive function of MDSCs enhanced by *C. tropicalis* in colonic LP (Additional file 1: Fig. S5B). We also found that *C. tropicalis* significantly enhanced the expression of enzymes associated with glycolysis, GLUT1, HK2, PKM2 and LDHA in colon tumor tissues, which was attenuated by TEPP-46 treatment (Additional file 1: Fig. S5C and D). Furthermore, TEPP-46 treatment substantially inhibited the expression of iNOS, COX2 and NOX2 in colon tumor tissues of *C. tropicalis*-colonized mice with CAC (Additional file 1: Fig. S5E and F). Overall, we conclude that blockade of PKM2 nuclear translocation ameliorates *C. tropicalis*- aggravated colon tumorigenesis.

### The high expression of PKM2, PKM2 (p-Y105) and iNOS in MDSCs infiltrated by CRC is associated with the development of human CRC

**Fig. 8:**
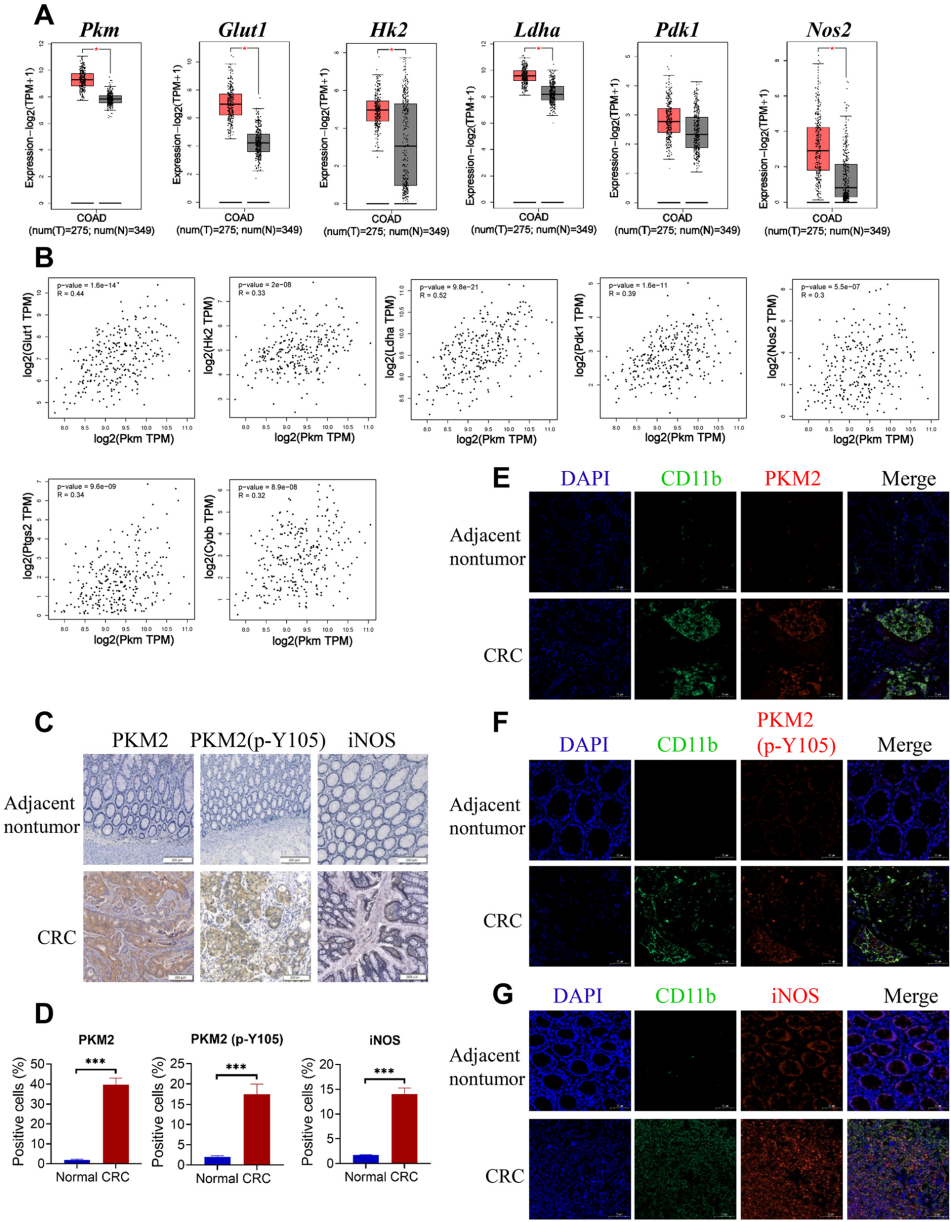
The infiltration of MDSCs is positively correlated with the level of PKM2, PKM2 (p-Y105) and iNOS in patients with CRC. **A** The indicated gene expression in human CRC tissues (T, n = 275) and normal colon tissues (N, n = 349) from TCGA and GTEx datasets for COAD, analyzed by the GEPIA2 online analysis tool. **B** Correlation between *Pkm* expression and *Glut1*, *Hk2*, *Ldha*, *Pdk1*, *Nos2*, *Ptgs2*, *Cybb* expression in TCGA database for COAD analyzed by the GEPIA2. **C** Representative IHC images of PKM2, PKM2(p-Y105) and iNOS levels in human CRC tissues (n = 50) and adjacent nontumor tissues (n = 50). The scale bar represents 200 μm. **D** The percentages of PKM2-positive, PKM2(p-Y105)-positive and iNOS-positive cells were quantified. **E**-**G** The infiltration of CD11b^+^ myeloid cells and PKM2, PKM2(p-Y105) and iNOS levels of CD11b^+^ myeloid cells in human CRC tissues were determined by immunofluorescence assay. Scale bars, 50 μm. The results shown here are expressed as the mean ± SEM. Each panel is a representative experiment of at least three independent biological replicates. *p < 0.05, **p < 0.01, ***p < 0.001. The following statistical analyses were performed: unpaired Student’s *t*-test

To clarify the relationship between PKM2, glycolysis, iNOS and the progression of human CRC, we first analyzed TCGA and GTEx databases for Colon adenocarcinoma (COAD) with GEPIA online tool [33]. The expression of *Pkm*, *Glut1*, *Hk2*, *Ldha* and *Nos2* was highly significant elevated in COAD tumors tissues compared to normal tissues (Fig. 8A). In addition, *Pkm* mRNA expression was positively correlated with the mRNA expression of *Glut1*, *Hk2*, *Ldha*, *Pdk1*, *Nos2*, *Ptgs2* and *Cybb* in COAD (Fig. 8B). Immunohistochemical (IHC) experiment revealed that human CRC tissues had considerably higher expression levels of PKM2, PKM2 Tyr105 phosphorylation, and iNOS than the surrounding nontumor tissues. (Fig. 8C and D). CD11b^+^ myeloid cells in human tumors have been reported to contain a large number of MDSCs [47]. We also further found that CD11b^+^ myeloid cells of CRC tissues showed elevated expression of PKM2, PKM2 (p-Y105) and iNOS. (Fig. 8E-G). To directly verify the expression of PKM2, PKM2 (p-Y105) and iNOS in MDSCs infiltrated by CRC, we further detected the expression of PKM2, PKM2 (p-Y105) and iNOS in MDSCs (HLA-DR^−/low^ CD33^+^ CD14^−/low^ CD66b^+^) from human CRC tissues and adjacent nontumor tissues by flow cytometry (Additional file 1: Fig. S6A). We found that MDSCs of CRC tissues showed elevated expression of PKM2, PKM2 (p-Y105) and iNOS (Additional file 1: Fig. S6B). In summary, all the clinical data demonstrate that infiltration of MDSCs is positively associated with the level of PKM2, PKM2 (p-Y105) and iNOS in patients with CRC.

## Discussion

Through the Dectin-1/ROS axis, the pathogenic fungi *C. albicans* and *A. fumigatus* activate MDSCs that can dampen T cell responses [48]. Consistent with our earlier research, *C. tropicalis*, a pathogenic fungus, promotes MDSC accumulation in colon tumors and activates their immunosuppressive activity, which aggravates colon cancer growth [10]. Moreover, this study further suggested that compared to healthy persons, CRC patients had a greater abundance of *C. tropicalis*. The most recent study this year also showed that *C. tropicalis* was significantly enriched in Crohn’s disease patients and mice with *C. tropicalis* infection had increased vulnerability to colitis [49]. However, the specific molecular mechanism by which *C. tropicalis* promotes CRC remains undetermined. A potentially effective treatment approach for colon cancer may involve targeting MDSCs. Mechanistically, our present study provides evidence that *C. tropicalis* promotes immunosuppressive function of MDSCs through the pattern recognition receptor Dectin-3 and Syk. Notably, *C. tropicalis* enhanced the expression of iNOS, COX2 and NOX2, production of nitric oxide (NO) and reactive oxygen species (ROS) in MDSCs, which can suppress T cell immune responses. Our most recent study indicated that *C. albicans* increased innate lymphoid cell release of IL-22, which encouraged CRC [50]. Therefore, we can conclude clearly that the effect of *C. albicans* on CRC development is different from that of *C. tropicalis*. This work is the first to connect the immunosuppressive properties of MDSCs with Dectin-3 signaling pathway. Further research is needed to determine whether Dectin-2 is involved in the immunosuppressive function of MDSCs.

A growing body of studies have demonstrated that aerobic glycolysis is essential for the expansion and function of MDSCs in tumor [28, 29, 51, 52]. To maintain immunosuppressive activities, the glycolysis level of MDSC in tumors is elevated. Unexpectedly, emerging evidence suggests that cancer cells preferentially utilize glutamine, whereas myeloid cells, particularly MDSCs, are better able to absorb intratumoral glucose in the tumour microenvironment (TME) [27]. Nevertheless, whether and how aerobic glycolysis modulates immunosuppressive function of MDSCs induced by *C. tropicalis*, subsequently, affects tumor immunity, are poorly understood. In this work, our metabolomic analysis reveal that *C. tropicalis* significantly enhances the glycolysis of MDSCs. And upregulation of glycolysis induced by *C. tropicalis* in MDSCs supports the survival and immunosuppressive function of MDSCs, which may represent a promising mechanism by which *C. tropicalis* can mediate the function of MDSCs. In addition, our metabolomic analysis also show the pathways with the most significant change in Glycerophospholipid metabolism and CoA biosynthesis pathway. This further supports the finding that Dectin-3 has the greatest impact on COX2, which contributes to lipid-related PGE2 metabolism of MDSCs [53–55]. We do not exclude that glycerophospholipid metabolism and CoA biosynthesis pathways may also play important roles in the expression of iNOS, COX2 and NOX2 and the immunosuppressive function of MDSCs induced by *C. tropicalis*. This will also be the focus of our future research. Therefore, our present research has established a possible causal relationship between glycolytic reprogramming and *C. tropicalis*-promoted immunosuppressive function of MDSCs. However, the intricate molecular mechanisms through which *C. tropicalis* induces glycolytic reprogramming of MDSCs remain elusive.

Our current results suggest that *C. tropicalis* increases the production iNOS-derived NO, which in turn enhances glycolysis level of MDSCs in a positive feedback manner. Nevertheless, the molecular mechanisms by which nitric oxide promotes glycolysis of MDSCs have not been thoroughly investigated in this study. We hypothesize that NO might specifically target pyruvate dehydrogenase (PDH), which blocks pyruvate from entering the TCA cycle [56]. Thus, this leads MDSCs to rely on glycolysis for ATP generation. This hypothesis remains to be further investigated, which is also the focus of our next research.

PKM2 operates as a rate-limiting enzyme in glycolysis, but more crucially, as a coactivator of HIF-1α. In malignancies, phosphorylation of PKM2 enhances the Warburg effect [42, 43, 57]. Moreover, phosphorylation of PKM2 leads to nuclear translocation of PKM2 and PKM2 works with HIF-1α to coactivate the expression of the target gene for HIF-1α [36, 45, 58]. In this study, we first found that Syk phosphorylates PKM2 Tyr105 in MDSCs after stimulation with *C. tropicalis*, which leads to PKM2 nuclear translocation. Secondly, the interaction between nuclear PKM2 and HIF-1α is enhanced, which promotes HIF-1α-dependent glycolytic metabolism. Finally, pharmacological inhibition of PKM2 nuclear translocation attenuates *C. tropicalis*-aggravated CAC. Furthermore, TEPP-46 treatment alone does not induce a reduction in tumor progression compared to the control. We speculated that in our current study, MDSCs only maintained lower levels of glycolysis and PKM2 activation in the absence of *C. tropicalis*. The lower levels of glycolysis and PKM2 activation in MDSCs had little effect. Therefore, TEPP treatment alone had little effect on glycolysis and PKM2 activation of MDSCs in the absence of *C. tropicalis*. But we did not explore the molecular mechanism by which phosphorylated PKM2 translocates to the nuclear. We hypothesized that phosphorylation of PKM2 by Syk results in the formation of a dimer of PKM2 from a tetramer [59]. In turn, dimeric PKM2 translocates to the nuclear to promote the activity of HIF-1α [45]. It is necessary to be further investigated this hypothesis more thoroughly. To date, no study has reported that pharmacological inhibition of PKM2 nuclear translocation could treat CRC patients. This treatment approach is still in preclinical research. Therefore, whether this treatment approach is feasible in CRC patients remains to be investigated. In our present study, both in vitro and in vivo experiments demonstrated that TEPP-46 ameliorated *C. tropicalis*-aggravated CRC by pharmacologically inhibiting PKM2 nuclear translocation. Whether TEPP-46 directly targets *C. tropicalis* remains unclear. We suppose that this is possible, but it needs to be further verified experimentally. This will also be the focus of our future research. Thus, pharmacological inhibition of PKM2 nuclear translocation may represent a promising therapeutic strategy for the treatment of CRC.

## Conclusion

**Fig. 9:**
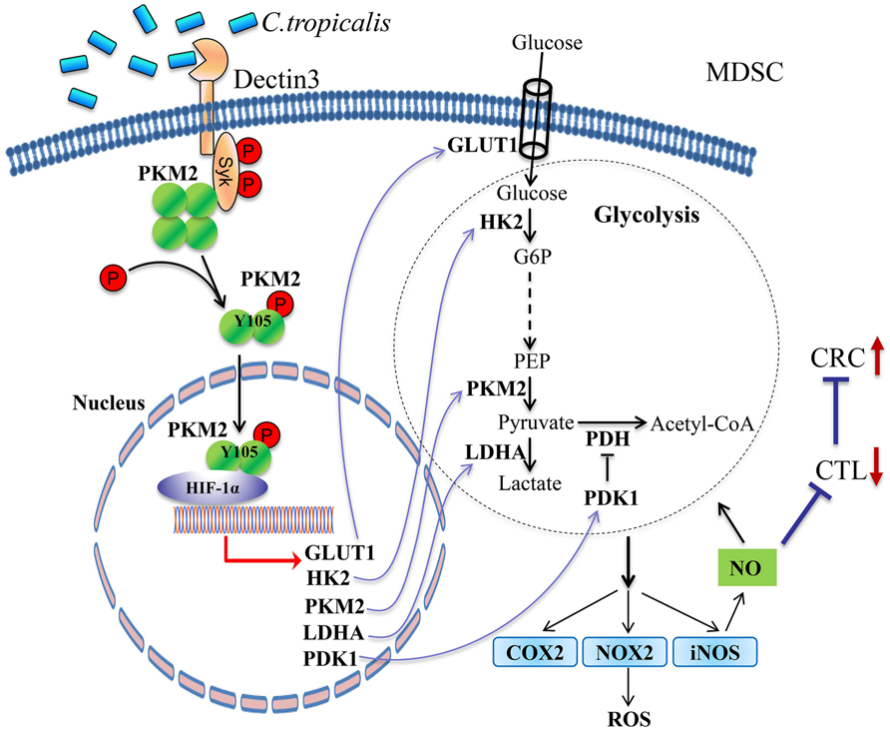
A proposed model. *C. tropicalis* activates Syk through Dectin-3 receptor in MDSCs. Then activated p-Syk phosphorylates PKM2 Tyr 105, which results in nuclear translocation of PKM2. Subsequently, nuclear PKM2 interacts with HIF-1α and enhances the expression of HIF-1α target genes encoding glycolytic enzymes, GLUT1, HK2, PKM2, LDHA and PDK1, which in turn promotes aerobic glycolysis, immunosuppression of MDSCs and colorectal tumorigenesis

Our studies identify for the first time that *C. tropicalis* enhances the interaction between Syk and PKM2 in MDSCs, which results in phosphorylation of PKM2 at Tyr105. PKM2 Tyr105 phosphorylation is essential for PKM2 nuclear translocation. Subsequently, nuclear PKM2 functions as a coactivator of HIF-1α to promote HIF-1α-dependent expression of glycolytic enzymes, such as GLUT1, HK2, PKM2, LDHA and PDK1, which in turn promotes aerobic glycolysis, immunosuppression of MDSCs and colorectal tumorigenesis (Fig. 9). Our studies emphasize a novel molecular mechanism by which *C. tropicalis* potentiates the immunosuppressive function of MDSCs and suggest that Syk-PKM2-HIF-1α-glycolysis axis may be a potential therapeutic target for CRC.

## Supplementary Information

**Additional file 1: Figure S1**. *C. tropicalis* promotes the immunosuppressive function of MDSCs through Dectin-3. Related to Figure 1. (A) The percentage of MDSCs (CD11b^+^Gr1^+^), G-MDSCs (CD11b^+^Ly6G^+^Ly6C^low^) and M-MDSCs (CD11b^+^Ly6G^−^Ly6C^+^) from bone marrow of WT and Clec4d-/- mice were determined by flow cytometry. (B) BM cells from WT and Clec4d^-/-^ mice were cultured with murine IL-6 and GM-CSF for 4 days. Then the percentage of MDSCs (CD11b^+^Gr1^+^), G-MDSCs (CD11b^+^Ly6G^+^Ly6C^low^) and M-MDSCs (CD11b^+^Ly6G^−^Ly6C^+^) were determined by flow cytometry. (C) WT MDSCs were stimulated with heat-inactivated *C.tropicalis* (MOI=2) for 24 h. Cell lysates were analyzed by immunoblotting for Dectin-3. (D) WT and Clec4d-/- MDSCs were stimulated with or without *C. tropicalis* (MOI=1) for 24 h. Then, MDSCs were collected and cocultured with CD8^+^ T cells at a 1:1 ratio in 96-well plates for 48 hours. IFNγ in the supernatants was measured by ELISA. (E and F) MDSCs from WT and Clec4d^-/-^ mice bearing AOM/DSS-induced CAC gavaged with or without *C. tropicalis* were collected. Then the expression of iNOS, COX2 and NOX2 in these collected MDSCs were determined by immunoblotting (E). In addition, these collected MDSCs were cocultured with CD8^+^ T cells labeled with 5 μM CFSE at a 1:1 ratio in 96-well plates for 72 hours. The proliferation of CD8^+^ T cells was measured by flow cytometry (F). The results shown here are expressed as the mean ± SEM. Each panel is a representative experiment of at least three independent biological replicates. *p <0.05, **p <0.01, ***p <0.001. The following statistical analyses were performed: unpaired Student’s t-test or one-way ANOVA where appropriate.** Figure S2**. Dectin-3 mediates *C. tropicalis*-induced glycolysis activation in MDSCs. Related to Figure 2. WT MDSCs were stimulated with heat-inactivated *C. tropicalis* (MOI=1) for 24h. Then unbiased metabolomics profiling was performed. Metabolites were identified by liquid chromatography-tandem mass spectrometry (LC-MS/MS) (n=5, mean ± SEM). (A) Principle component analysis (PCA) and (orthogonal) partial least-squares-discriminant analysis (O)PLS-DA were carried out to visualize the metabolic alterations in MDSCs among control group and *C. tropicalis* treated group. (B and C) Volcano Plot and Heatmap showing metabolites with significant changes as indicated in MDSCs.** Figure S3**. *C. tropicalis* induced Syk-mediated PKM2 Tyr105 phosphorylation and PKM2 nuclear translocation in MDSCs. Related to Figure 5. (A, B and D) WT MDSCs were stimulated with heat-inactivated *C. tropicalis* (MOI=2) in the presence or absence of Syk inhibitor R406 (as indicated concentration) for 18 h. Cell lysates were analyzed by immunoblotting for the indicated proteins. (C) WT MDSCs were stimulated with heat-inactivated *C. tropicalis* (MOI=2) in the presence or absence of Syk inhibitor R406 (2μM), then the ECAR level of these cells was measured by Agilent Seahorse XFe96 Analyzer. The results shown here are expressed as the mean ± SEM. Each panel is a representative experiment of at least three independent biological replicates. *p <0.05, **p <0.01, ***p <0.001. The following statistical analyses were performed: unpaired Student’s t-test or one-way ANOVA where appropriate.** Figure S4**. PKM2 nuclear translocation promotes HIF-1α-dependent glycolytic metabolism in MDSCs after *C. tropicalis* stimulation. Related to Figure 6. (A) WT MDSCs were stimulated with heat-inactivated *C.tropicalis* (MOI=2) in the presence or absence of FBP (20mM) for 6 h. Total RNA was extracted. iNOS, COX2 and NOX2 mRNA expression were measured by quantitative real-time PCR and normalized to that of the internal control, β-actin. (B) WT MDSCs were stimulated with heat-inactivated *C. tropicalis* (MOI=2) in the presence or absence of FBP (as indicated concentration) for 6 h. Cell lysates were analyzed by immunoblotting for the indicated proteins. (C) WT MDSCs were pretransfected with PKM2 siRNA for 24 hr prior to stimulation with heat-inactivated *C. tropicalis* (MOI=2) for 18 h. PKM2 expression were measured by immunoblotting. (D and E) WT MDSCs were pretransfected with PKM2 siRNA for 24 hr prior to stimulation with heat-inactivated *C. tropicalis* (MOI=2) for 6 h or 18 h. iNOS, COX2 and NOX2 expression were measured by quantitative real-time PCR (D) and immunoblotting (E). The results shown here are expressed as the mean ± SEM. Each panel is a representative experiment of at least three independent biological replicates. *p <0.05, **p <0.01, ***p <0.001. The following statistical analyses were performed: unpaired Student’s t-test or one-way ANOVA where appropriate.** Figure S5**. TEPP-46 ameliorates *C. tropicalis*-aggravated CAC. Related to Figure 7. (A) The proportion of MDSCs (CD11b^+^Gr1^+^) in colonic lamina propria (LP), bone marrow (BM) and spleen, as well as CD4^+^ and CD8^+^ T cells in spleen were measured by flow cytometry. (B) MDSCs from colonic lamina propria (LP) of mice bearing CAC were collected. Then, these collected MDSCs were cocultured with CD8^+^ T cells labeled with 5 μM CFSE at a 1:1 ratio in 96-well plates for 72 hours. The proliferation of CD8^+^ T cells was measured by flow cytometry. (C and E) The mRNA expressions of *Glut1*, *Hk2*, *Pkm2*, *Ldha*, *Nos2*, *Ptgs2*, *Cybb* in colon tumors tissues were detected by qPCR and normalized to that of the internal control, β-actin. (D and F) The protein levels of GLUT1, HK2, PKM2, LDHA, iNOS, COX2, NOX2 in colon tumors tissues were detected by western blots. The results shown here are expressed as the mean ± SEM. Each panel is a representative experiment of 6 independent biological replicates (n=6). *p <0.05, **p <0.01, ***p <0.001. The following statistical analyses were performed: unpaired Student’s t-test or one-way ANOVA where appropriate.** Figure S6**. The infiltration of MDSCs is positively correlated with the level of PKM2, PKM2 (p-Y105) and iNOS in patients with CRC. Related to Figure 8. (A) Illustrations of the gating strategy used in typical flow cytometry plots to reveal MDSCs in tumor tissue of patients with CRC. (B) The expression of PKM2, PKM2 (p-Y105) and iNOS in MDSCs (HLA-DR^−/low^ CD33^+^CD14^−/low^ CD66b^+^) from human CRC tissues (n=50) and adjacent nontumor tissues (n=50) were measure by flow cytometry. The results shown here are expressed as the mean ± SEM. *p <0.05, **p <0.01, ***p <0.001. The following statistical analyses were performed: unpaired Student’s t test.** Table S1**. Primer sequences for qRT-PCR.** Table S2**. Antibody for Western blot analysis and IHC.** Table S3**. Antibody for FACS analysis.** Table S4**. siRNA Sequence

**Additional file 2**. Raw data of this paper

## Data Availability

The RNA sequencing data from this study have been deposited in the NCBI Sequence Read Archive (SRA) database under the accession number: PRJNA854306. Metabolomics data from this study have been deposited to the EMBL-EBI MetaboLights database (DOI: 10.1093/nar/gkz1019, PMID:31,691,833) with the identifier MTBLS5276. The complete dataset can be accessed here https://www.ebi.ac.uk/metabolights/MTBLS5276. Moreover, the raw data generated for this study are available on request to the corresponding author.

## Abbreviations

CRC: Colorectal cancer
ECAR: Extracellular acidification rate
iNOS: Inducible nitric oxide synthase
COX2: cyclooxygenase 2
NOX2: NADPH oxidase 2
NO: Nitric oxide
ROS: Reactive oxygen species
Syk: Spleen tyrosine kinase
PKM2: Pyruvate Kinase M2
MDSCs: Myeloid-derived suppressor cells
HIF-1α: Hypoxia-inducible factor 1α
GLUT1: Glucose transporter 1
HK2: Hexokinase 2
LDHA: Lactate dehydrogenase A
PDK1: Pyruvate dehydrogenase kinase 1
CAC: Colitis-associated colon cancer
PCNA: Proliferating cell nuclear antigen
COAD: Colon adenocarcinoma

## References

[1] Arnold M, Sierra MS, Laversanne M, Soerjomataram I, Jemal A, Bray F (2017). Global patterns and trends in colorectal cancer incidence and mortality. Gut.

[2] Wong MCS, Huang J, Lok V, Wang J, Fung F, Ding H (2021). Differences in incidence and mortality trends of colorectal cancer worldwide based on sex, age, and anatomic location. Clin Gastroenterol Hepatol..

[3] Dahlhamer JM, Zammitti EP, Ward BW, Wheaton AG, Croft JB (2016). Prevalence of inflammatory bowel disease among adults aged ≥ 18 Years - United States, 2015. MMWR Morb Mortal Wkly Rep.

[4] Olén O, Erichsen R, Sachs MC, Pedersen L, Halfvarson J, Askling J (2020). Colorectal cancer in ulcerative colitis: a Scandinavian population-based cohort study. Lancet.

[5] Man SM, Zhu Q, Zhu L, Liu Z, Karki R, Malik A (2015). Critical role for the DNA sensor AIM2 in stem cell proliferation and cancer. Cell.

[6] Malik A, Sharma D, Zhu Q, Karki R, Guy CS, Vogel P (2016). IL-33 regulates the IgA-microbiota axis to restrain IL-1α-dependent colitis and tumorigenesis. J Clin Invest.

[7] Sokol H, Leducq V, Aschard H, Pham HP, Jegou S, Landman C (2017). Fungal microbiota dysbiosis in IBD. Gut.

[8] Iliev ID, Funari VA, Taylor KD, Nguyen Q, Reyes CN, Strom SP (2012). Interactions between commensal fungi and the C-type lectin receptor Dectin-1 influence colitis. Science.

[9] Wang T, Pan D, Zhou Z, You Y, Jiang C, Zhao X (2016). Dectin-3 deficiency promotes colitis development due to impaired antifungal innate immune responses in the gut. PLoS Pathog.

[10] Wang T, Fan C, Yao A, Xu X, Zheng G, You Y (2018). The adaptor protein CARD9 protects against colon cancer by restricting mycobiota-mediated expansion of myeloid-derived suppressor cells. Immunity..

[11] Gabrilovich DI, Nagaraj S (2009). Myeloid-derived suppressor cells as regulators of the immune system. Nat Rev Immunol.

[12] Gabrilovich DI, Ostrand-Rosenberg S, Bronte V (2012). Coordinated regulation of myeloid cells by tumours. Nat Rev Immunol.

[13] Condamine T, Gabrilovich DI (2011). Molecular mechanisms regulating myeloid-derived suppressor cell differentiation and function. Trends Immunol.

[14] Kumar V, Patel S, Tcyganov E, Gabrilovich DI (2016). The nature of myeloid-derived suppressor cells in the tumor microenvironment. Trends Immunol.

[15] Romani L (2011). Immunity to fungal infections. Nat Rev Immunol.

[16] Hardison SE, Brown GD (2012). C-type lectin receptors orchestrate antifungal immunity. Nat Immunol.

[17] Navarro-Arias MJ, Hernandez-Chavez MJ, Garcia-Carnero LC, Amezcua-Hernandez DG, Lozoya-Perez NE, Estrada-Mata E (2019). Differential recognition of Candida tropicalis, Candida guilliermondii, Candida krusei, and Candida auris by human innate immune cells. Infect Drug Resist.

[18] Zhu LL, Zhao XQ, Jiang C, You Y, Chen XP, Jiang YY (2013). C-type lectin receptors Dectin-3 and Dectin-2 form a heterodimeric pattern-recognition receptor for host defense against fungal infection. Immunity.

[19] Zhu Y, Shi T, Lu X, Xu Z, Qu J, Zhang Z (2021). Fungal-induced glycolysis in macrophages promotes colon cancer by enhancing innate lymphoid cell secretion of IL-22. EMBO J..

[20] Krawczyk CM, Holowka T, Sun J, Blagih J, Amiel E, DeBerardinis RJ (2010). Toll-like receptor-induced changes in glycolytic metabolism regulate dendritic cell activation. Blood.

[21] Kelly B, O’Neill LA (2015). Metabolic reprogramming in macrophages and dendritic cells in innate immunity. Cell Res.

[22] O’Neill LA, Pearce EJ (2016). Immunometabolism governs dendritic cell and macrophage function. J Exp Med.

[23] Ip WKE, Hoshi N, Shouval DS, Snapper S, Medzhitov R (2017). Anti-inflammatory effect of IL-10 mediated by metabolic reprogramming of macrophages. Science.

[24] Van den Bossche J, O’Neill LA, Menon D (2017). Macrophage immunometabolism: where are we (going)?. Trends Immunol.

[25] Guak H, Al Habyan S, Ma EH, Aldossary H, Al-Masri M, Won SY (2018). Glycolytic metabolism is essential for CCR7 oligomerization and dendritic cell migration. Nat Commun.

[26] Vander Heiden MG, DeBerardinis RJ (2017). Understanding the intersections between metabolism and cancer biology. Cell.

[27] Reinfeld BI, Madden MZ, Wolf MM, Chytil A, Bader JE, Patterson AR (2021). Cell-programmed nutrient partitioning in the tumour microenvironment. Nature.

[28] Hu C, Pang B, Lin G, Zhen Y, Yi H (2020). Energy metabolism manipulates the fate and function of tumour myeloid-derived suppressor cells. Br J Cancer.

[29] Leone RD, Powell JD (2020). Metabolism of immune cells in cancer. Nat Rev Cancer.

[30] Ji J, Xu J, Zhao S, Liu F, Qi J, Song Y (2016). Myeloid-derived suppressor cells contribute to systemic lupus erythaematosus by regulating differentiation of Th17 cells and Tregs. Clin Sci (Lond).

[31] Li D, Qi J, Wang J, Pan Y, Li J, Xia X (2019). Protective effect of dihydroartemisinin in inhibiting senescence of myeloid-derived suppressor cells from lupus mice via Nrf2/HO-1 pathway. Free Radic Biol Med.

[32] Hornbeck PV, Zhang B, Murray B, Kornhauser JM, Latham V, Skrzypek E (2015). PhosphoSitePlus, 2014: mutations, PTMs and recalibrations. Nucleic Acids Res.

[33] Tang Z, Kang B, Li C, Chen T, Zhang Z (2019). GEPIA2: an enhanced web server for large-scale expression profiling and interactive analysis. Nucleic Acids Res.

[34] Kargl J, Haybaeck J, Stancic A, Andersen L, Marsche G, Heinemann A (2013). O-1602, an atypical cannabinoid, inhibits tumor growth in colitis-associated colon cancer through multiple mechanisms. J Mol Med (Berl).

[35] Yeung SJ, Pan J, Lee MH (2008). Roles of p53, MYC and HIF-1 in regulating glycolysis - the seventh hallmark of cancer. Cell Mol Life Sci.

[36] Luo W, Hu H, Chang R, Zhong J, Knabel M, O’Meally R (2011). Pyruvate kinase M2 is a PHD3-stimulated coactivator for hypoxia-inducible factor 1. Cell.

[37] Everts B, Amiel E, van der Windt GJ, Freitas TC, Chott R, Yarasheski KE (2012). Commitment to glycolysis sustains survival of NO-producing inflammatory dendritic cells. Blood.

[38] Lawless SJ, Kedia-Mehta N, Walls JF, McGarrigle R, Convery O, Sinclair LV (2017). Glucose represses dendritic cell-induced T cell responses. Nat Commun.

[39] Thwe PM, Amiel E (2018). The role of nitric oxide in metabolic regulation of Dendritic cell immune function. Cancer Lett.

[40] Saijo S, Ikeda S, Yamabe K, Kakuta S, Ishigame H, Akitsu A (2010). Dectin-2 recognition of alpha-mannans and induction of Th17 cell differentiation is essential for host defense against Candida albicans. Immunity.

[41] Strasser D, Neumann K, Bergmann H, Marakalala MJ, Guler R, Rojowska A (2012). Syk kinase-coupled C-type lectin receptors engage protein kinase C-delta to elicit Card9 adaptor-mediated innate immunity. Immunity.

[42] Yang W, Zheng Y, Xia Y, Ji H, Chen X, Guo F (2012). ERK1/2-dependent phosphorylation and nuclear translocation of PKM2 promotes the Warburg effect. Nat Cell Biol.

[43] Xu Q, Tu J, Dou C, Zhang J, Yang L, Liu X (2017). HSP90 promotes cell glycolysis, proliferation and inhibits apoptosis by regulating PKM2 abundance via Thr-328 phosphorylation in hepatocellular carcinoma. Mol Cancer.

[44] Anastasiou D, Yu Y, Israelsen WJ, Jiang JK, Boxer MB, Hong BS (2012). Pyruvate kinase M2 activators promote tetramer formation and suppress tumorigenesis. Nat Chem Biol.

[45] Palsson-McDermott EM, Curtis AM, Goel G, Lauterbach MA, Sheedy FJ, Gleeson LE (2015). Pyruvate kinase M2 regulates Hif-1alpha activity and IL-1beta induction and is a critical determinant of the warburg effect in LPS-activated macrophages. Cell Metab.

[46] Angiari S, Runtsch MC, Sutton CE, Palsson-McDermott EM, Kelly B, Rana N (2020). Pharmacological activation of pyruvate kinase M2 inhibits CD4(+) T cell pathogenicity and suppresses autoimmunity. Cell Metab.

[47] Deng Y, Cheng J, Fu B, Liu W, Chen G, Zhang Q (2017). Hepatic carcinoma-associated fibroblasts enhance immune suppression by facilitating the generation of myeloid-derived suppressor cells. Oncogene.

[48] Rieber N, Singh A, Oz H, Carevic M, Bouzani M, Amich J (2015). Pathogenic fungi regulate immunity by inducing neutrophilic myeloid-derived suppressor cells. Cell Host Microbe.

[49] Di Martino L, De Salvo C, Buela KA, Hager C, Ghannoum M, Osme A (2022). Candida tropicalis infection modulates the gut microbiome and confers enhanced susceptibility to colitis in mice. Cell Mol Gastroenterol Hepatol.

[50] Zhu Y, Shi T, Lu X, Xu Z, Qu J, Zhang Z (2021). Fungal-induced glycolysis in macrophages promotes colon cancer by enhancing innate lymphoid cell secretion of IL-22. EMBO J.

[51] Jian SL, Chen WW, Su YC, Su YW, Chuang TH, Hsu SC (2017). Glycolysis regulates the expansion of myeloid-derived suppressor cells in tumor-bearing hosts through prevention of ROS-mediated apoptosis. Cell Death Dis.

[52] Deng Y, Yang J, Luo F, Qian J, Liu R, Zhang D (2018). mTOR-mediated glycolysis contributes to the enhanced suppressive function of murine tumor-infiltrating monocytic myeloid-derived suppressor cells. Cancer Immunol Immunother.

[53] Veglia F, Tyurin VA, Blasi M, De Leo A, Kossenkov AV, Donthireddy L (2019). Fatty acid transport protein 2 reprograms neutrophils in cancer. Nature.

[54] Yan D, Adeshakin AO, Xu M, Afolabi LO, Zhang G, Chen YH (2019). Lipid metabolic pathways confer the immunosuppressive function of myeloid-derived suppressor cells in tumor. Front Immunol.

[55] Adeshakin AO, Liu W, Adeshakin FO, Afolabi LO, Zhang M, Zhang G (2021). Regulation of ROS in myeloid-derived suppressor cells through targeting fatty acid transport protein 2 enhanced anti-PD-L1 tumor immunotherapy. Cell Immunol.

[56] Palmieri EM, Gonzalez-Cotto M, Baseler WA, Davies LC, Ghesquiere B, Maio N (2020). Nitric oxide orchestrates metabolic rewiring in M1 macrophages by targeting aconitase 2 and pyruvate dehydrogenase. Nat Commun.

[57] Hitosugi T, Kang S, Vander Heiden MG, Chung TW, Elf S, Lythgoe K (2009). Tyrosine phosphorylation inhibits PKM2 to promote the Warburg effect and tumor growth. Sci Signal.

[58] Azoitei N, Becher A, Steinestel K, Rouhi A, Diepold K, Genze F (2016). PKM2 promotes tumor angiogenesis by regulating HIF-1alpha through NF-kappaB activation. Mol Cancer.

[59] Zhang Z, Deng X, Liu Y, Liu Y, Sun L, Chen F (2019). PKM2, function and expression and regulation. Cell Biosci.

