# Supplementary material for: Gut fungi enhances immunosuppressive function of myeloid-derived suppressor cells by activating PKM2-dependent glycolysis to promote colorectal tumorigenesis
Source: Exp Hematol Oncol. 2022 Nov 8;11:88. doi: 10.1186/s40164-022-00334-6 (PMC9644472; doi:10.1186/s40164-022-00334-6)
Supplement: Supplementary file 1 — Additional file 1: Figure S1. C. tropicalis promotes the immunosuppressive function of MDSCs through Dectin-3. Related to Figure 1. (A) The percentage of MDSCs (CD11b+Gr1+), G-MDSCs (CD11b+Ly6G+Ly6Clow) and M-MDSCs (CD11b+Ly6G−Ly6C+) from bone marrow of WT and Clec4d-/- mice were determined by flow cytometry. (B) BM cells from WT and Clec4d-/- mice were cultured with murine IL-6 and GM-CSF for 4 days. Then the percentage of MDSCs (CD11b+Gr1+), G-MDSCs (CD11b+Ly6G+Ly6Clow) and M-MDSCs (CD11b+Ly6G−Ly6C+) were determined by flow cytometry. (C) WT MDSCs were stimulated with heat-inactivated C.tropicalis (MOI=2) for 24 h. Cell lysates were analyzed by immunoblotting for Dectin-3. (D) WT and Clec4d-/- MDSCs were stimulated with or without C. tropicalis (MOI=1) for 24 h. Then, MDSCs were collected and cocultured with CD8+ T cells at a 1:1 ratio in 96-well plates for 48 hours. IFNγ in the supernatants was measured by ELISA. (E and F) MDSCs from WT and Clec4d-/- mice bearing AOM/DSS-induced CAC gavaged with or without C. tropicalis were collected. Then the expression of iNOS, COX2 and NOX2 in these collected MDSCs were determined by immunoblotting (E). In addition, these collected MDSCs were cocultured with CD8+ T cells labeled with 5 μM CFSE at a 1:1 ratio in 96-well plates for 72 hours. The proliferation of CD8+ T cells was measured by flow cytometry (F). The results shown here are expressed as the mean ± SEM. Each panel is a representative experiment of at least three independent biological replicates. *p <0.05, **p <0.01, ***p <0.001. The following statistical analyses were performed: unpaired Student’s t-test or one-way ANOVA where appropriate. Figure S2. Dectin-3 mediates C. tropicalis-induced glycolysis activation in MDSCs. Related to Figure 2. WT MDSCs were stimulated with heat-inactivated C. tropicalis (MOI=1) for 24h. Then unbiased metabolomics profiling was performed. Metabolites were identified by liquid chromatography-tandem mass spectrometry (LC-MS/MS) (n=5, me [file 40164_2022_334_MOESM1_ESM.docx]

**Supplementary Information**

**Gut fungi enhances immunosuppressive function of myeloid-derived suppressor cells by activating PKM2-dependent glycolysis to promote colorectal tumorigenesis**

Zhiyong Zhang *et al*.

**Supplementary figures**


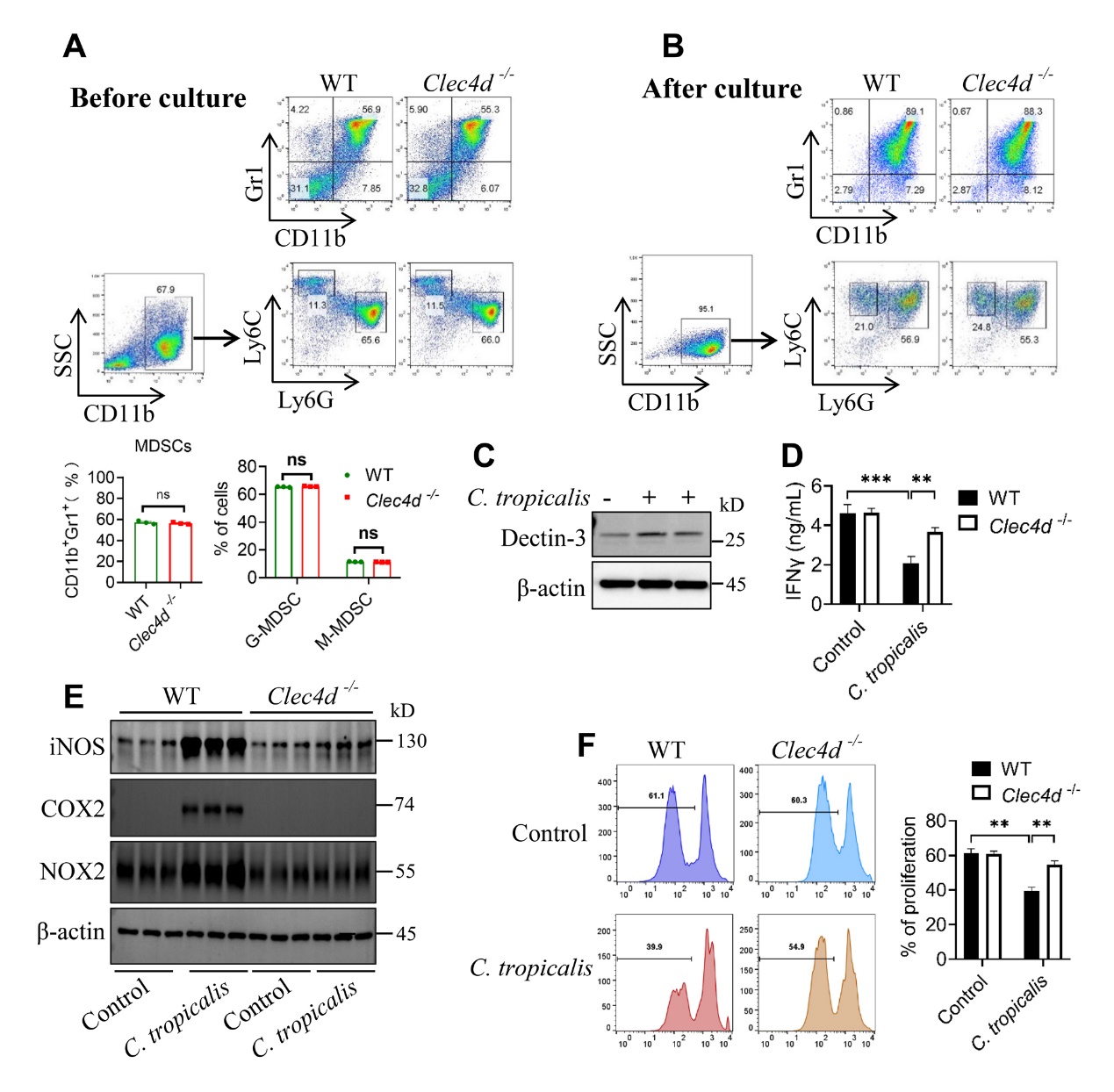


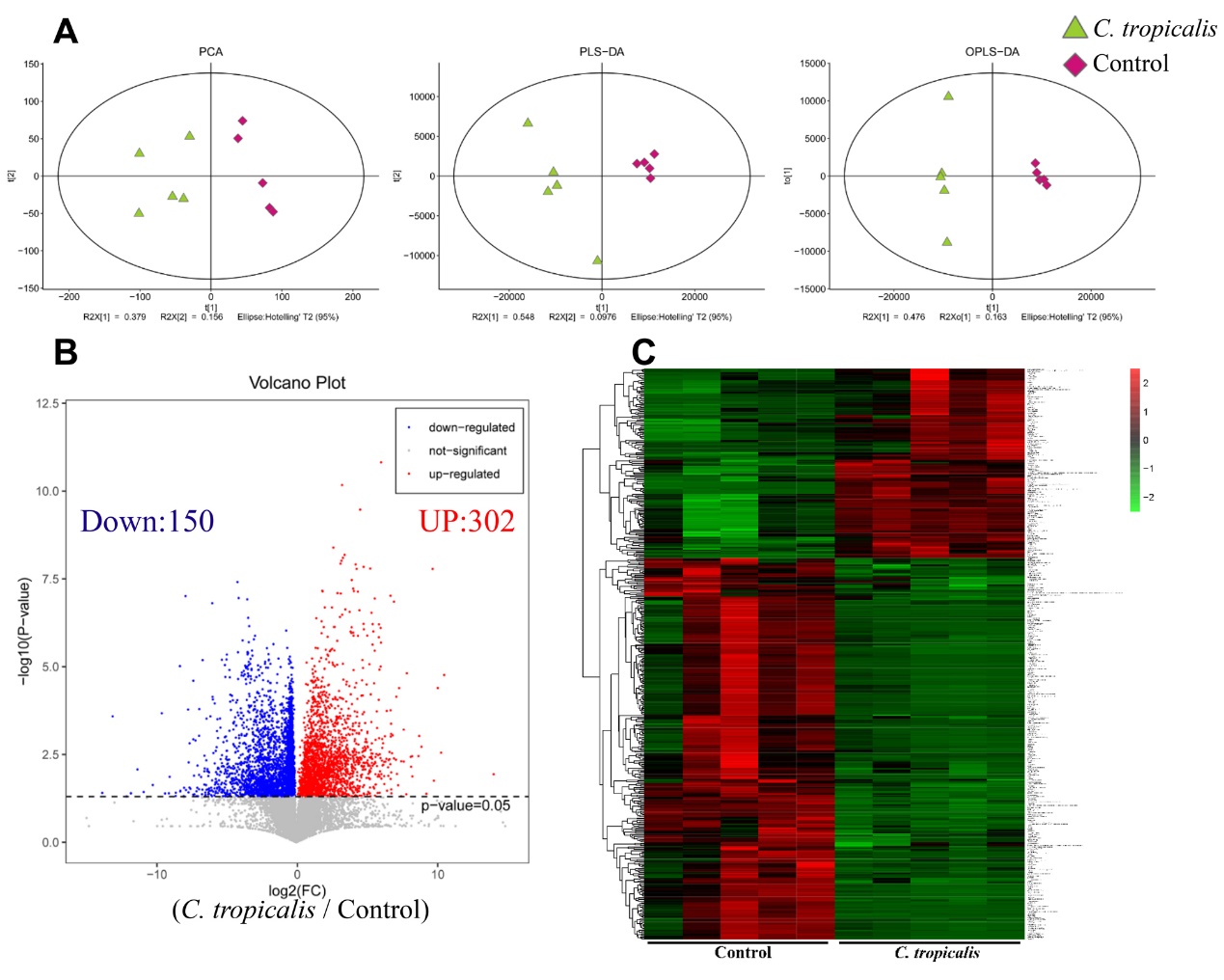


**Figure S2.** **Dectin-3 mediates** ***C. tropicalis*-induced glycolysis** **activation in MDSCs.** **Related to Figure 2.** WT MDSCs were stimulated with heat-inactivated *C. tropicalis* (MOI=1) for 24h. Then unbiased metabolomics profiling was performed. Metabolites were identified by liquid chromatography-tandem mass spectrometry (LC-MS/MS) (n=5, mean ± SEM). (A) Principle component analysis (PCA) and (orthogonal) partial least-squares-discriminant analysis (O)PLS-DA were carried out to visualize the metabolic alterations in MDSCs among control group and *C. tropicalis* treated group. (B and C) Volcano Plot and Heatmap showing metabolites with significant changes as indicated in MDSCs.


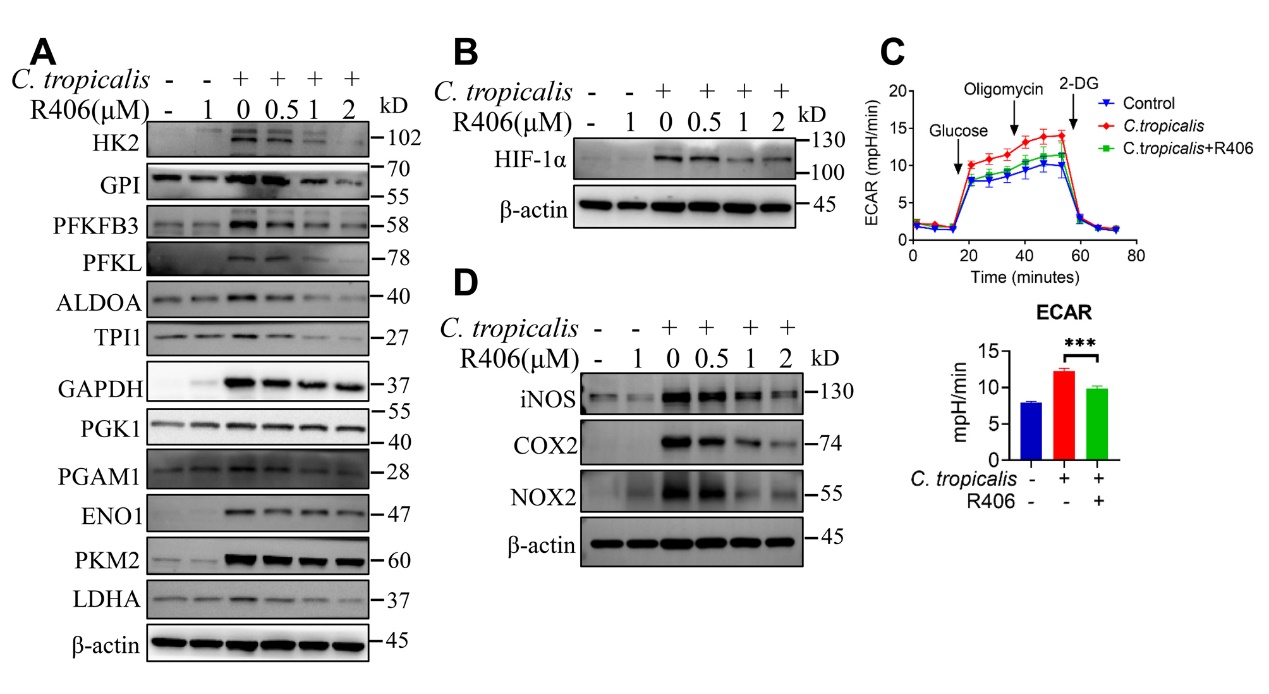


**Figure S3. *C. tropicalis* induced Syk-mediated PKM2 Tyr105 phosphorylation and PKM2 nuclear translocation in MDSCs. Related to Figure 5.** (A, B and D) WT MDSCs were stimulated with heat-inactivated *C. tropicalis* (MOI=2) in the presence or absence of Syk inhibitor R406 (as indicated concentration) for 18 h. Cell lysates were analyzed by immunoblotting for the indicated proteins. (C) WT MDSCs were stimulated with heat-inactivated *C. tropicalis* (MOI=2) in the presence or absence of Syk inhibitor R406 (2μM), then the ECAR level of these cells was measured by Agilent Seahorse XFe96 Analyzer. The results shown here are expressed as the mean ± SEM. Each panel is a representative experiment of at least three independent biological replicates. *p <0.05, **p <0.01, ***p <0.001. The following statistical analyses were performed: unpaired Student’s *t*-test or one-way ANOVA where appropriate.


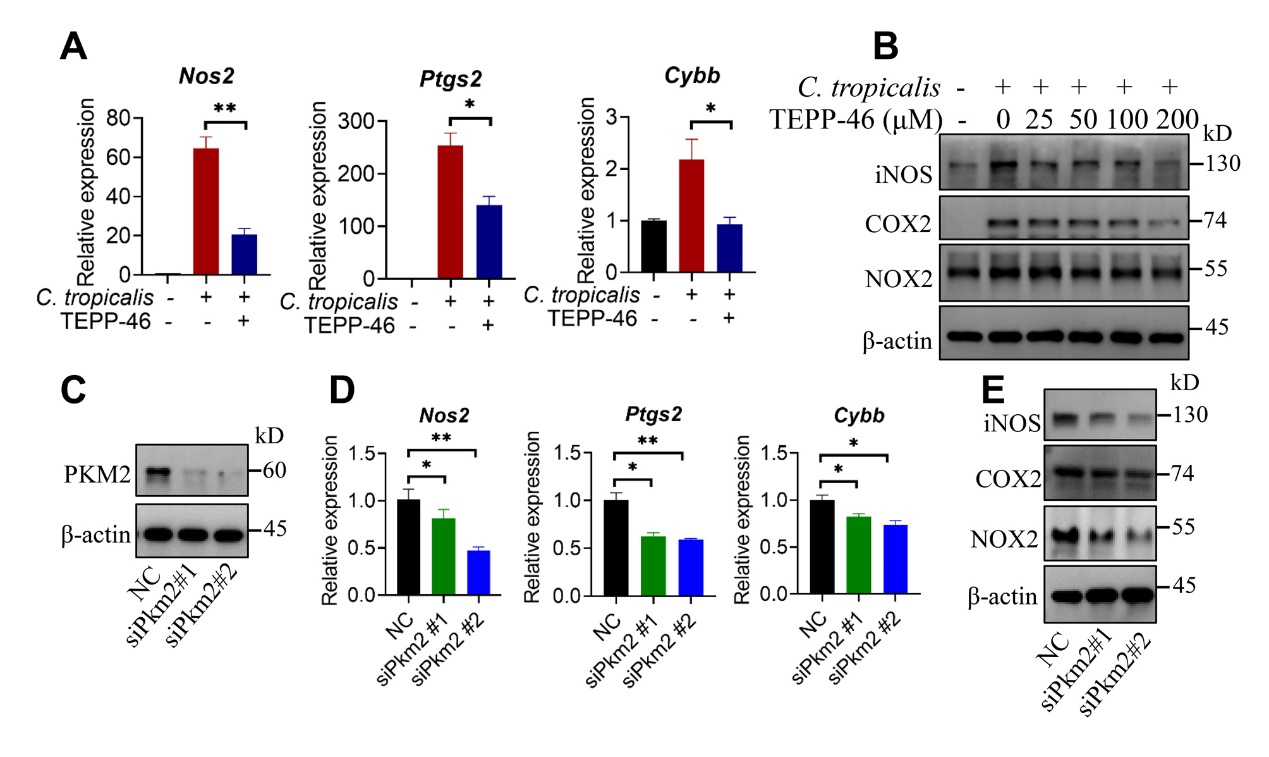


**Figure S4.** **PKM2 nuclear translocation promotes HIF-1α-dependent glycolytic metabolism in MDSCs after *C. tropicalis* stimulation. Related to Figure 6.** (A) WT MDSCs were stimulated with heat-inactivated *C.tropicalis* (MOI=2) in the presence or absence of FBP (20mM) for 6 h. Total RNA was extracted. iNOS, COX2 and NOX2 mRNA expression were measured by quantitative real-time PCR and normalized to that of the internal control, *β-actin*. (B) WT MDSCs were stimulated with heat-inactivated *C. tropicalis* (MOI=2) in the presence or absence of FBP (as indicated concentration) for 6 h. Cell lysates were analyzed by immunoblotting for the indicated proteins. (C) WT MDSCs were pretransfected with PKM2 siRNA for 24 hr prior to stimulation with heat-inactivated *C. tropicalis* (MOI=2) for 18 h. PKM2 expression were measured by immunoblotting. (D and E) WT MDSCs were pretransfected with PKM2 siRNA for 24 hr prior to stimulation with heat-inactivated *C. tropicalis* (MOI=2) for 6 h or 18 h. iNOS, COX2 and NOX2 expression were measured by quantitative real-time PCR (D) and immunoblotting (E). The results shown here are expressed as the mean ± SEM. Each panel is a representative experiment of at least three independent biological replicates. *p <0.05, **p <0.01, ***p <0.001. The following statistical analyses were performed: unpaired Student’s *t*-test or one-way ANOVA where appropriate.


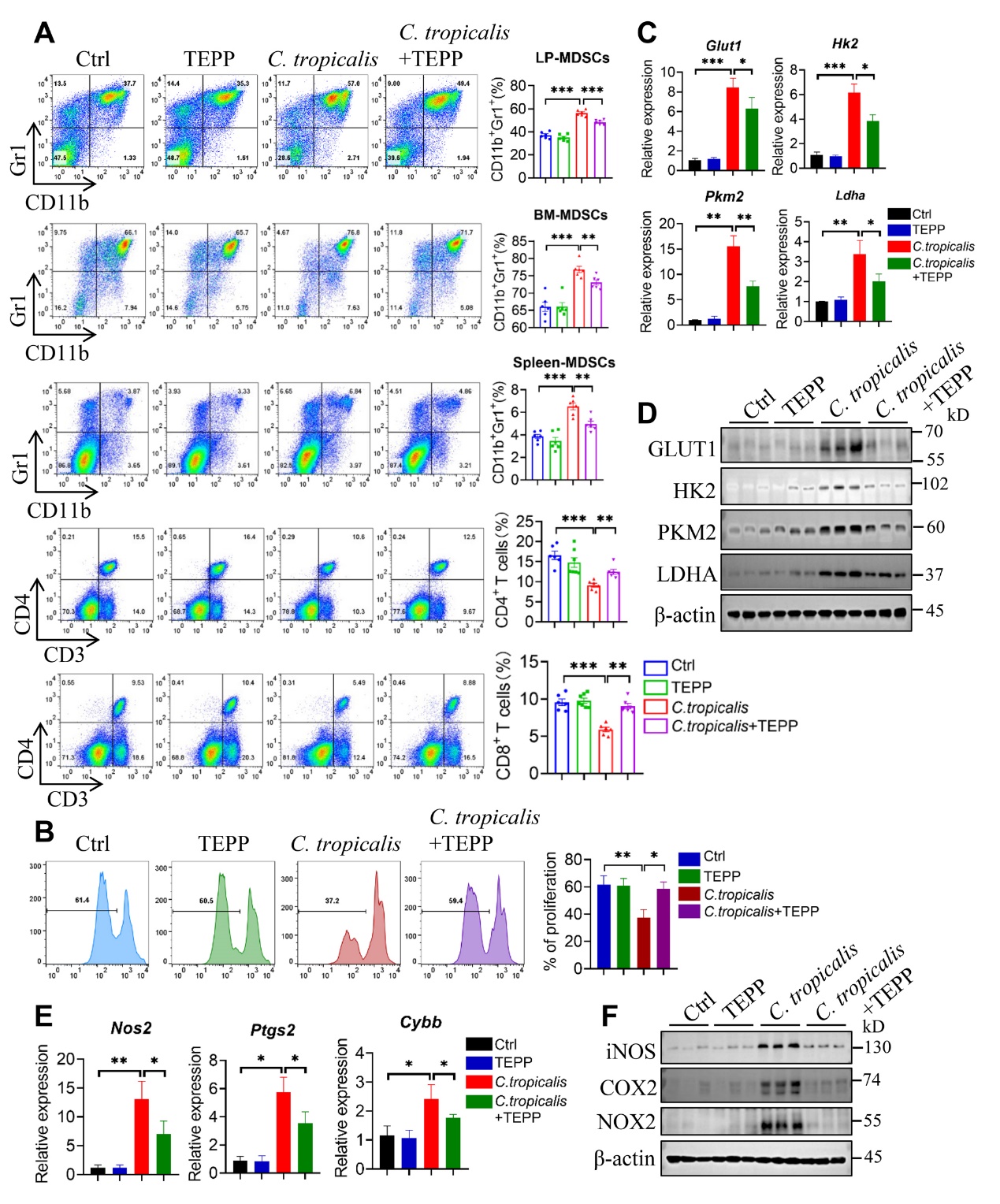


**Figure S5.** **TEPP-46 ameliorates *C. tropicalis*-aggravated CAC. Related to Figure 7.** (A) The proportion of MDSCs (CD11b^+^Gr1^+^) in colonic lamina propria (LP), bone marrow (BM) and spleen, as well as CD4^+^ and CD8^+^ T cells in spleen were measured by flow cytometry. (B) MDSCs from colonic lamina propria (LP) of mice bearing CAC were collected. Then, these collected MDSCs were cocultured with CD8^+^ T cells labeled with 5 μM CFSE at a 1:1 ratio in 96-well plates for 72 hours. The proliferation of CD8^+^ T cells was measured by flow cytometry. (C and E) The mRNA expressions of *Glut1*, *Hk2*, *Pkm2*, *Ldha*, *Nos2*, *Ptgs2*, *Cybb* in colon tumors tissues were detected by qPCR and normalized to that of the internal control, *β-actin*. (D and F) The protein levels of GLUT1, HK2, PKM2, LDHA, iNOS, COX2, NOX2 in colon tumors tissues were detected by western blots. The results shown here are expressed as the mean ± SEM. Each panel is a representative experiment of 6 independent biological replicates (n=6). *p <0.05, **p <0.01, ***p <0.001. The following statistical analyses were performed: unpaired Student’s *t*-test or one-way ANOVA where appropriate.


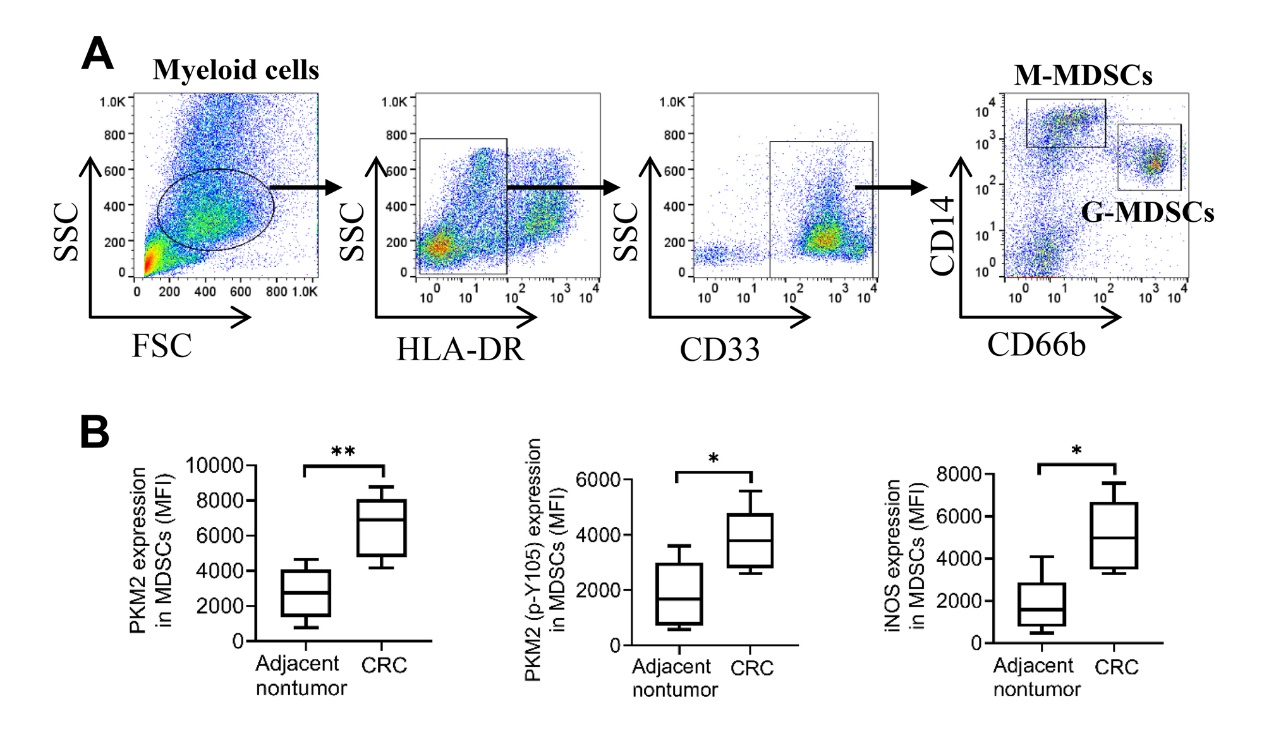


**Figure S6.** **The infiltration of MDSCs is positively correlated with the level of PKM2, PKM2 (p-Y105) and iNOS in patients with CRC.** **Related to Figure 8.** (A) Illustrations of the gating strategy used in typical flow cytometry plots to reveal MDSCs in tumor tissue of patients with CRC. (B) The expression of PKM2, PKM2 (p-Y105) and iNOS in MDSCs (HLA-DR^−/low^ CD33^+^ CD14^−/low^ CD66b^+^) from human CRC tissues (n=50) and adjacent nontumor tissues (n=50) were measure by flow cytometry. The results shown here are expressed as the mean ± SEM. *p <0.05, **p <0.01, ***p <0.001. The following statistical analyses were performed: unpaired Student’s t test.

**Supplementary Tables**

| **Gene (Mouse)** | **Forward Primer (5’→3’)** | **Reverse Primer (5’→3’)** |
| --- | --- | --- |
| *β-actin* | ATTGTTACCAACTGGGACGACATG | CTTCATGAGGTAGTCTGTCAGGTC |
| *Nos2* | GTTCTCAGCCCAACAATACAAGA | GTGGACGGGTCGATGTCAC |
| *Ptgs2* | TACAGGAGAGAAGGAAATGGC | TTGAGGAGAACAGATGGGATT |
| *Cybb* | TGCCCCAAGGTATCCAAGTT | CCTCCGTCCAGTCTCCCACA |
| *Hif-1α* | GACAATAGCTTCGCAGAATGC | TCGTAACTGGTCAGCTGTGG |
| *Glut1* | GCAGTTCGGCTATAACACTGG | GCGGTGGTTCCATGTTTGATTG |
| *Hk2* | CCTGCTACAGGTCCGAGCCATCTT | GAGGATGAAGCTTGTACAGTGTCC |
| *Gpi* | AACCGGCCGACCAACTCAATTGTG | TGCCGTCCAGCTCTGGCTCAATTT |
| *Pfkl* | AATGTGCTGGGCCACTTGCAGCAG | TGACCGGACTGAAGGCCACTACCT |
| *Pfkfb3* | CCCAGAGCCGGGTACAGAA | GGGGAGTTGGTCAGCTTCG |
| *Aldoa* | ATGAGGAGATTGCCATGGCAACGG | TTTAGAGCAGAGGCCTGCAGGGCT |
| *Tpi1* | CCAGGAAGTTCTTCGTTGGGG | CAAAGTCGATGTAAGCGGTGG |
| *Gapdh* | GGGACAAGGATAGTCATTTTGGGG | TGTCATTGAGAGCAATGCCAGCCC |
| *Pgk1* | AGACTGGCCAAGCTACTGTGGCCT | GAAGTGGCTTTCACCACCTCATCC |
| *Pgam1* | TACGCAGACCTTACTGAAGACCAG | AGCTCCATGATGGCCTCTTCTGAG |
| *Eno1* | ACCAACCCTAAGCGGATTGCCAAG | AGTCTTGATCTGCCCAGTGCAGAG |
| *Pkm2* | TCGCATGCAGCACCTGATT | CCTCGAATAGCTGCAAGTGGTA |
| *Ldha* | GCAGACAAGGAGCAGTGGAAGGAG | ACACTGAGGAAGACATCCTCATTG |
| *Pdk1* | GGACTTCGGGTCAGTGAATGC | TCCTGAGAAGATTGTCGGGGA |

**Table S1** **Primer sequences for qRT-PCR**

**Table S2 Antibody for Western blot analysis and IHC**

| **Antibody** | **Species** | **Catalog number** | **Distributor** |
| --- | --- | --- | --- |
| β-actin | Rabbit | 8457 | Cell Signaling Technology |
| PCNA | Rabbit | 13110 | Cell Signaling Technology |
| iNOS | Rabbit | 18985-1-AP | Proteintech |
| COX2 | Rabbit | 12282 | Cell Signaling Technology |
| NOX2 | Rabbit | 19013-1-AP | Proteintech |
| total STAT3 | Rabbit | 12640 | Cell Signaling Technology |
| phospho-STAT3 | Rabbit | 9145 | Cell Signaling Technology |
| PD-L1 | Rabbit | 13684 | Cell Signaling Technology |
| Dectin-3 | Rabbit polyclonal antibody | ab175021 | Abcam |
| HIF-1α | Rabbit polyclonal antibody | YT2133 | Immunoway |
| GLUT1 | Rabbit | 12939 | Cell Signaling Technology |
| HK2 | Rabbit | 2867 | Cell Signaling Technology |
| GPI | Rabbit | 15171-1-AP | Proteintech |
| PFKL | Rabbit | ab181064 | Abcam |
| PFKFB3 | Rabbit polyclonal antibody | 13763-1-AP | Proteintech |
| ALDOA | Rabbit | 8060 | Cell Signaling Technology |
| TPI1 | Rabbit monoclonal antibody | 67515-1-Ig | Proteintech |
| GAPDH | Rabbit polyclonal antibody | 10494-1-AP | Proteintech |
| PGK1 | Rabbit polyclonal antibody | 17811-1-AP | Proteintech |
| PGAM1 | Rabbit | 12098 | Cell Signaling Technology |
| ENO1 | Rabbit | 3810 | Cell Signaling Technology |
| PKM2 | Rabbit | 4053 | Cell Signaling Technology |
| phospho-PKM2 (Tyr105) | Rabbit | 12821 | Signalway Antibody |
| LDHA | Rabbit | 2012 | Cell Signaling Technology |
| PDHK1 | Rabbit | 3820 | Cell Signaling Technology |
| total Syk | Rabbit | 13198 | Cell Signaling Technology |
| phospho-Syk | Rabbit | 2710 | Cell Signaling Technology |
| PKM2 (D78A4) XP® Rabbit mAb (Sepharose® Bead Conjugate) | Rabbit | 13266 | Cell Signaling Technology |
| Rabbit (DA1E) mAb IgG XP® Isotype Control (Sepharose® Bead Conjugate) | Rabbit | 3423 | Cell Signaling Technology |
| Ki-67 | Rabbit | 12202S | Cell Signaling Technology |
| phospho-PKM2  (Tyr105) | Rabbit | bs-3334R | Bioss |
| iNOS | Rabbit | SAB4502012 | Sigma |

**Table S3** **Antibody for FACS analysis**

| **Antibody** | **Species** | **Catalog number** | **Distributor** |
| --- | --- | --- | --- |
| PE/Cy7-conjugated anti-mouse CD45 | mouse | 103114 | BioLegend |
| FITC-conjugated anti-mouse/human CD11b | mouse/human | 101206 | BioLegend |
| APC-conjugated anti-mouse Ly-6G/Ly-6C (Gr-1) | mouse | 108412 | BioLegend |
| PE-conjugated anti-mouse Ly-6G | mouse | 127608 | BioLegend |
| APC-conjugated anti-mouse Ly-6C | mouse | 128016 | BioLegend |
| FITC-conjugated anti-mouse CD3 | mouse | 100203 | BioLegend |
| ANTI-MO CD4 RMA-5 APC | mouse | 17-0042-81 | eBioscience |
| APC-conjugated anti-mouse CD8a | mouse | 100712 | BioLegend |
| PerCP/Cyanine5.5 anti-human HLA-DR antibody | human | 307629 | BioLegend |
| FITC anti-human CD33 antibody | human | 303303 | BioLegend |
| APC anti-human CD14 antibody | human | 301807 | BioLegend |
| PE anti-human CD66b antibody | human | 305105 | BioLegend |

**Table S4** **siRNA Sequence**

| Gene | siRNA | **Sequence** |
| --- | --- | --- |
| Mouse *Hk2* | siRNA#1 | CCAAAGATGTCTCGGATAT |
|  | siRNA#2 | GAAGGATGAAGGTGGAAAT |
|  | siRNA#3 | GCAACATCCTGATCGATTT |
| Mouse *Pkm2* | siRNA#1 | CCCTGTGCTGTGTAAGGAT |
|  | siRNA#2 | GATGTCGACCTTCGTGTAA |
|  | siRNA#3 | TCCTATCATTGCCGTGACT |
