# Supplementary material for: Gut fungi enhances immunosuppressive function of myeloid-derived suppressor cells by activating PKM2-dependent glycolysis to promote colorectal tumorigenesis
Source: Exp Hematol Oncol. 2022 Nov 8;11:88. doi: 10.1186/s40164-022-00334-6 (PMC9644472; doi:10.1186/s40164-022-00334-6)

Figure 1D

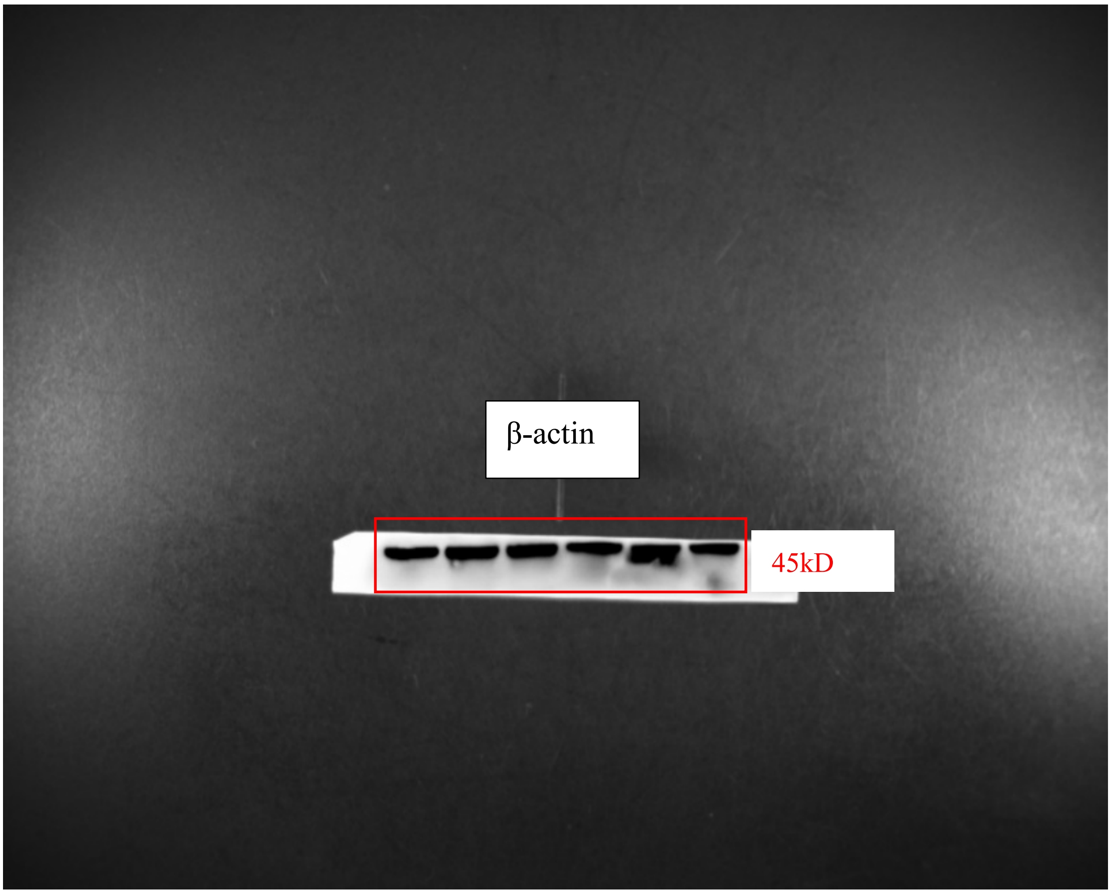

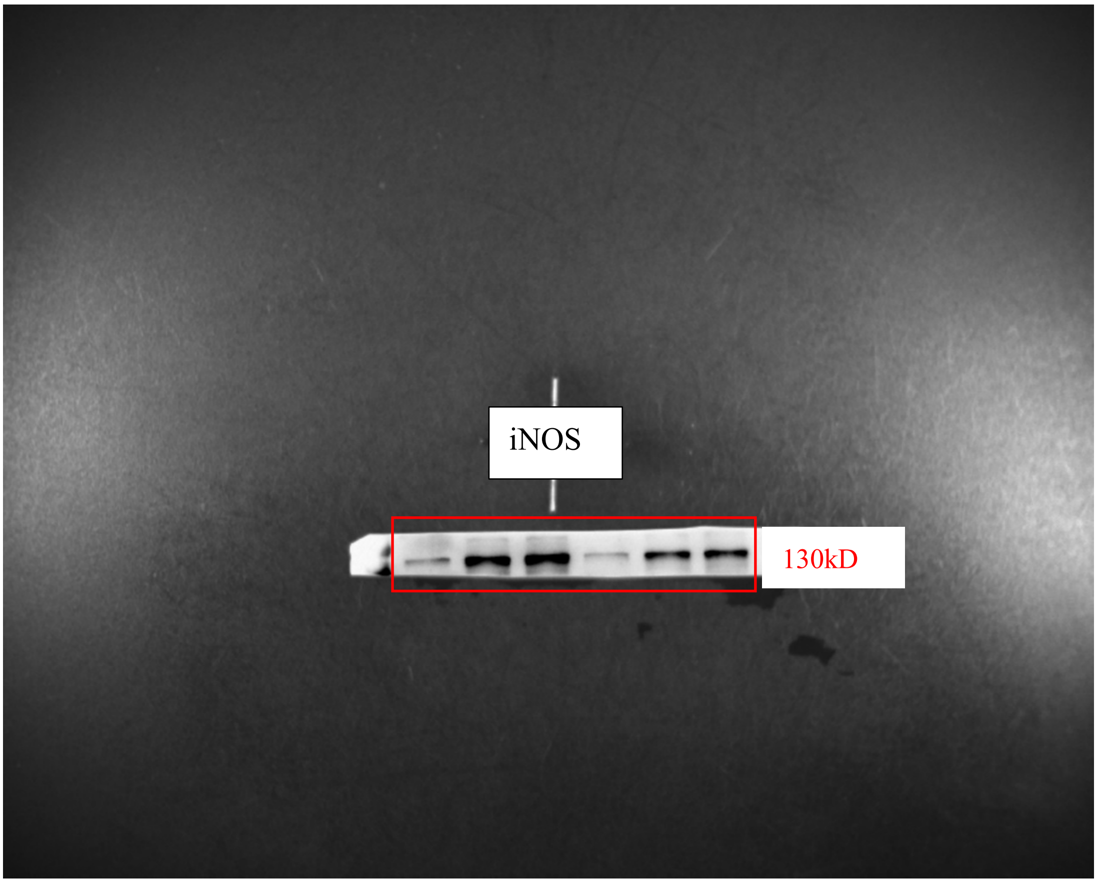

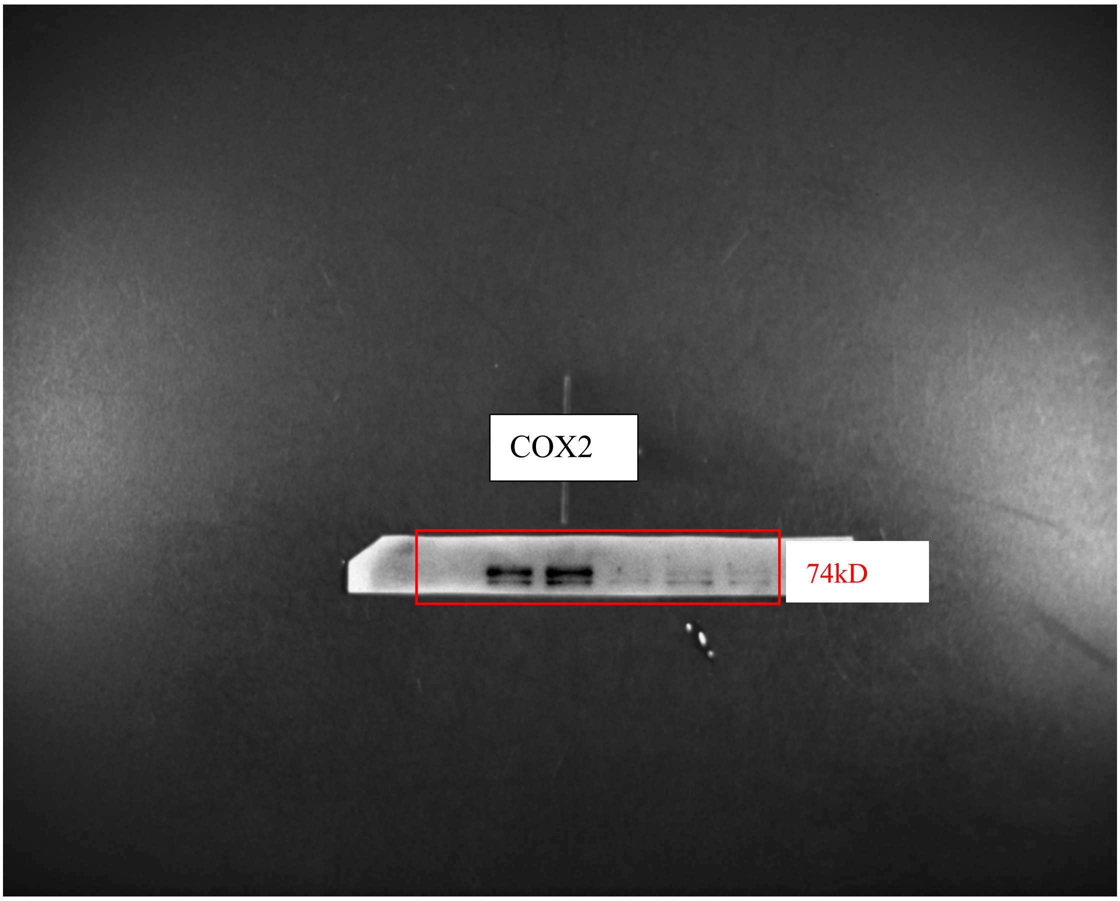
COX2
74kD

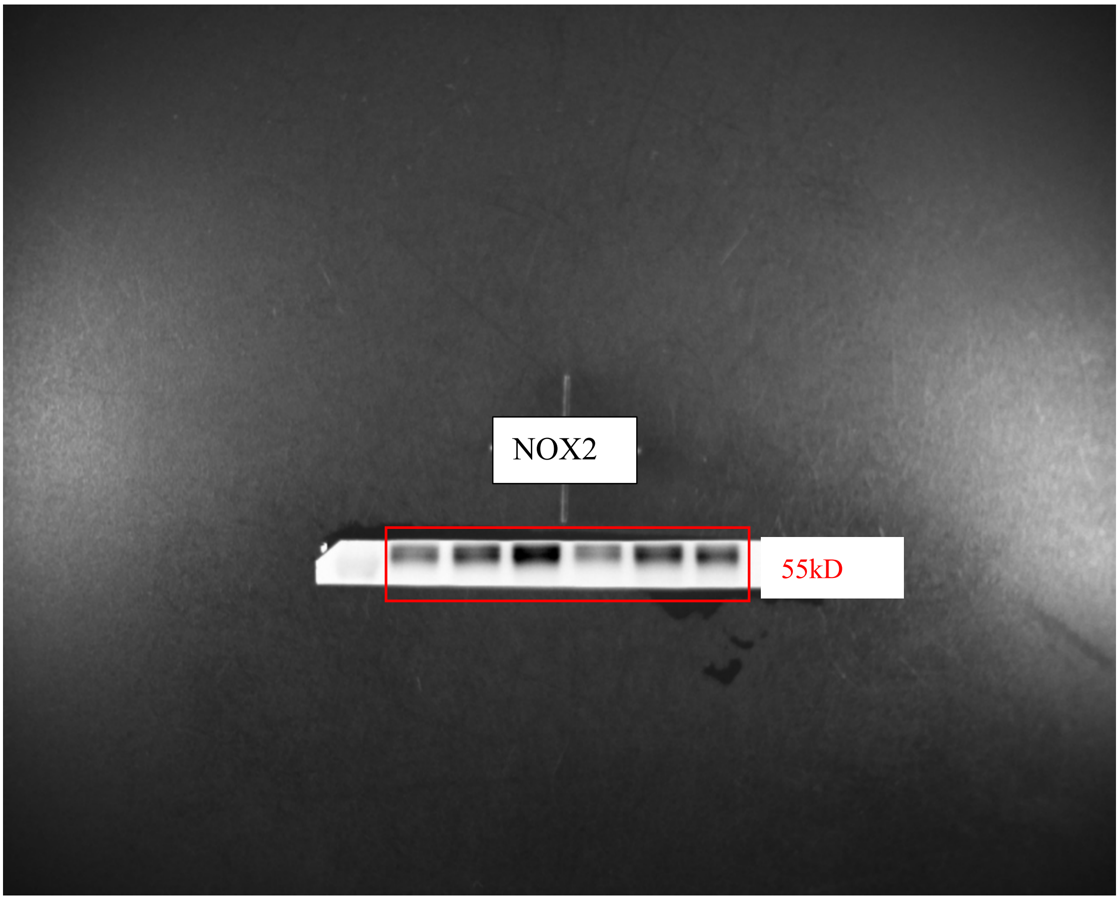
NOX2
55kD

Figure 2D

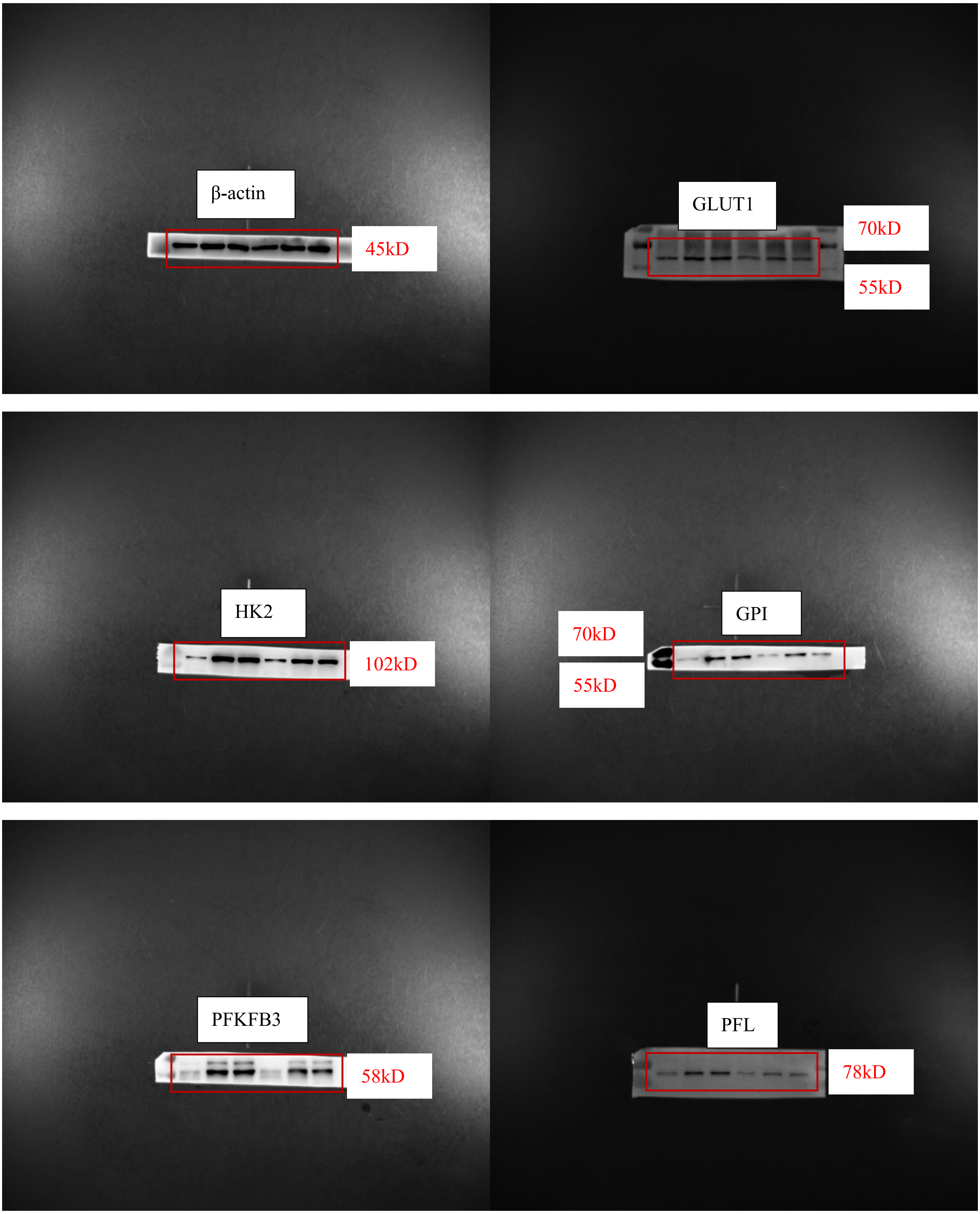
β-actin
45kD
GLUT1
70kD
55kD
HK2
102kD
GPI
70kD
55kD
PFKFB3
58kD
PFL
78kD

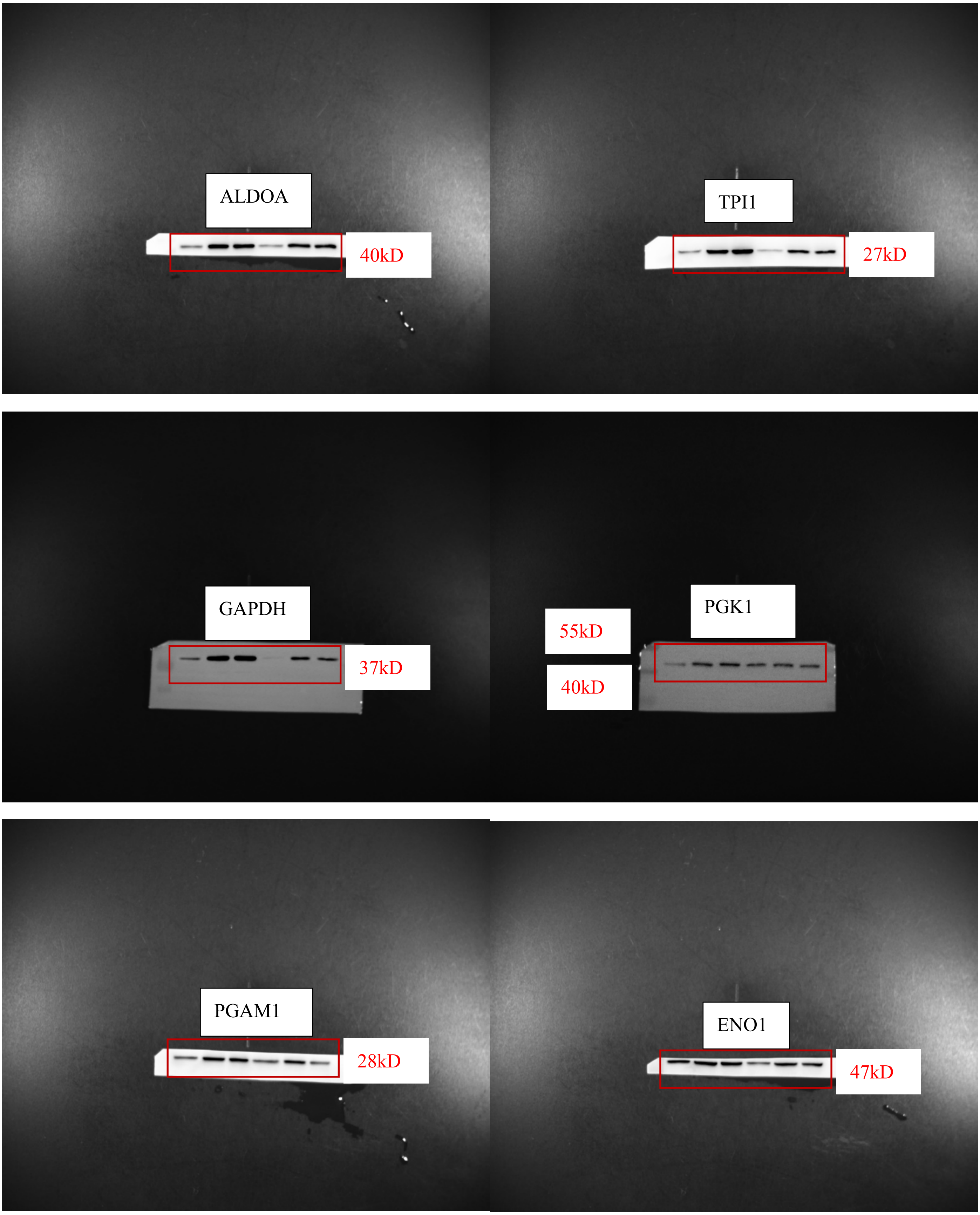
ALDOA
40kD
TPI1
27kD
GAPDH
37kD
PGK1
55kD
40kD
PGAM1
28kD
ENO1
47kD

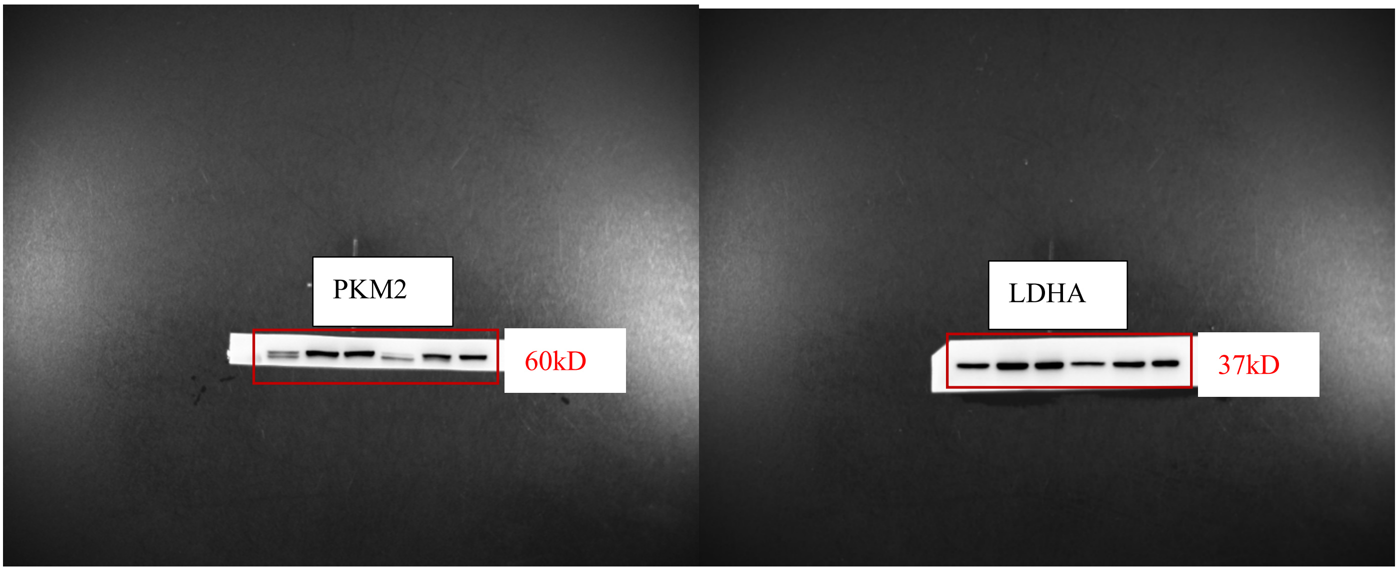

Figure 2H

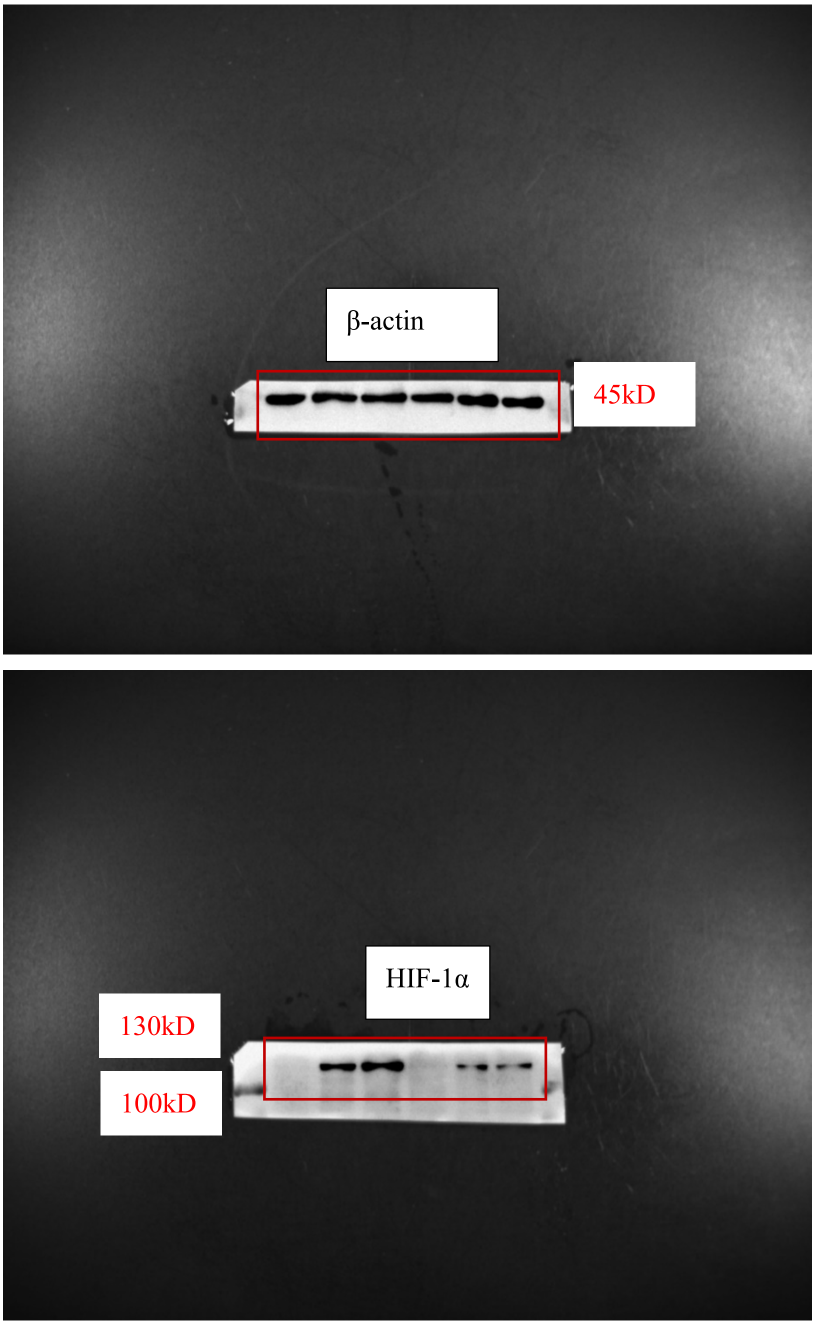

Figure 3C

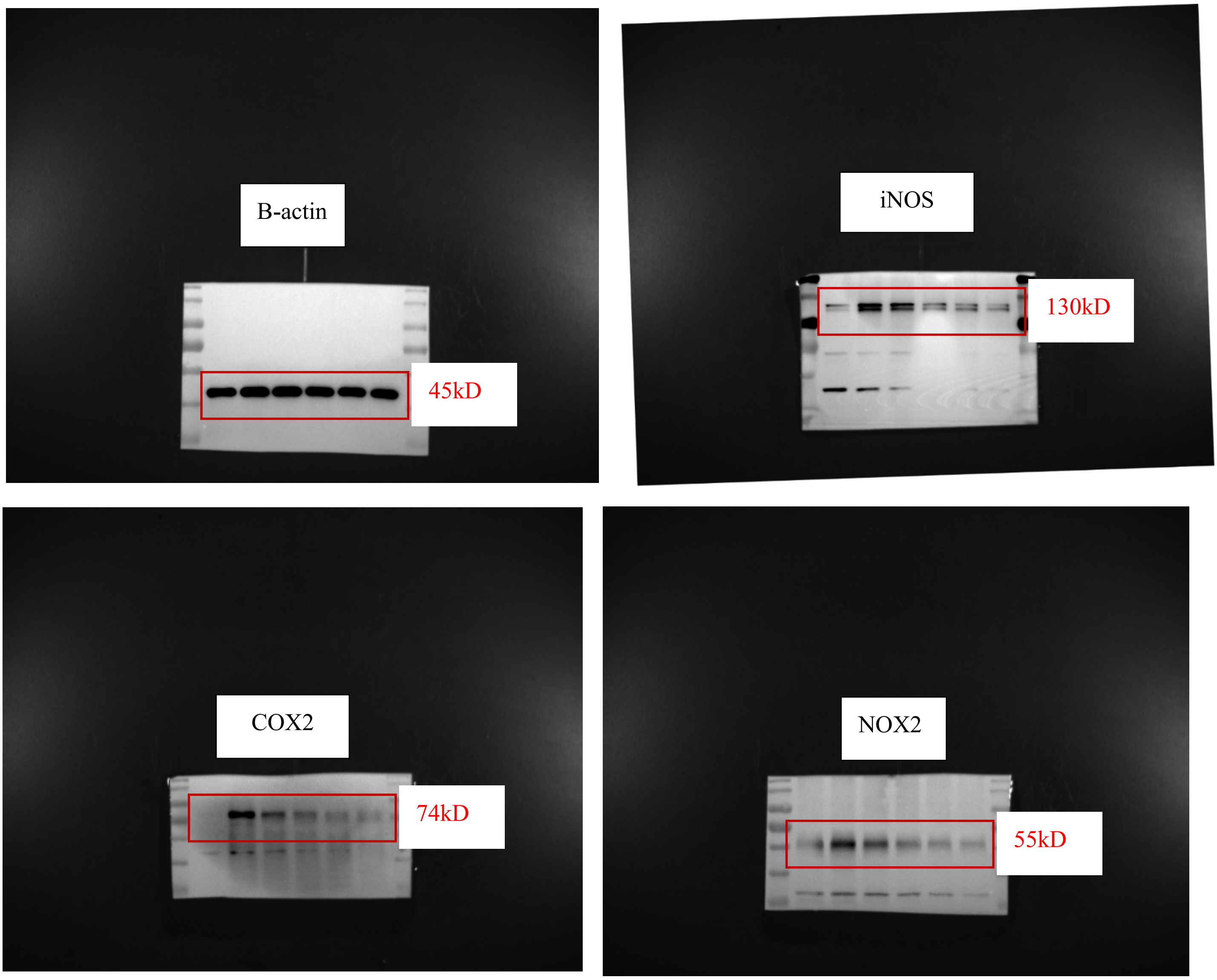

Figure 3E

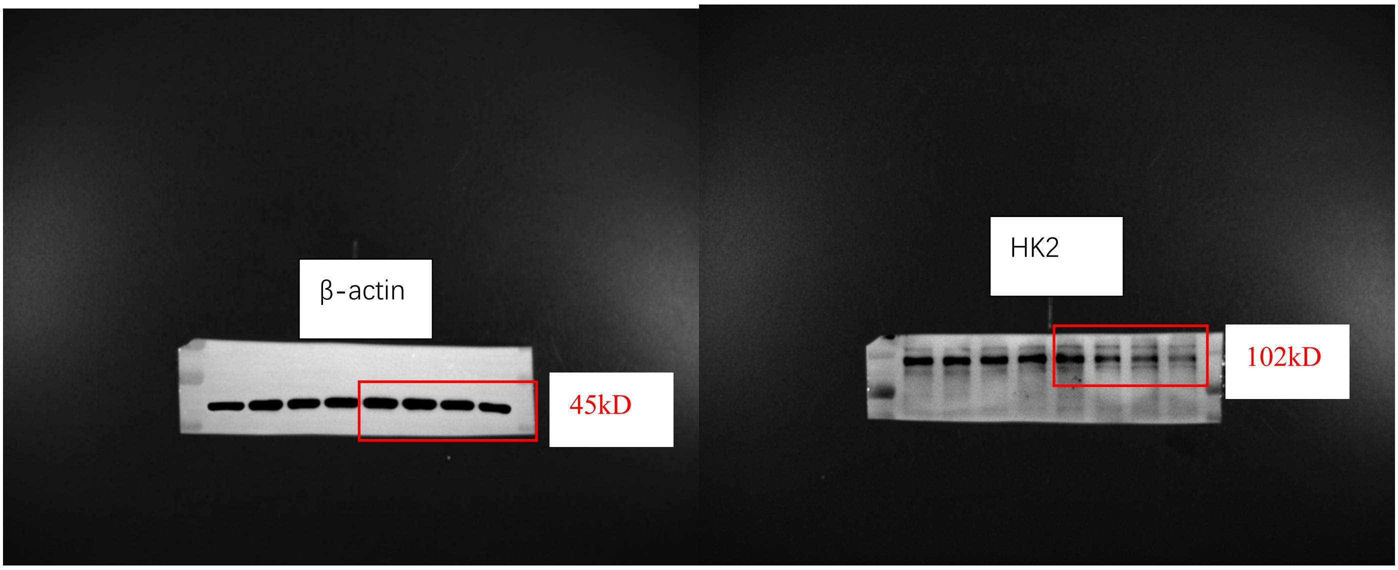

Figure 3G

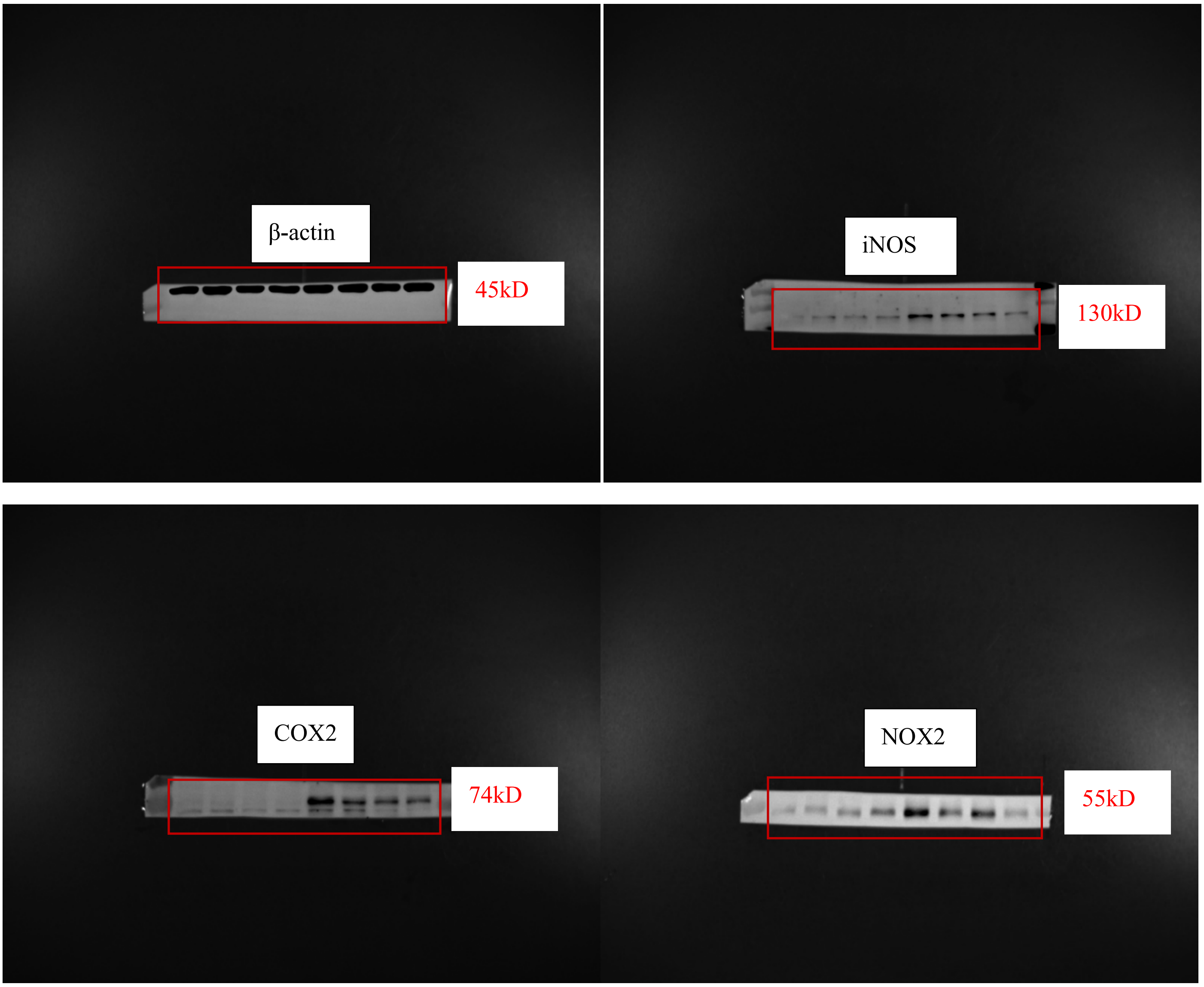

Figure 4A

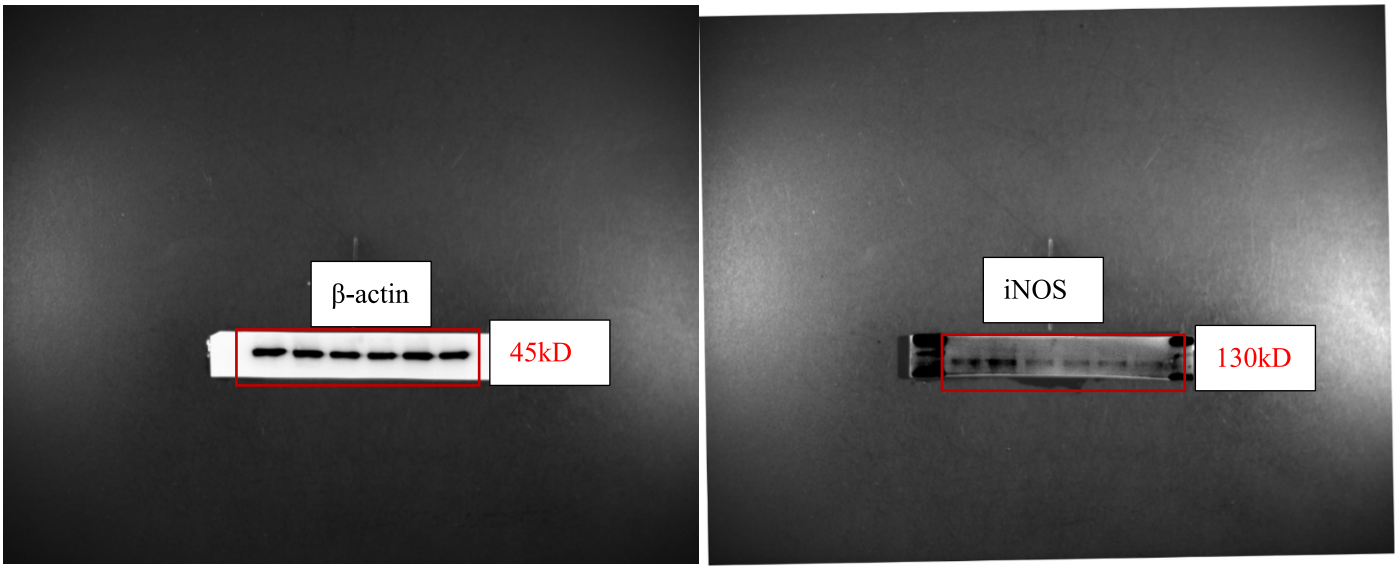

Figure 4B

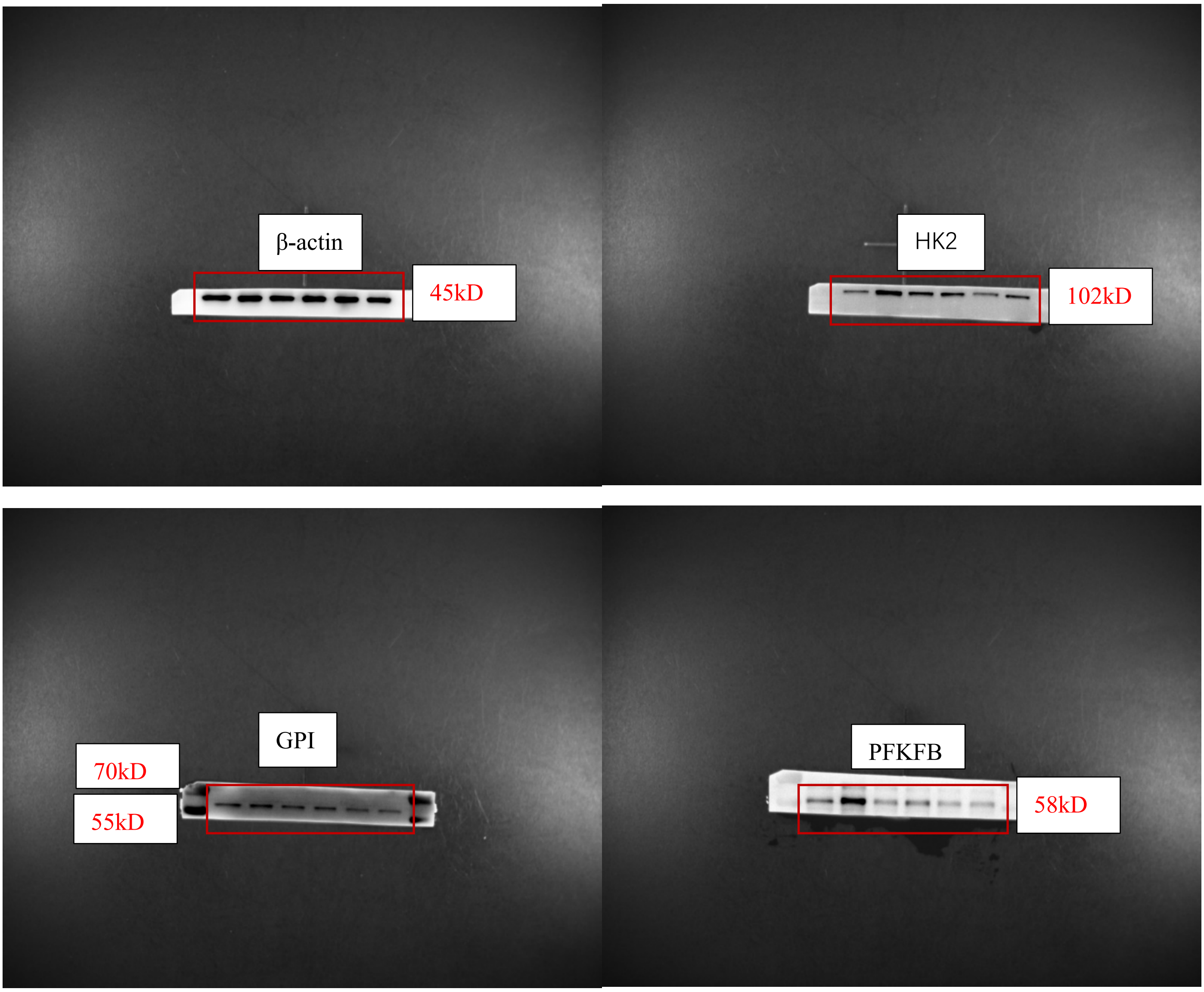

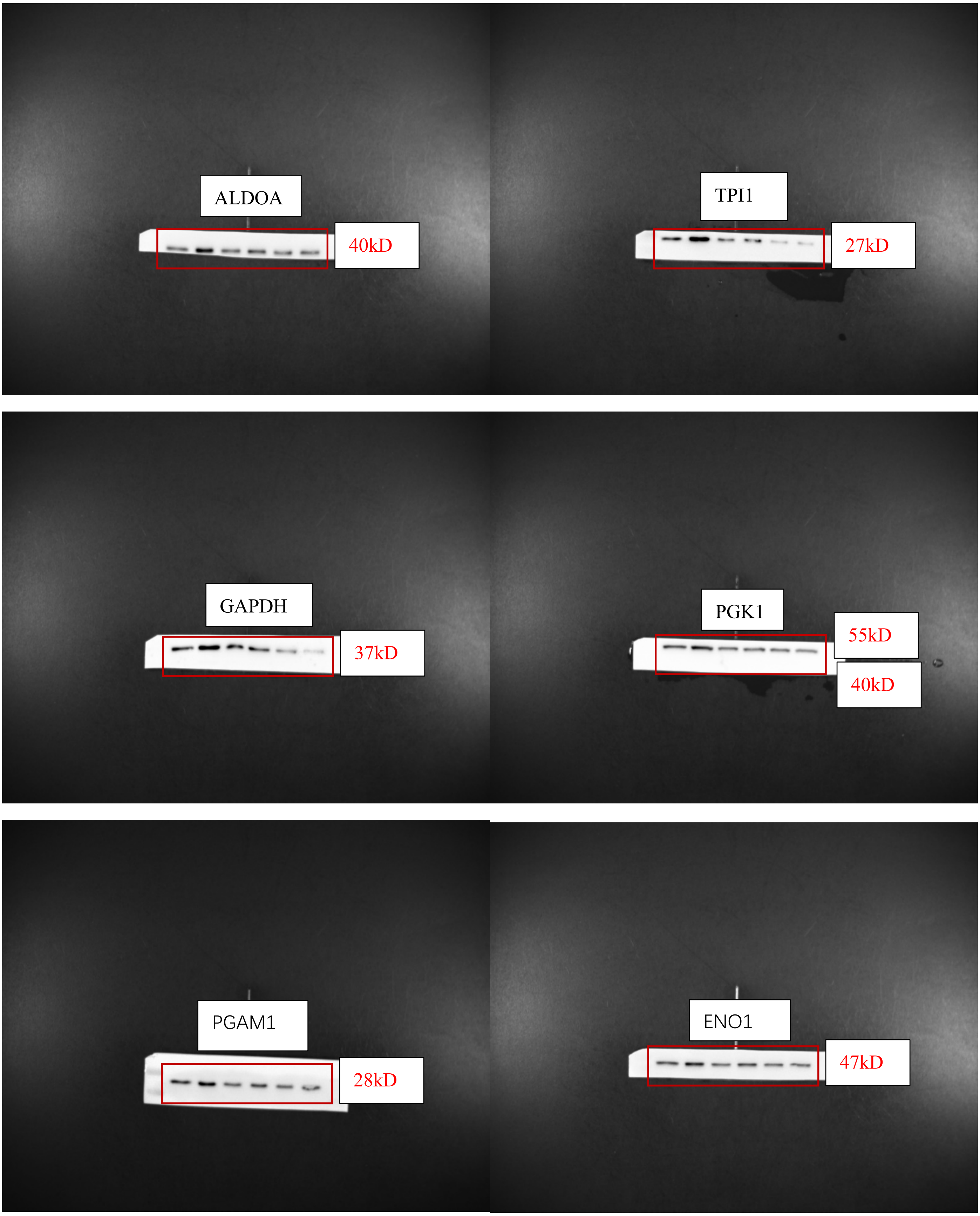
ALDOA
40kD
TPI1
27kD
GAPDH
37kD
PGK1
55kD
40kD
PGAM1
28kD
ENO1
47kD

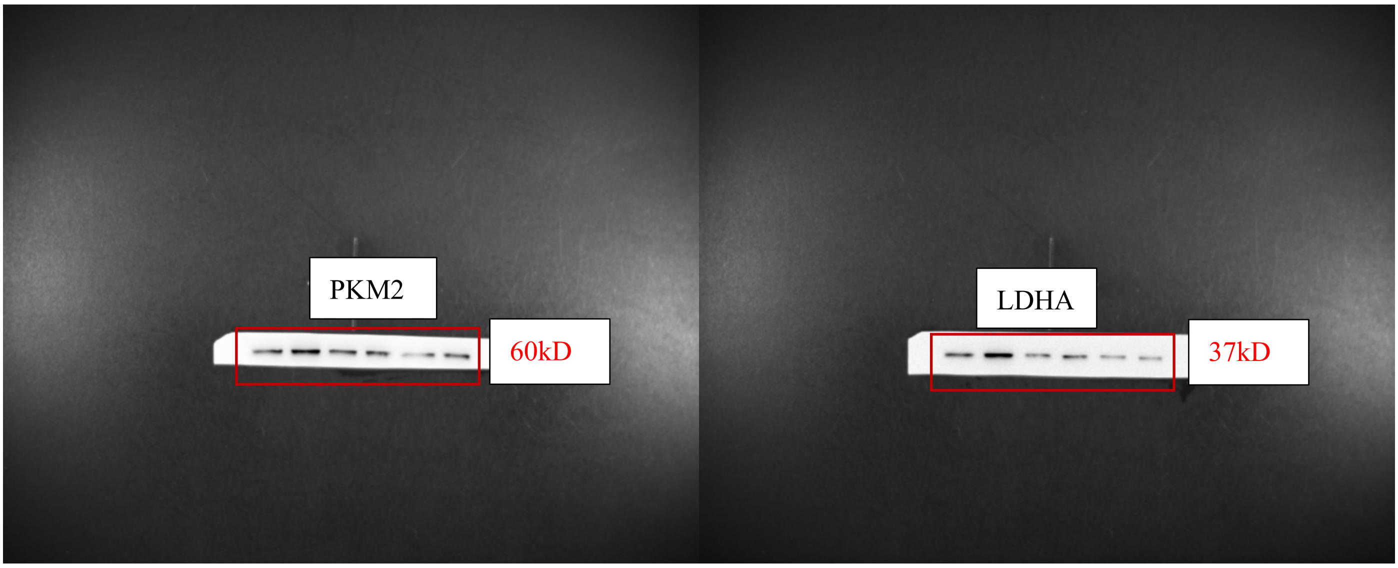

Figure 4E

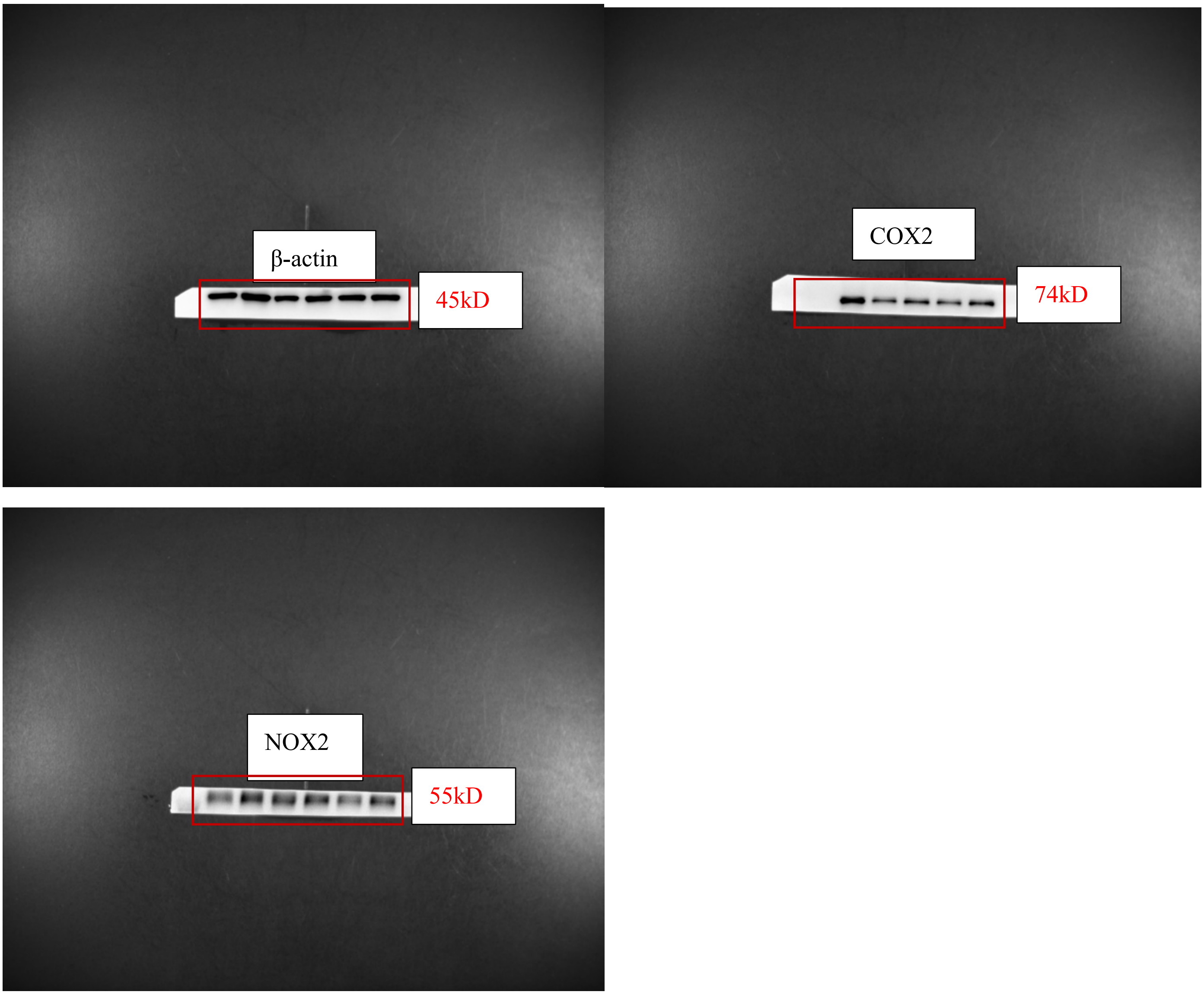

Figure 4G

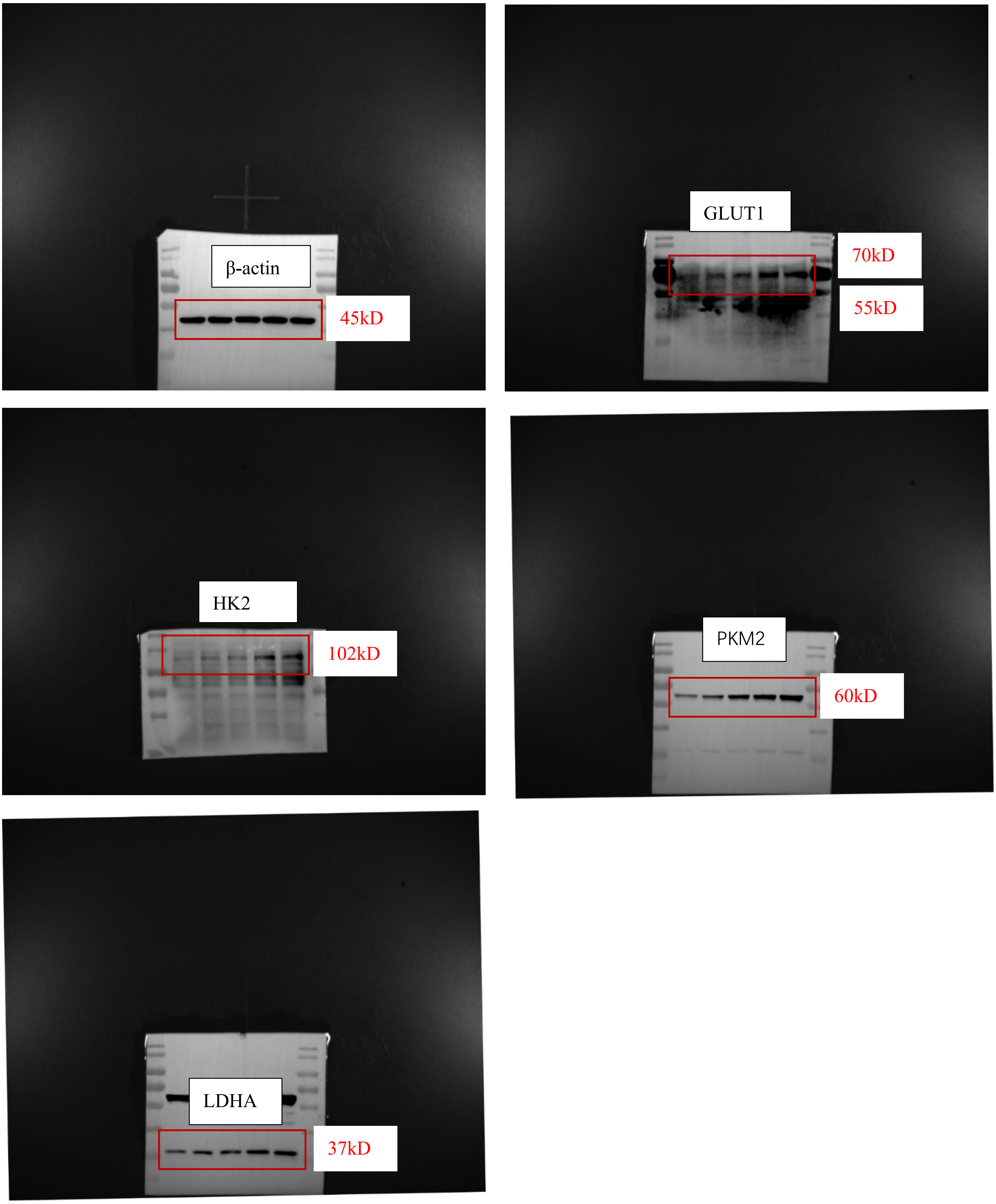

Figure 5A

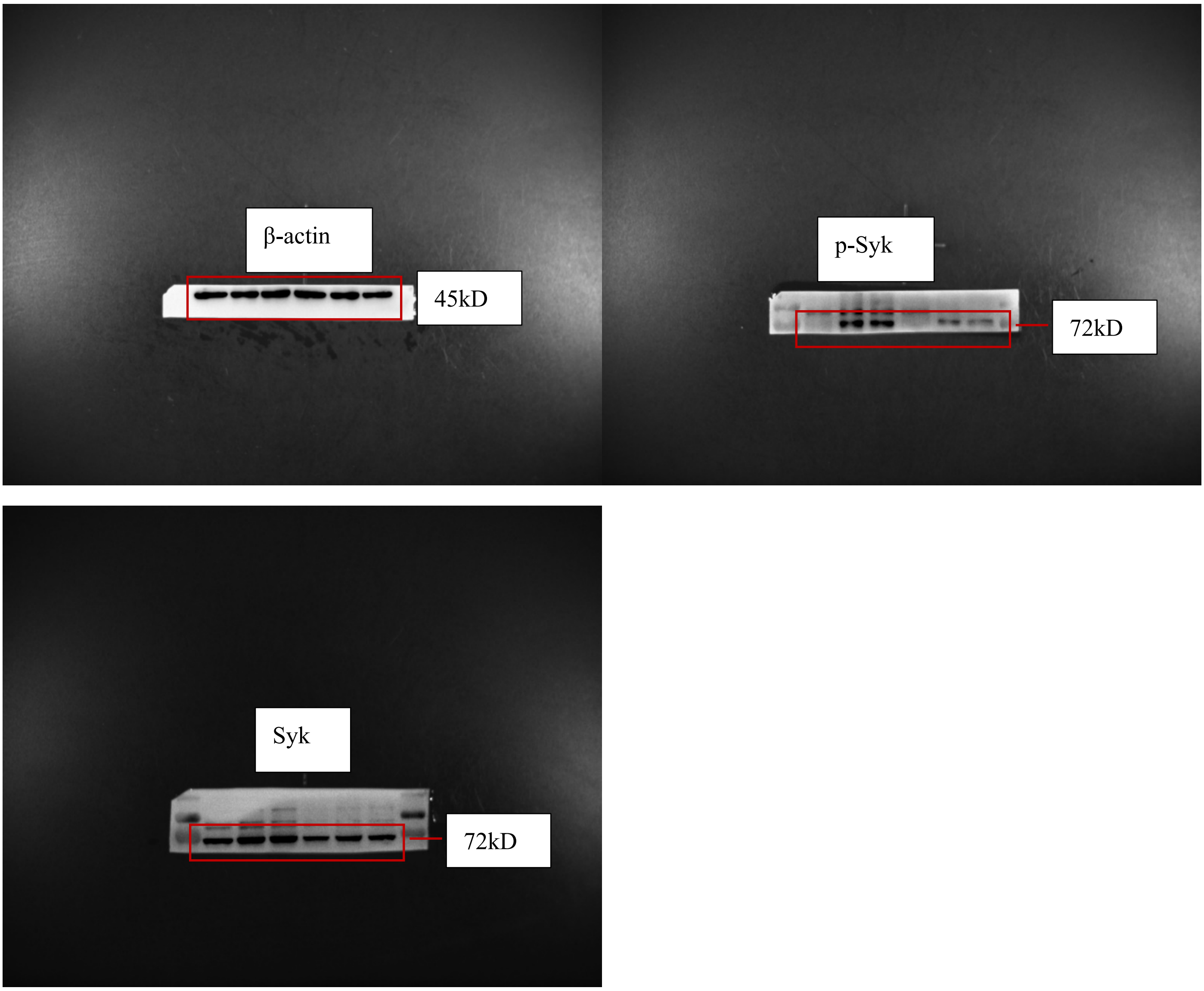

Figure 5E

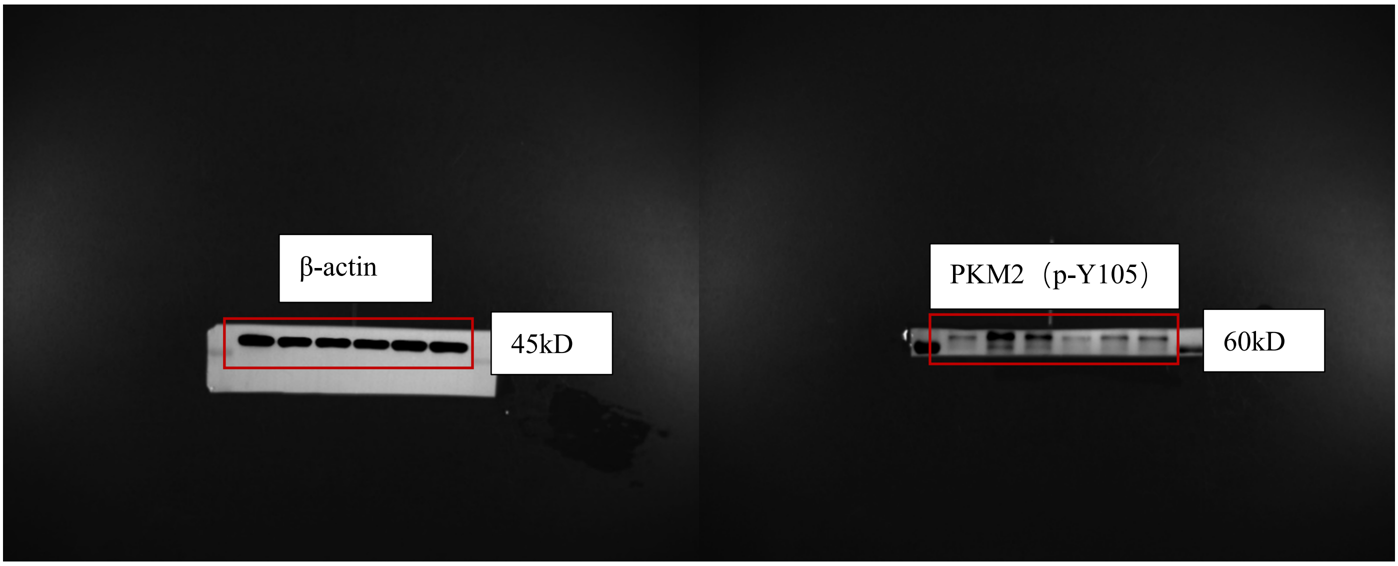

Figure 5F

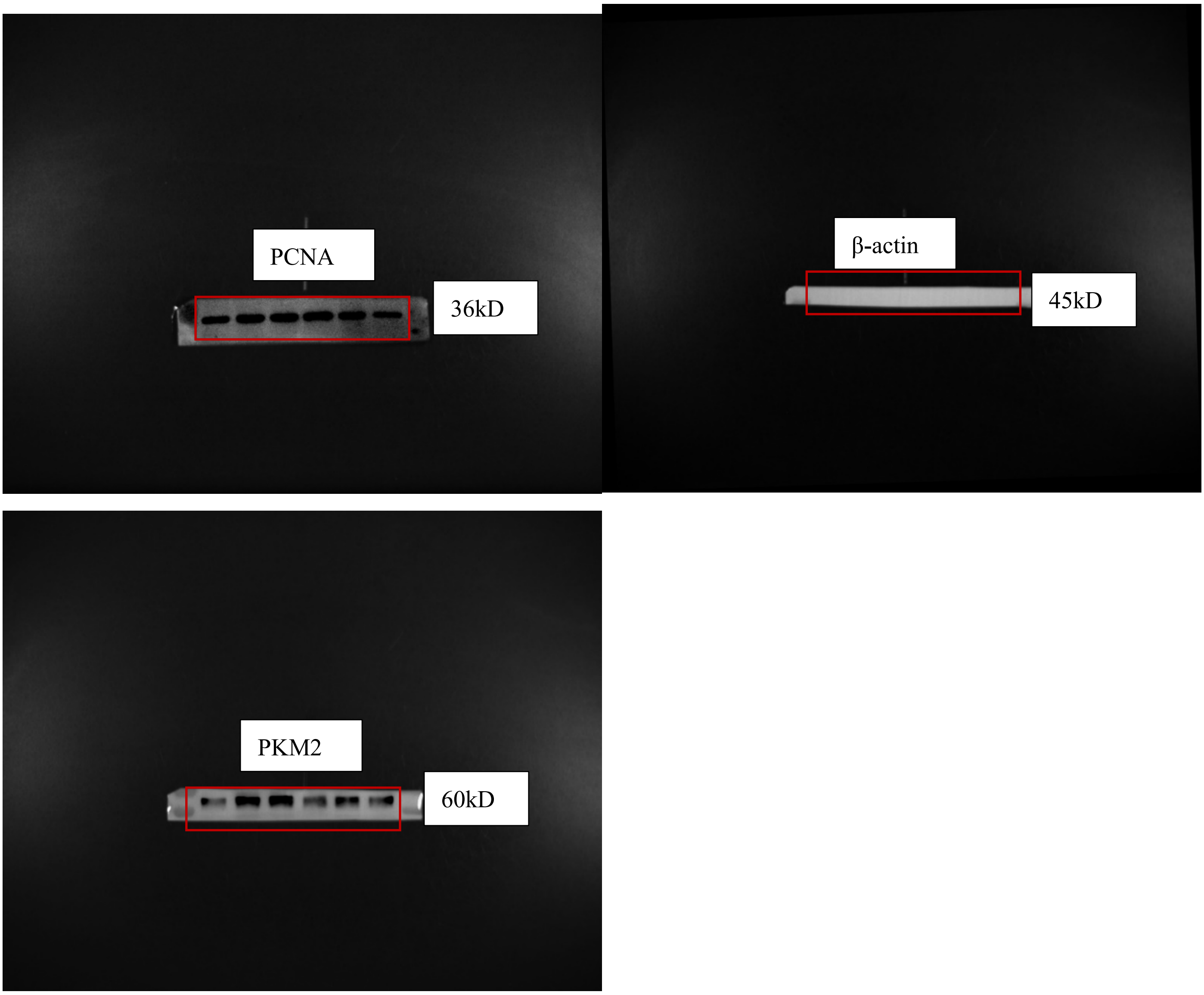

Figure 5G

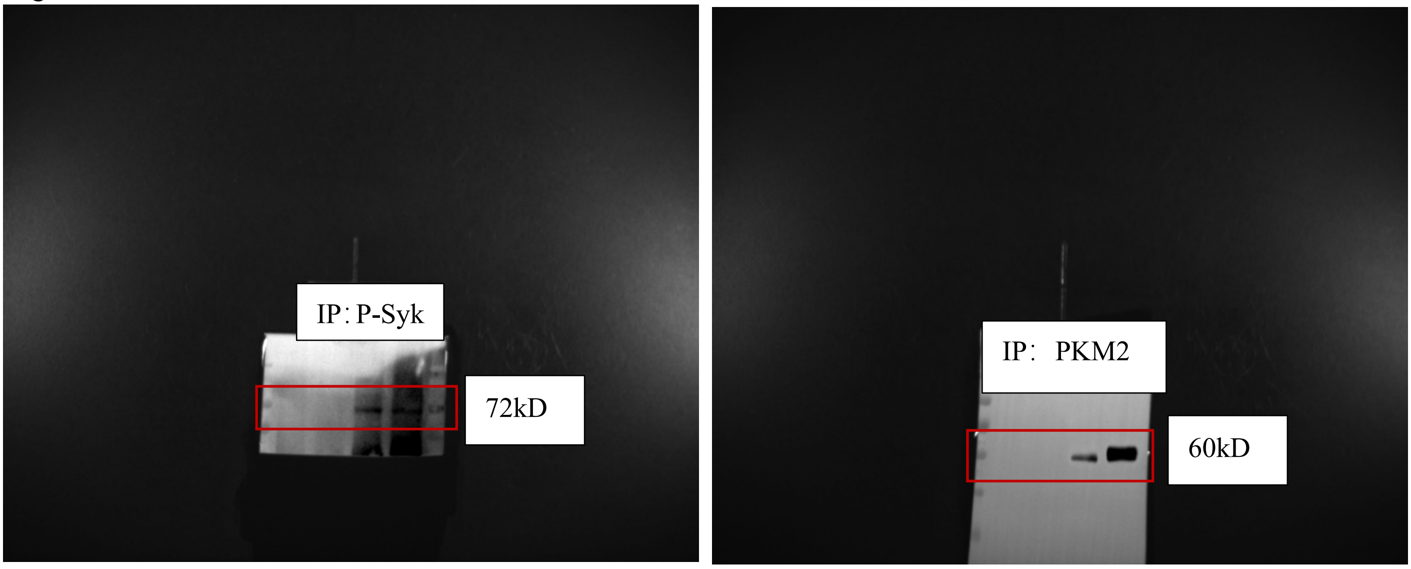

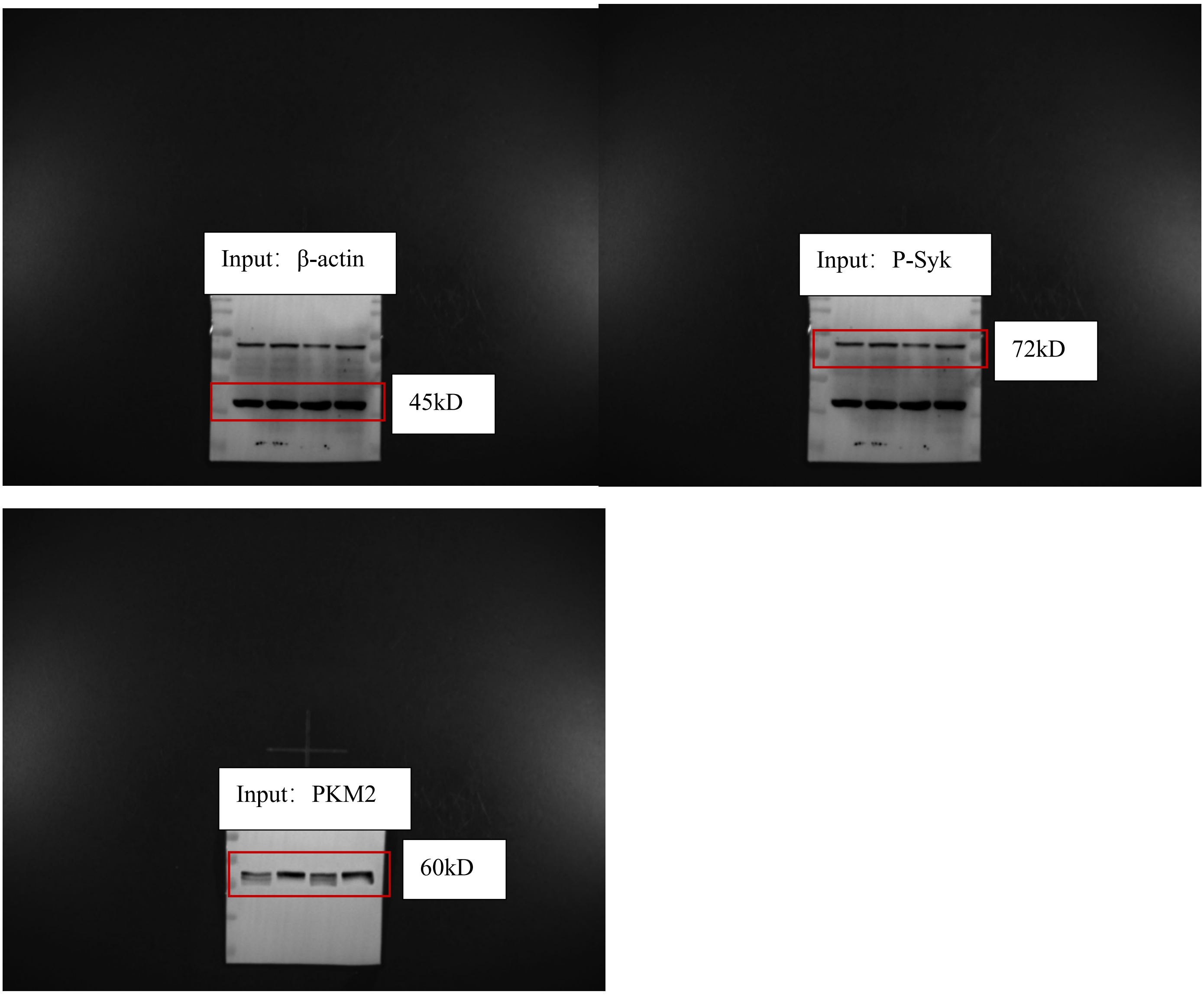

Input: β-actin
45kD
Input: P-Syk
72kD
Input: PKM2
60kD

Figure 5H

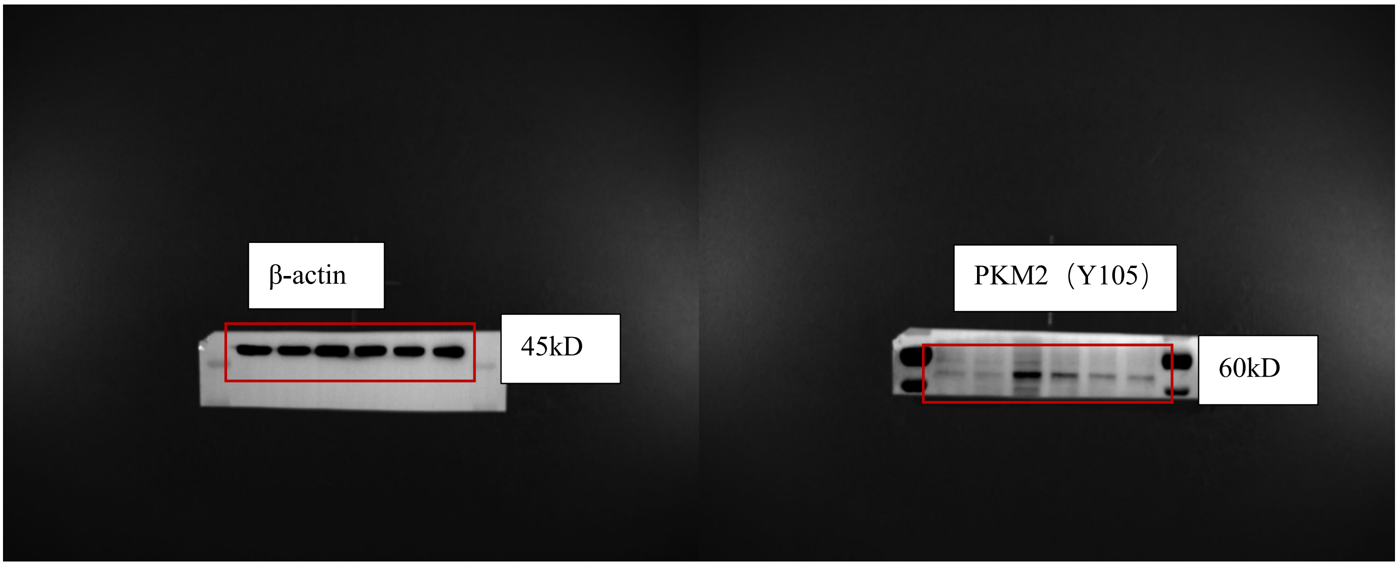

β-actin
45kD
PKM2（Y105）
60kD

Figure 5I

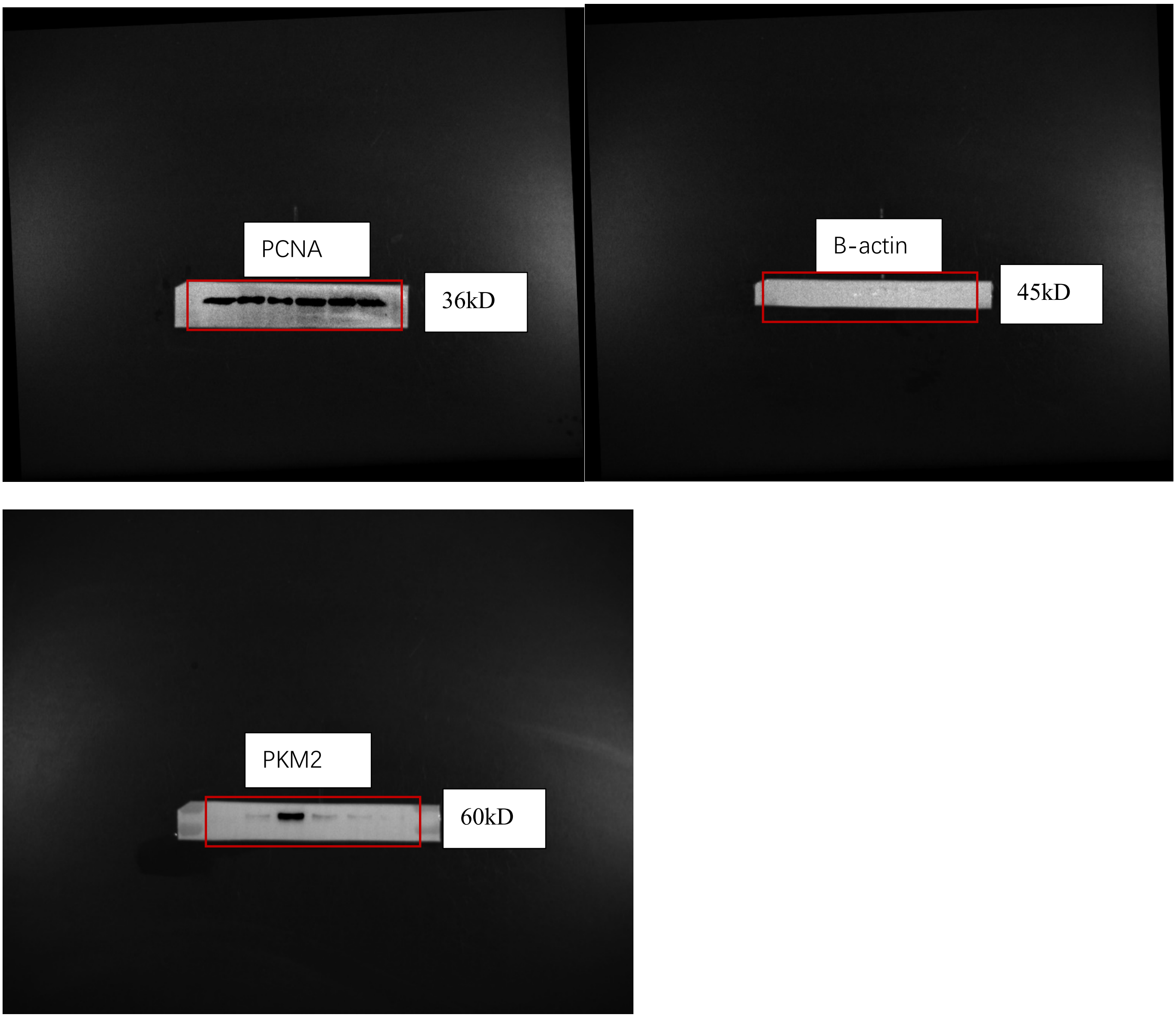

Figure 6A

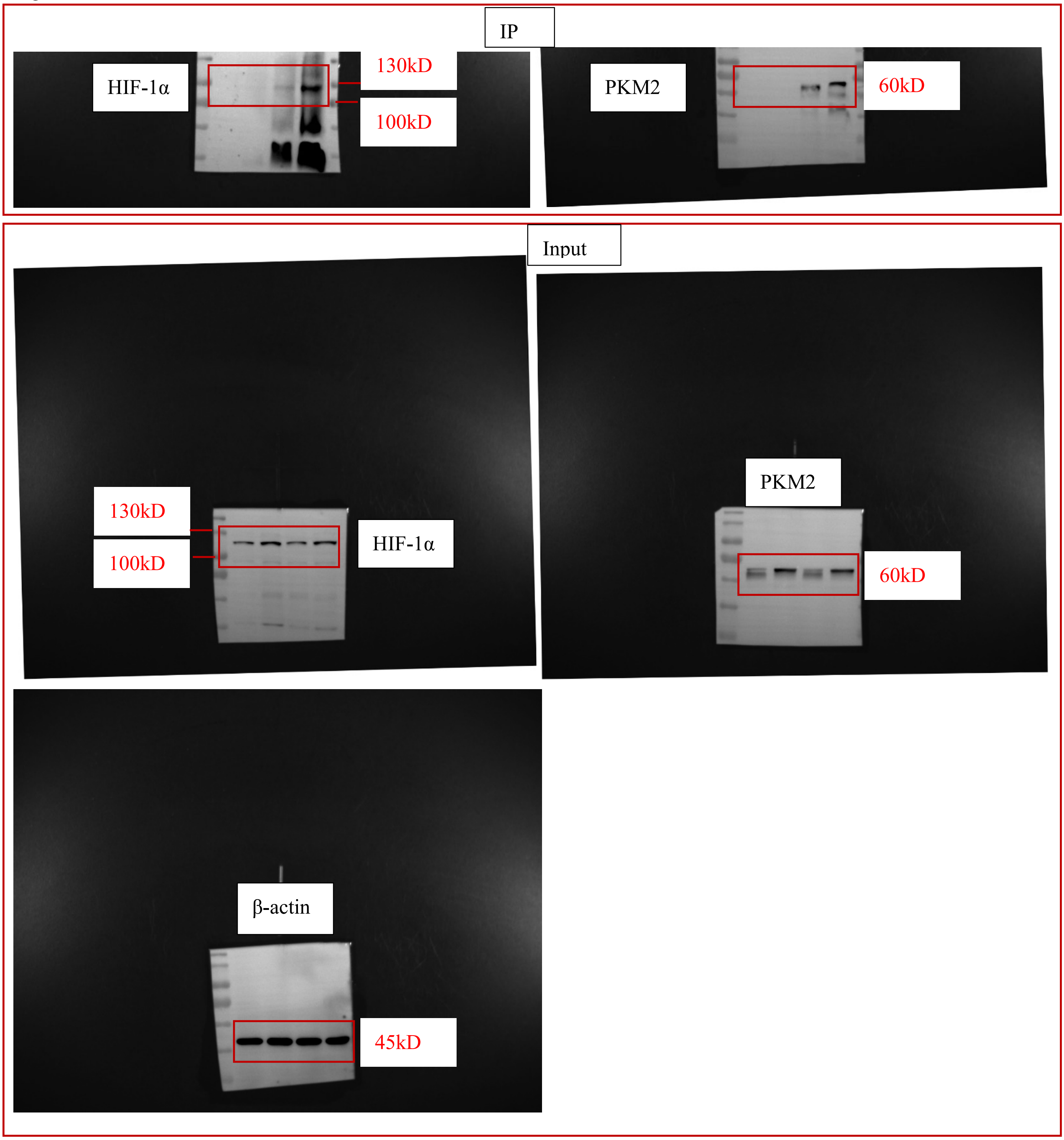
IP
HIF-1α
130kD
100kD
PKM2
60kD
Input
130kD
100kD
HIF-1α
PKM2
60kD
β-actin
45kD

Figure 6B

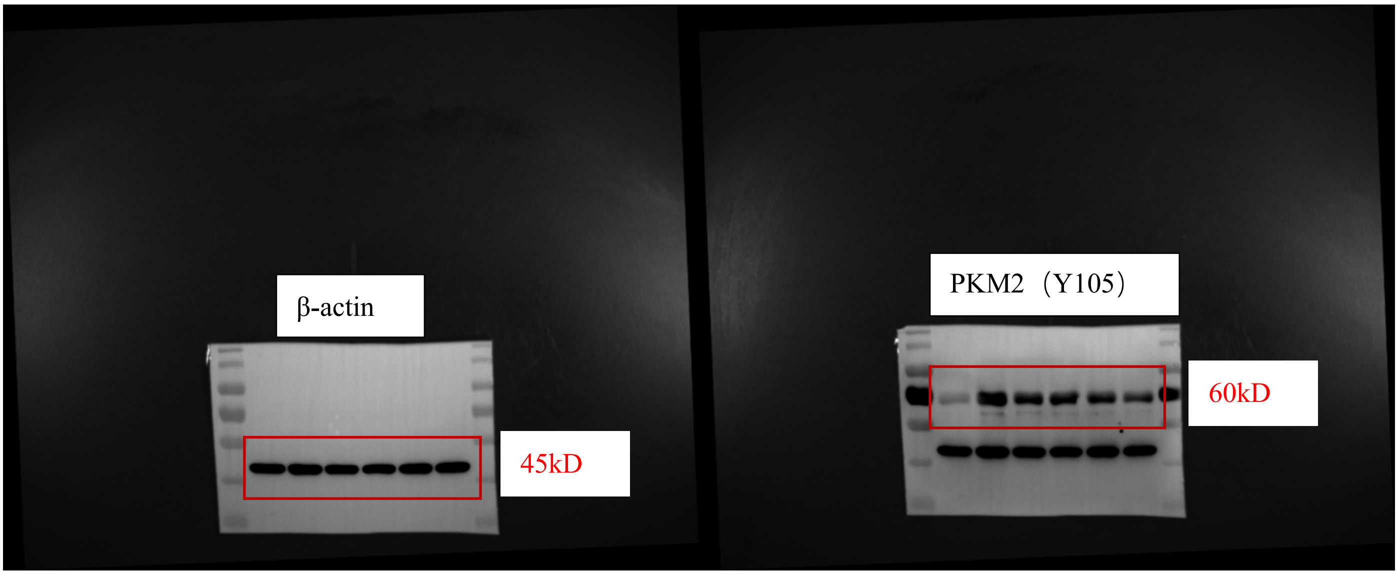

Figure 6C

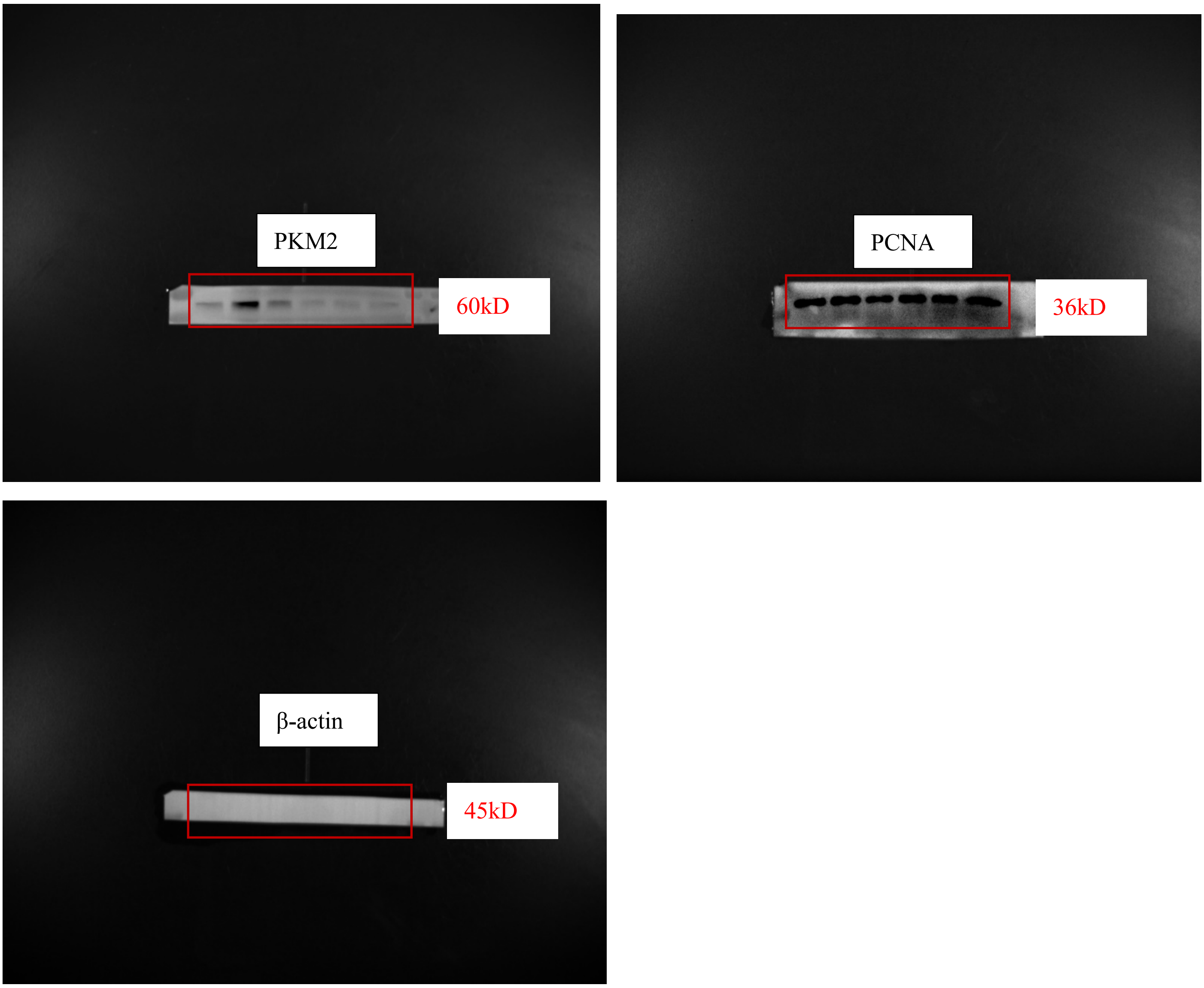

Figure 6E

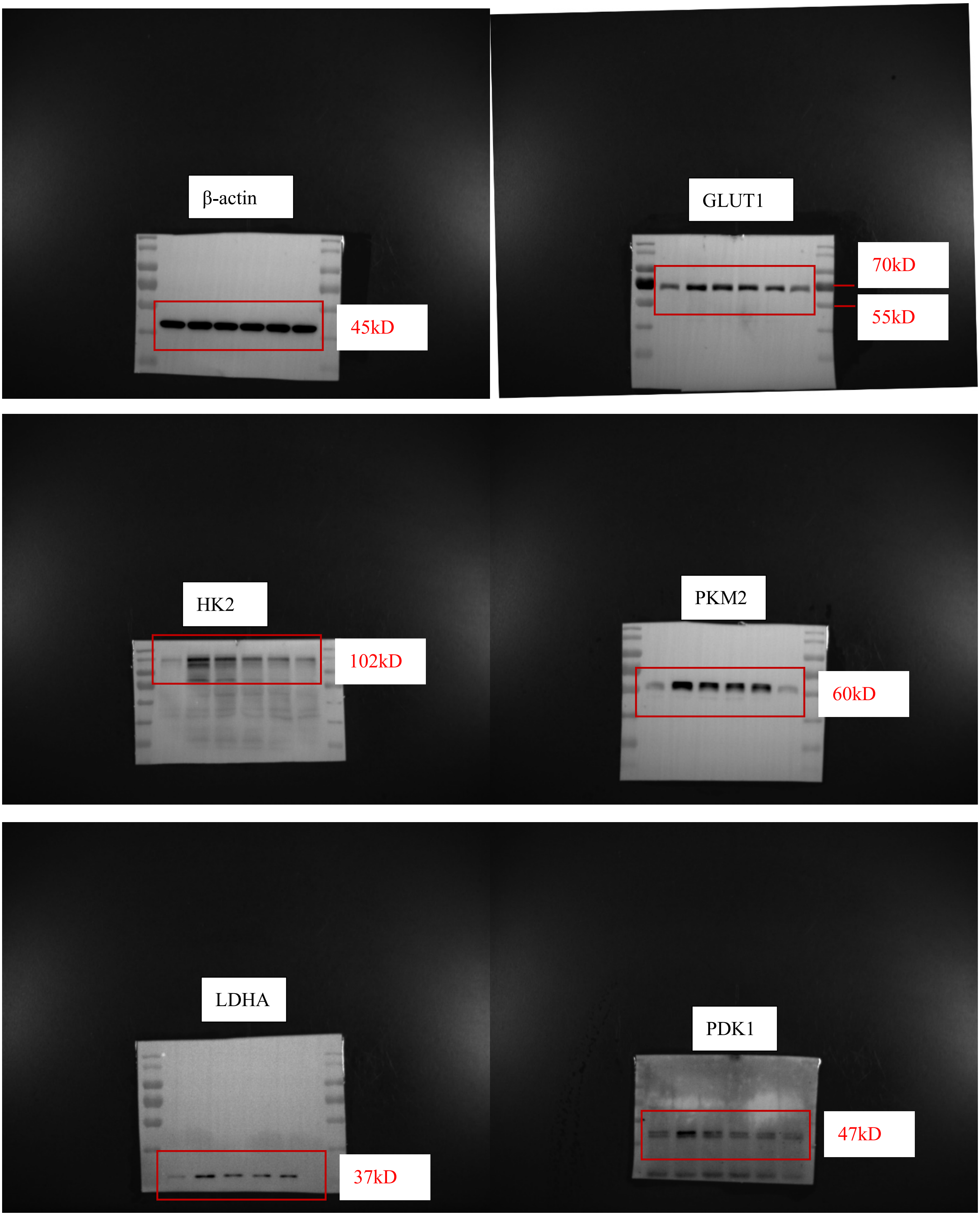
β-actin
45kD
GLUT1
70kD
55kD
HK2
102kD
PKM2
60kD
LDHA
37kD
PDK1
47kD

Figure 6H

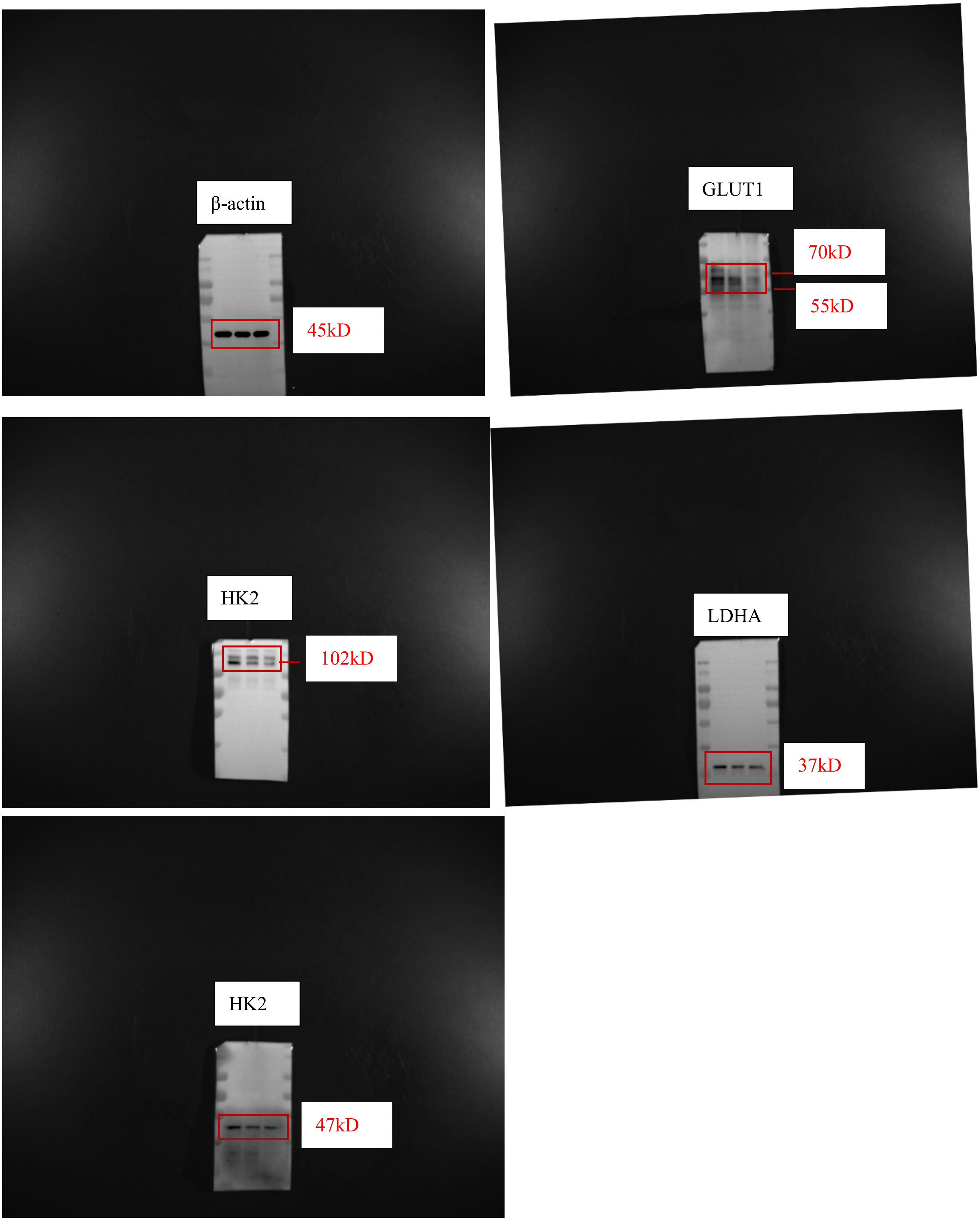

Figure S1C

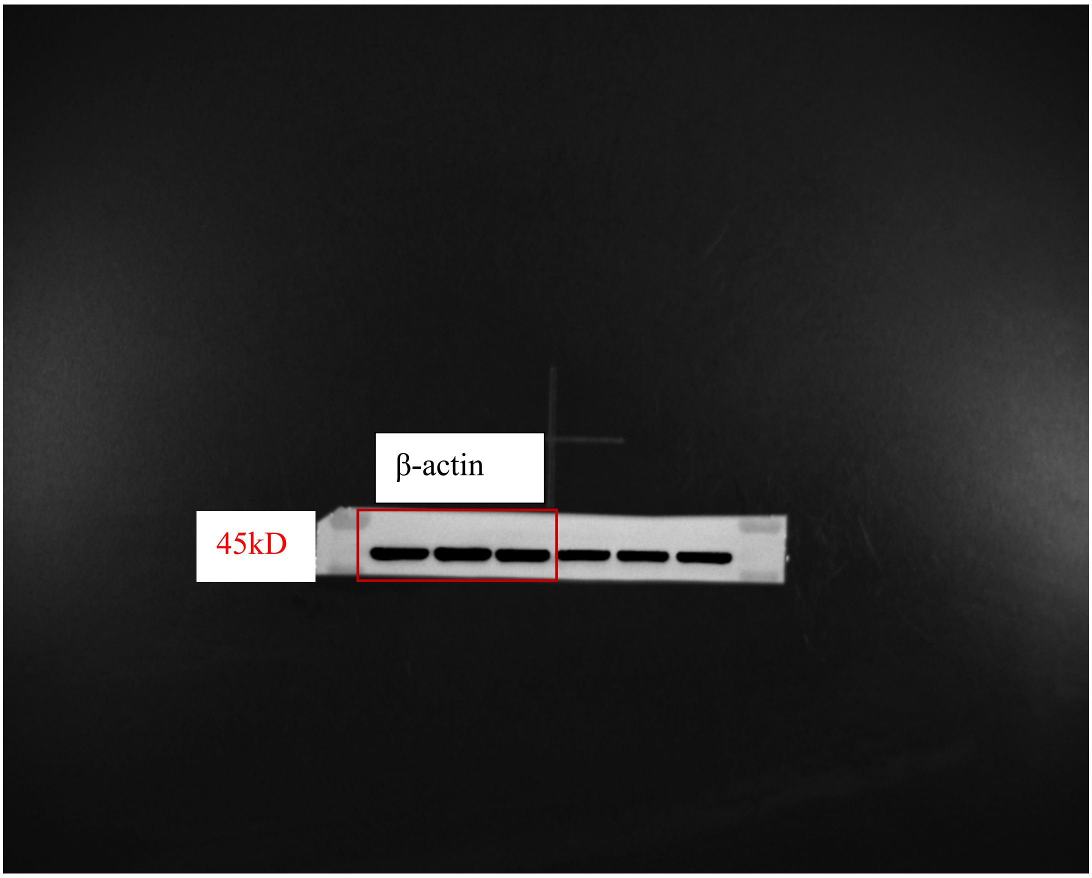

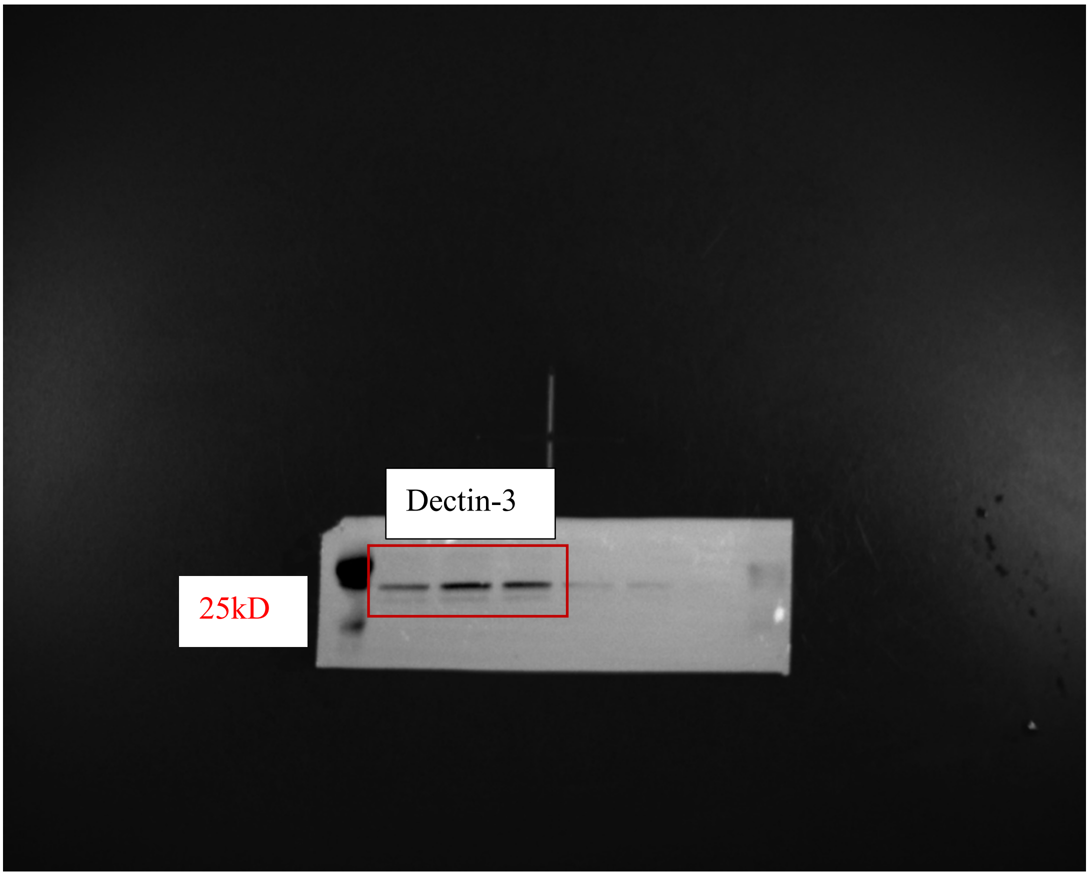

Figure S1E

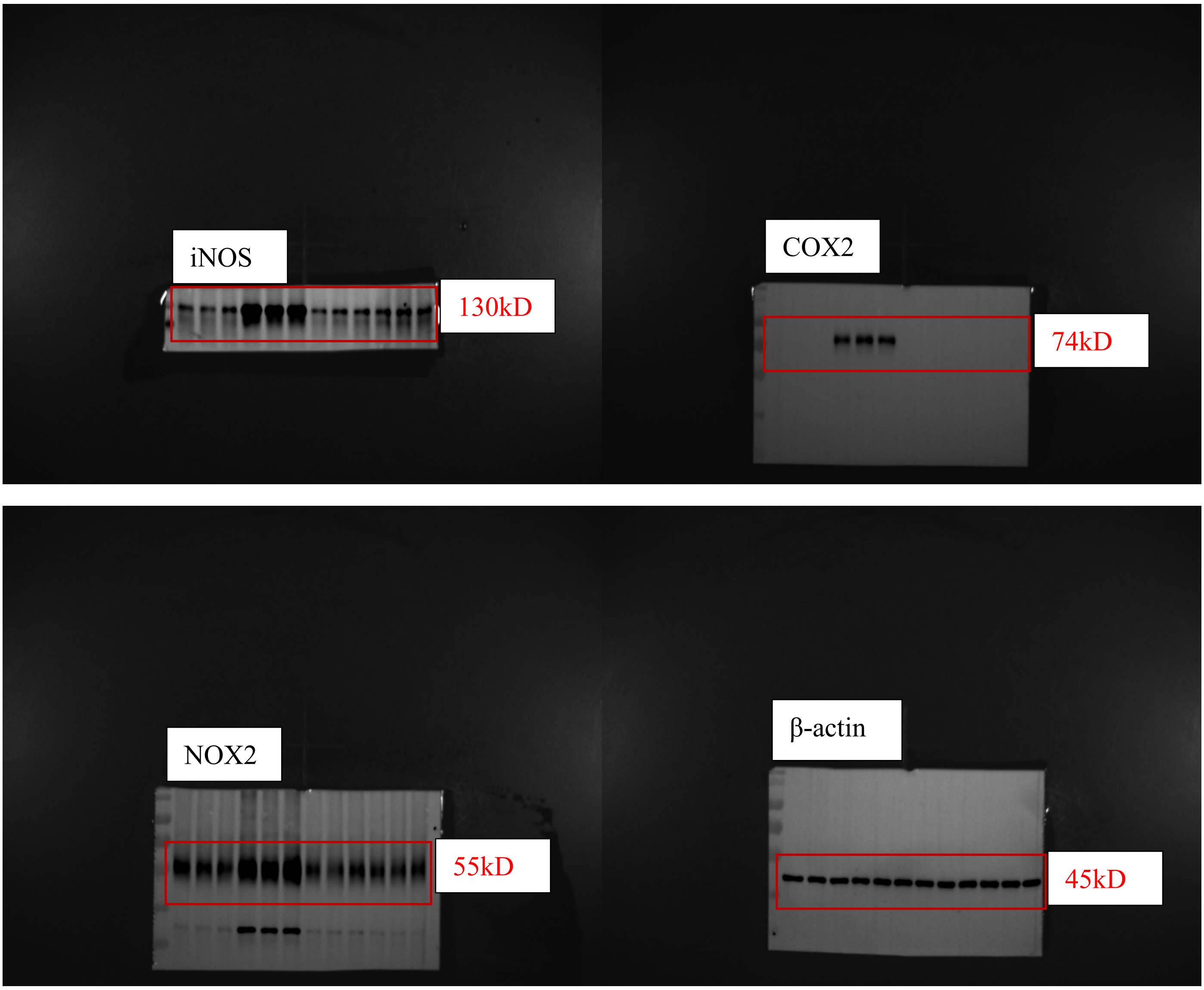

Figure S3A

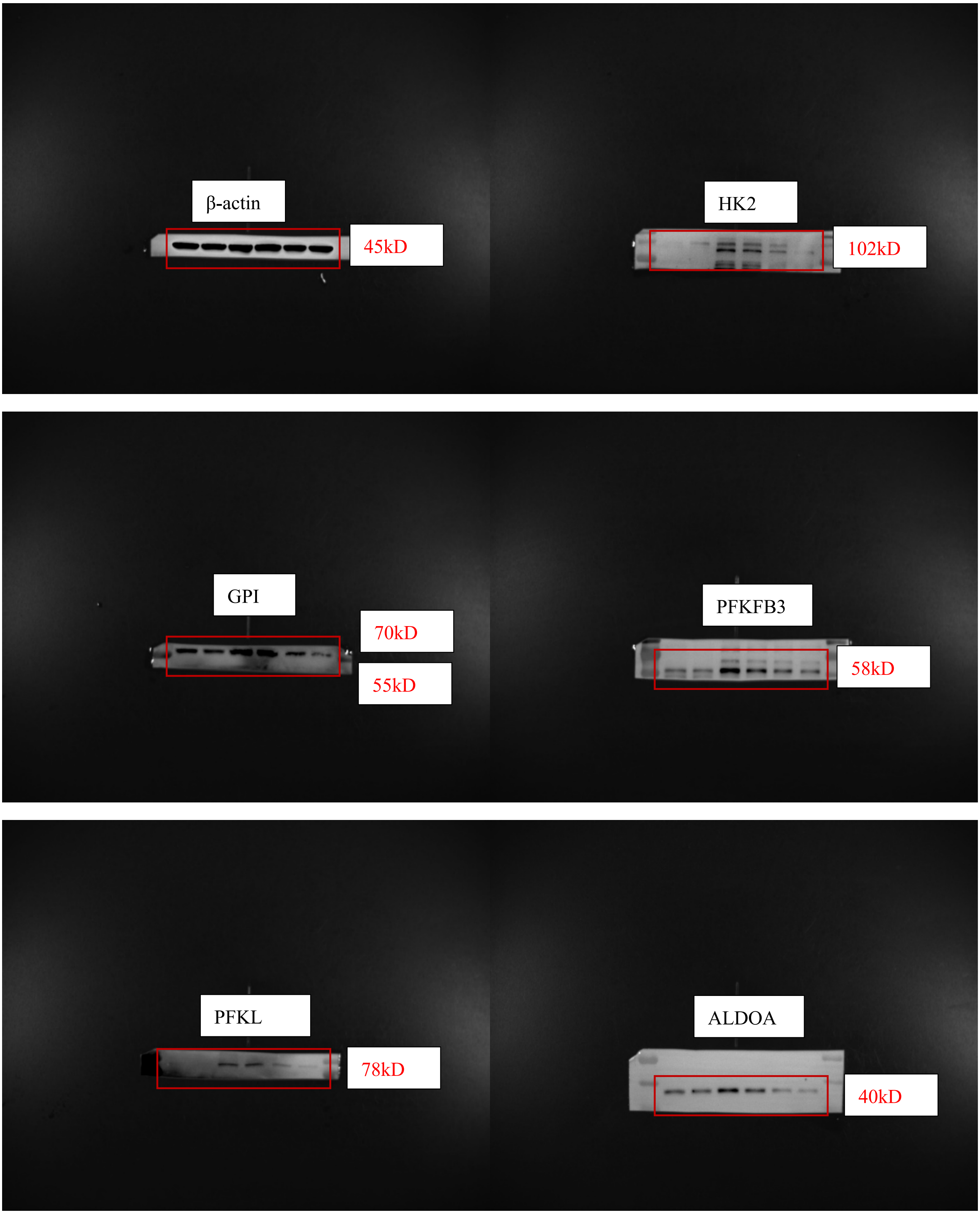

β-actin
45kD
HK2
102kD
GPI
70kD
55kD
PFKFB3
58kD
PFKL
78kD
ALDOA
40kD

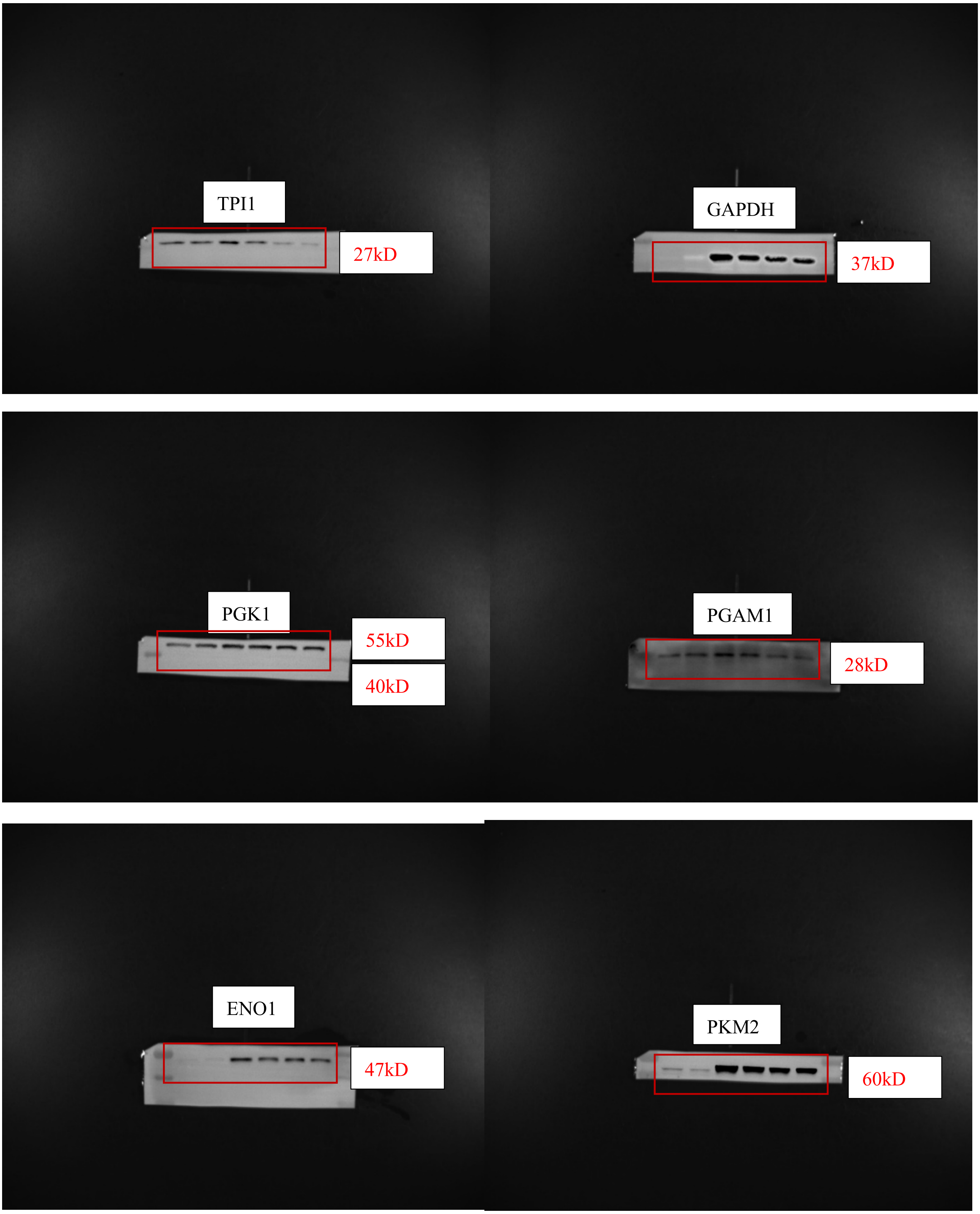
TPI1
27kD
GAPDH
37kD
PGK1
55kD
40kD
PGAM1
28kD
ENO1
47kD
PKM2
60kD

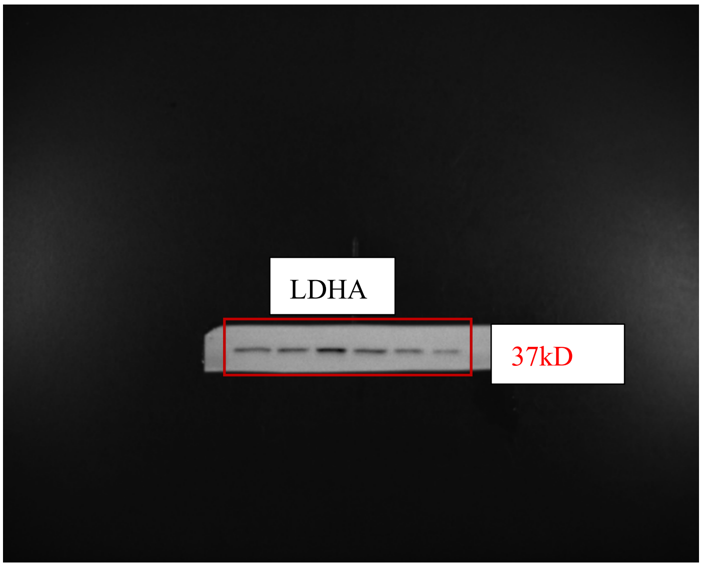

Figure S3B

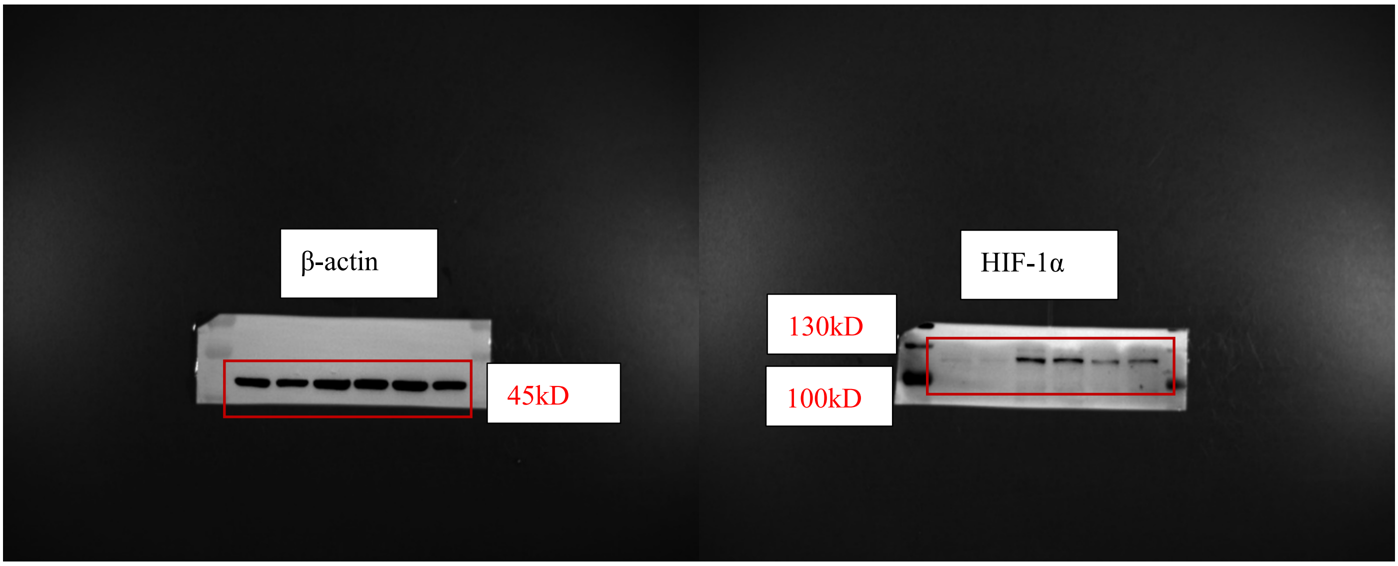

Figure S3D

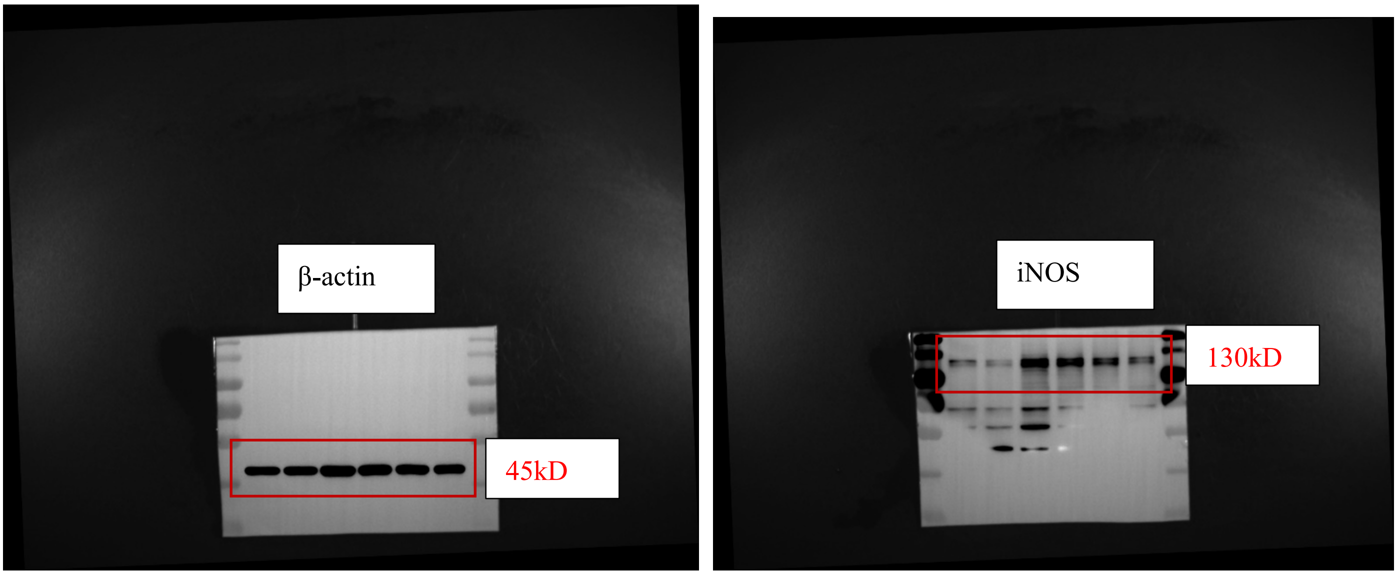

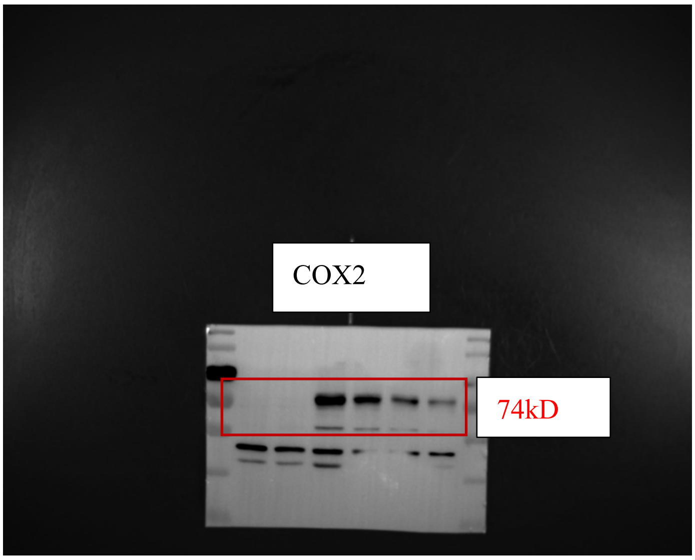
COX2
74kD

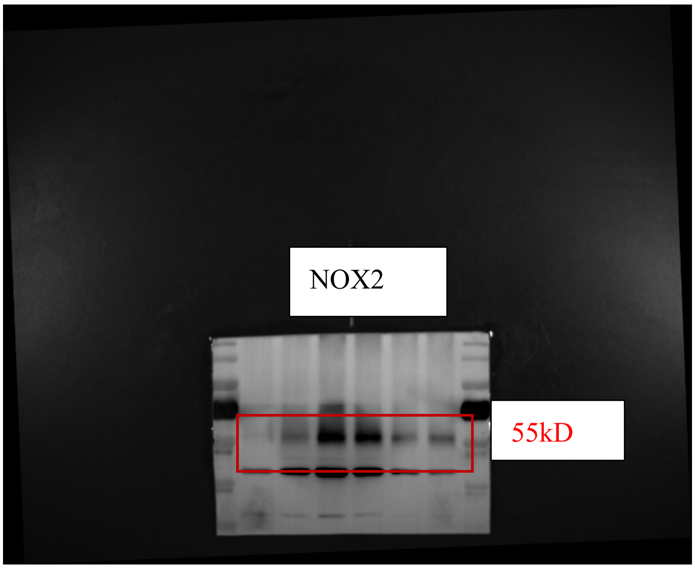
NOX2
55kD

Figure S4B

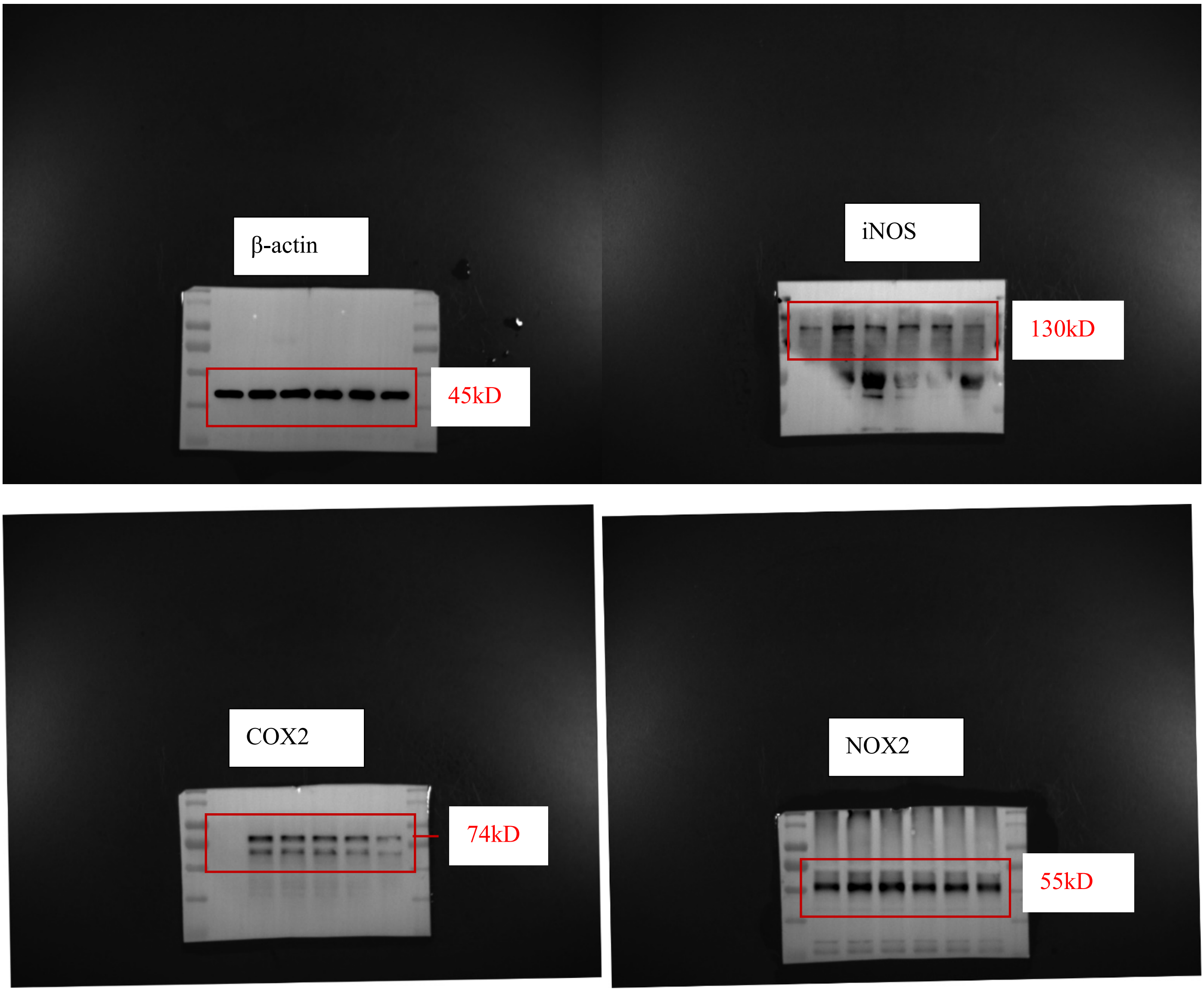

Figure S4C

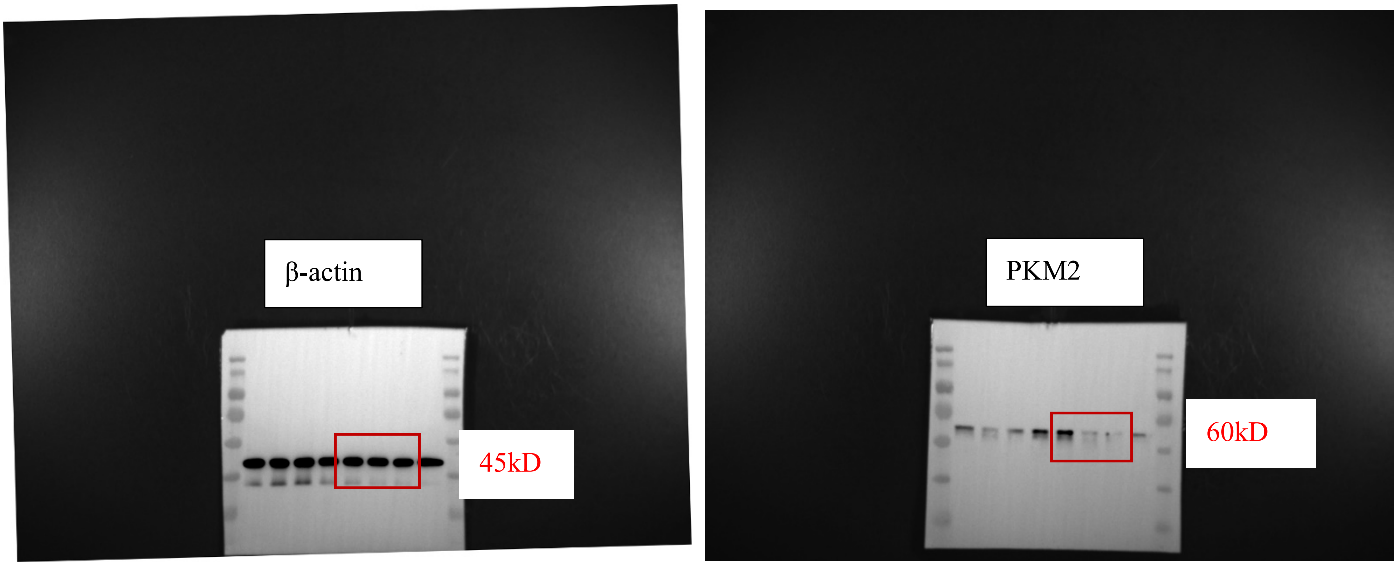

Figure S4E

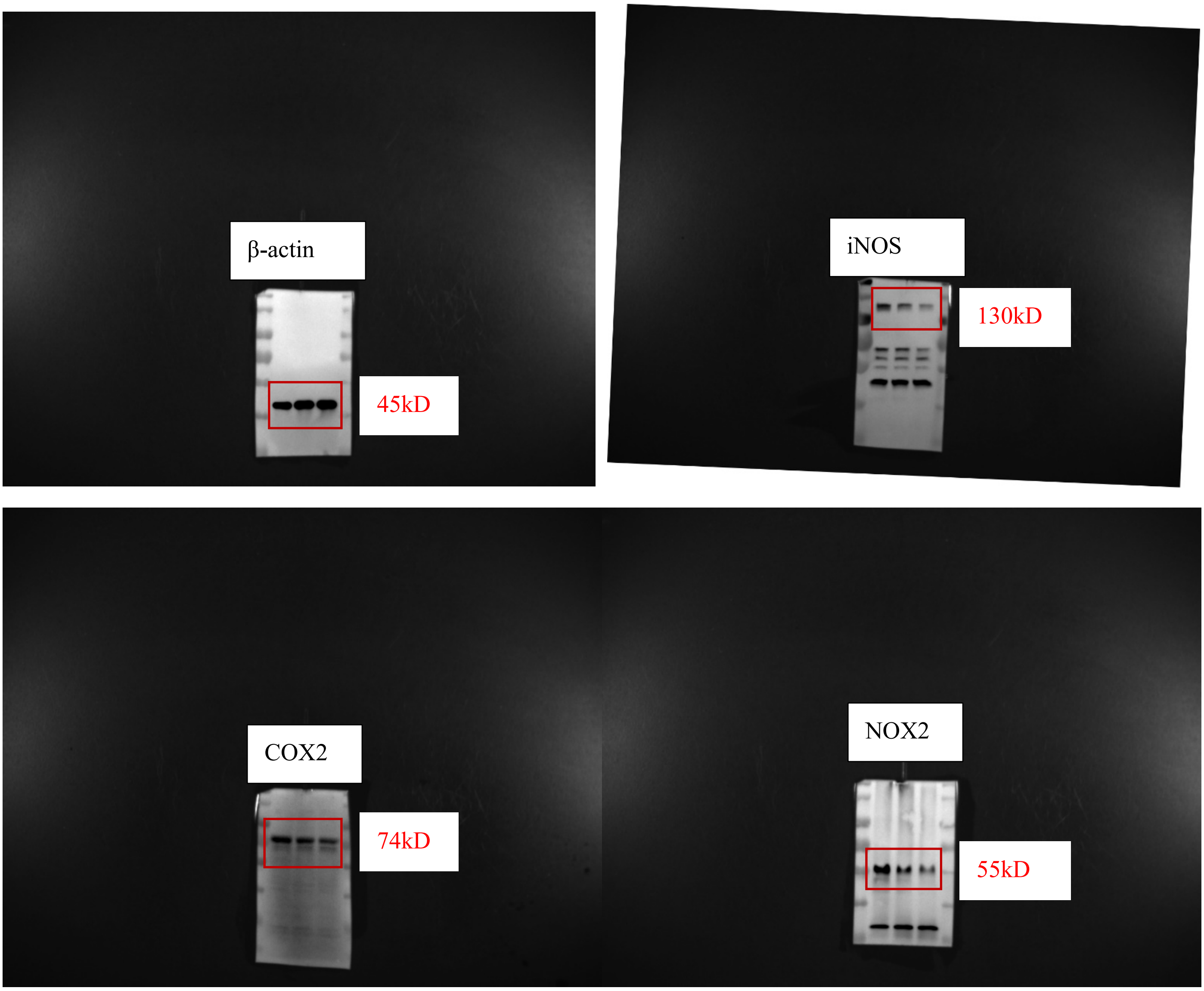

Figure S5C

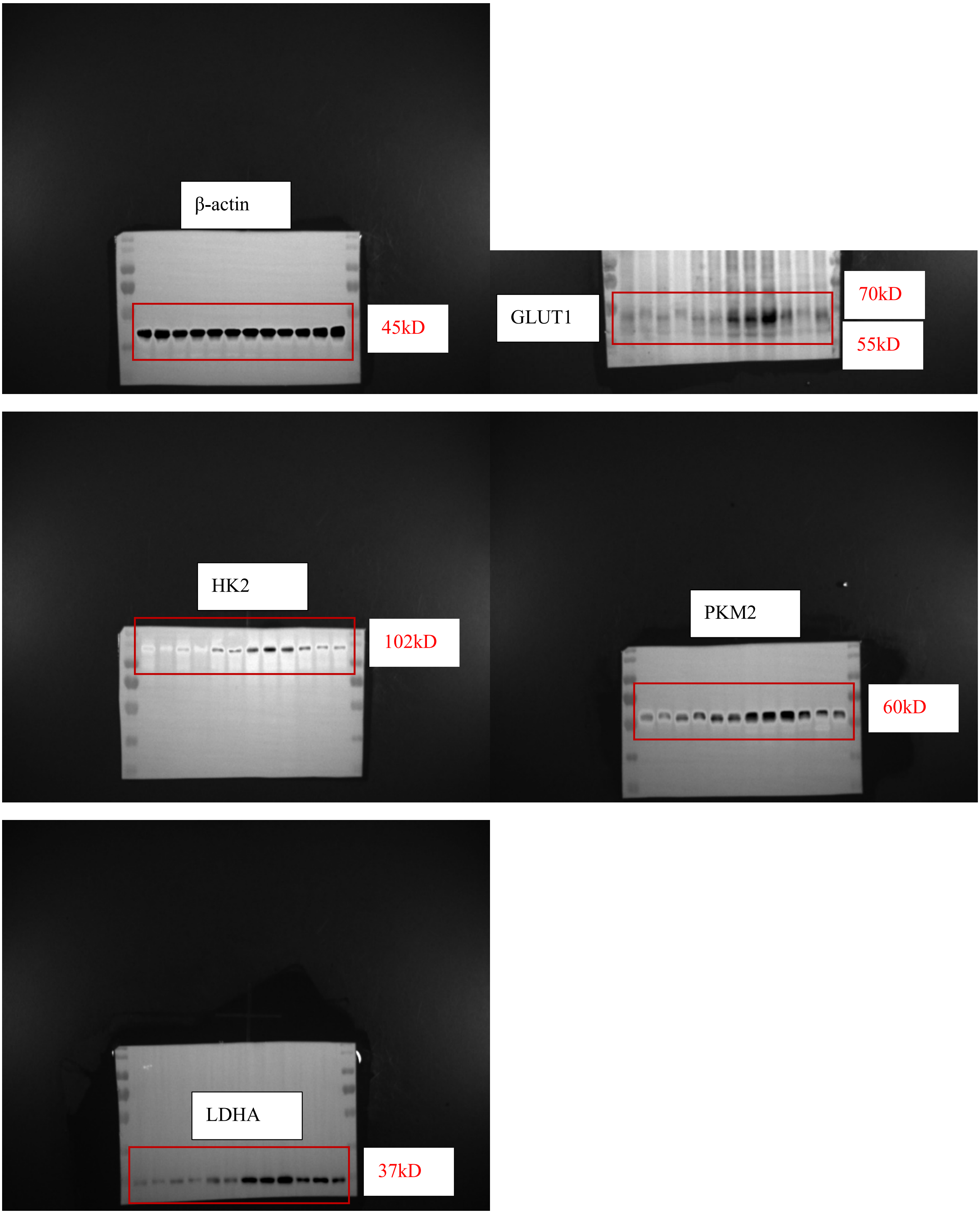

Figure S5E

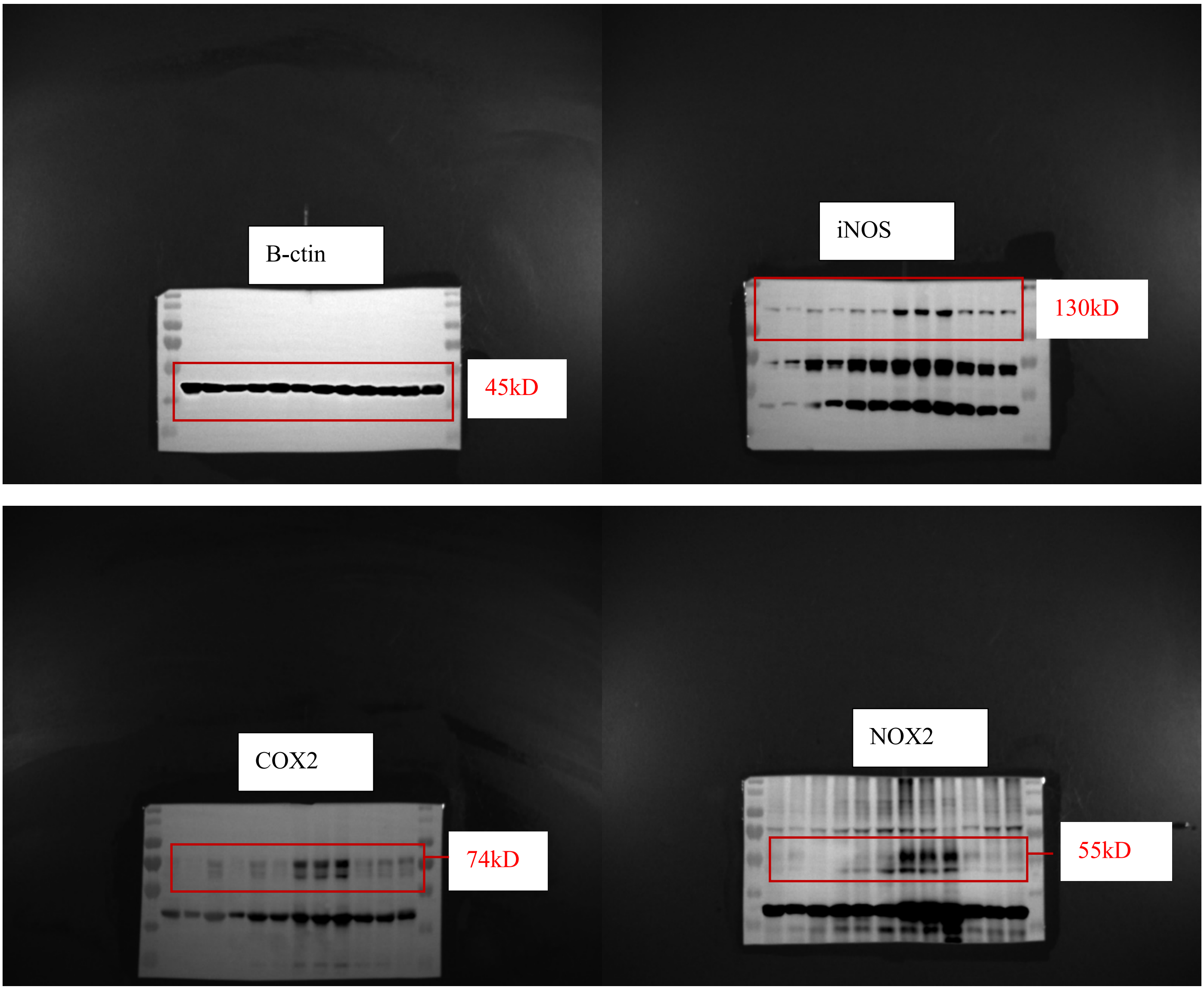

Supplement: Supplementary file 2 — Additional file 2. Raw data of this paper [file 40164_2022_334_MOESM2_ESM.pdf]
